# A revision of the New World species of *Gymnoclasiopa* Hendel (Diptera, Ephydridae)

**DOI:** 10.3897/zookeys.248.4106

**Published:** 2012-12-05

**Authors:** Wayne N. Mathis, Tadeusz Zatwarnicki

**Affiliations:** 1 Department of Entomology, NHB 169, PO Box 37012; Smithsonian Institution, Washington, D.C. 20013-7012, USA; 2 Department of Biosystematics, Opole University, ul. Oleska 22, 45-052 Opole, Poland

**Keywords:** Diptera, Ephydridae, *Gymnoclasiopa*, new species, New World

## Abstract

Species of the shore-fly genus *Gymnoclasiopa* Hendel from the New World are revised, including *Gymnoclasiopa grecorum* **sp. n.** (Alaska. Juneau: Gastineau Channel, Thane Road (S Juneau; 58°16.9'N, 134°22.4'W)) and *Gymnoclasiopa matanuska* s**p. n.** (Alaska. Matanuska-Susitna: Palmer (Matanuska River; 61°36.5'N, 149°04.1'W)). We also clarify the status of previously described species, including those now discovered to have Holarctic distributions and/or for which sexual dimorphism was not appreciated and the species was described twice, including *Gymnoclasiopa montana* (Cresson) as a **syn. n.** of *Gymnoclasiopa bohemanni* (Becker). Two species, *Gymnoclasiopa bella* (Mathis), **comb. n.,** and *Gymnoclasiopa chiapas* (Mathis), **comb. n.,** are transferred from *Ditrichophora* to *Gymnoclasiopa*, and *Gymnoclasiopa cana* Cresson **stat. rev.** and *Ditrichophora canifrons* Cresson, **stat. rev.** are returned to *Ditrichophora*, the genus in which Cresson originally described them. A neotype is designated for *Gymnoclasiopa tacoma* to stabilize the nomenclature of this species. The two excluded species, *Ditrichophora cana* and *Ditrichophora canifrons*, are diagnosed and distributional data are also provided. For all known New World species of *Gymnoclasiopa*, structures of the male terminalia are described for the first time and are fully illustrated. Detailed locality data and distribution maps are also included. To provide context and also to facilitate identification, diagnoses are included for the tribe Discocerinini and genus in addition to a key to the genera and species occurring in the New World.

## Introduction

The shore-fly genus *Gymnoclasiopa* Hendel occurs primarily in the Holarctic Region (Mathis and Zatwarnicki 1995) with some species being found jointly in both the Nearctic and Palearctic Regions. Discovering additional species common to both biogeographic regions is an objective of this study, as is unraveling any nomenclatural issues that may result when the same species was described separately in both biogeographic regions. Another impetus for this revision is to contribute to ongoing studies of the Alaskan and Mongolian faunas, among others, for which correctly determined species are essential. This revision treats congeners occurring in the New World.

Although specimens are not generally uncommon in nature, most collections, with few exceptions, have relatively few specimens, and the genus is not generally well known except to specialists. Only a few authors have reported on any included species, and aside from Cresson (1942), there are no comprehensive treatments available. The literature on included taxa is not extensive and mostly comprises alpha-level taxonomic treatments, frequently as isolated species descriptions. In view of recent field work and discoveries, the available literature is also inadequate for dealing with species that are found in both the Nearctic and Neotropical Regions. Likewise, the obvious sexual dimorphism that is now recognized in some species was not then appreciated, which contributed to confused concepts of some species and thus to incorrect determinations. We also know virtually nothing about the ecology and natural history of any of the included species except for brief habitat characterizations where adults have been found (Deonier 1965). With clarification of species--how they can be recognized and where they occur--we hope that additional research on immature stages and other aspects of their natural history and ecology will be fostered and facilitated.

Cresson (1942) provided a brief synopsis of Nearctic species. The included species, however, were treated within the genus *Ditrichophora*, which was and continued to be their accepted status until Zatwarnicki (1992) and Mathis and Zatwarnicki (1995) accorded generic status to *Gymnoclasiopa* based on characters of the male terminalia. Determining generic concepts continues to be a difficult issue because external characters, which were used exclusively in the original diagnosis of the genus, are not always wholly concordant with interpretations of structures of the male terminalia.

## Methods and materials

**Figures 1–3.**
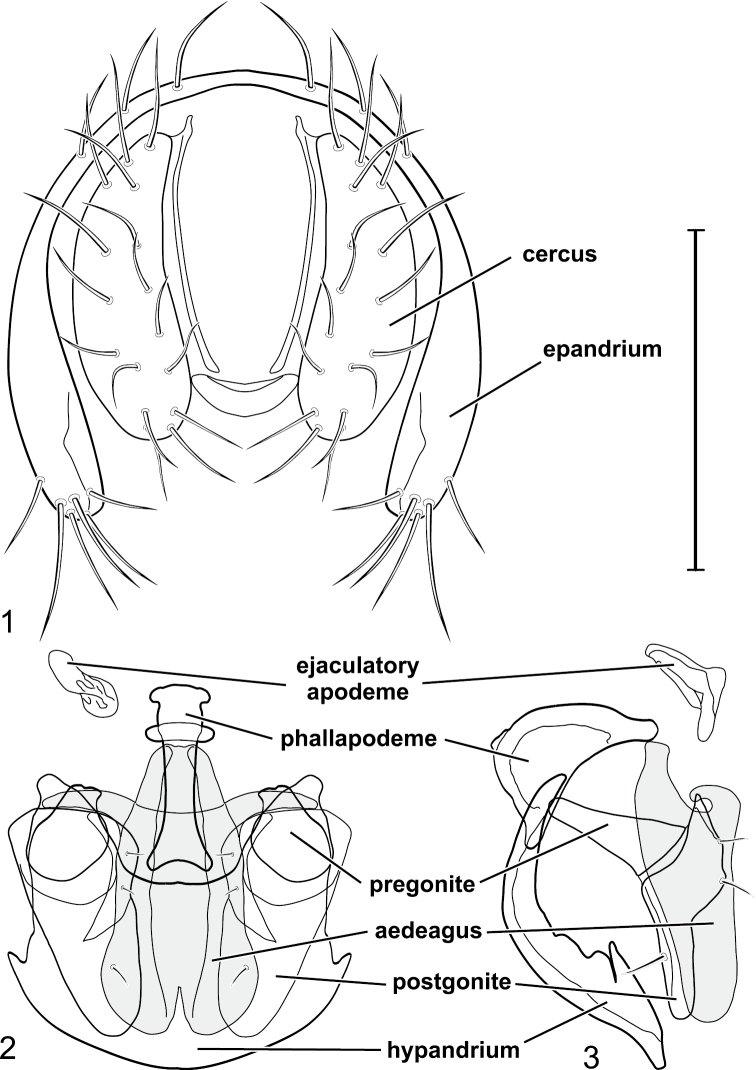
*Gymnoclasiopa argyrostoma* (Cresson) (USA: Washington. Pierce: DuPont). **1** epandrium and cerci, posterior view **2** internal structures of male terminalia (aedeagus [shaded], phallapodeme, gonite, hypandrium), ventral view **3** same, lateral view. Scale bar = 0.1 mm.

The descriptive terminology, with the exceptions noted in Mathis (1986) and Mathis and Zatwarnicki (1990a), follows McAlpine (1981). Because specimens are small, usually less than 3.5 mm in length, study and illustration of the male terminalia required use of a compound microscope. We have followed the terminology for most structures of the male terminalia that other workers in Ephydridae have used (references in Mathis 1986; Mathis and Zatwarnicki 1990a, 1990b), such as surstylus. Zatwarnicki (1996) suggested that the pre- and postsurstylus correspond with the pre- and postgonostylus and that the subepandrial sclerite is the same as the medandrium. The terminology for structures of the male terminalia is provided directly on Figs 1-3. We use the term basal flagellomere for the large antennomere beyond the pedicel. We prefer this term over “first flagellomere” as there may be more than one flagellomere involved, and basal does not imply a number or numbers. We likewise do not use “postpedicel” (Stuckenberg 1999) for this antennomere because at least the multisegmented arista is beyond the pedicel in addition to the large antennomere, and postpedicel is thus ambiguous and lacking in precision.

Dissections of male terminalia were performed following Clausen and Cook (1971) and Grimaldi (1987). Abdomens were removed with microforceps and macerated in a sodium hydroxide solution. Cleared genitalia were then transferred to glycerin for observation, description, and illustration. The dissected abdomen was placed in a plastic microvial filled with glycerin and attached to the pin supporting the remainder of the insect from which it was removed.

The species descriptions are composite and not based solely on holotypes. Head and two venational ratios used in the descriptions are based on three specimens (largest, smallest, and one other): gena-to-eye ratio – genal height (immediately below maximum eye height)/eye height; costal vein ratio – the straight line distance between the apices of R_2+3_ and R_4+5_/distance between the apices of R_1_ and R_2+3_; M vein ratio – the straight line distance along vein M between crossveins dm-cu and r-m/distance apicad of dm-cu.

Distribution maps were made using ESRI ArcView© GIS 3.2. Longitude and latitude coordinates were obtained for the locality where each specimen was collected and entered into a Microsoft Excel© spreadsheet. If unavailable directly from specimen labels, longitude and latitude were estimated using gazetteers and maps to determine the geographical coordinates. Localities of specimens were plotted on a world land projection, presented within ESRI ArcView layouts and exported as encapsulated postscript (EPS) files.

Many specimens examined for this study are in the National Museum of Natural History, Smithsonian Institution, Washington, D.C. (USNM). We also borrowed and studied numerous specimens, especially primary types from the following museums:

ANSPAcademy of Natural Sciences of Philadelphia, Pennsylvania (Jon K. Gelhaus, Donald F. Azuma, and Jason D. Weintraub)


CASCalifornia Academy of Sciences, San Francisco, California (Norman D. Penny)


MCZMuseum of Comparative Zoology, Harvard University, Cambridge, Massachusetts (Philip D. Perkins)


NMWNaturhistorisches Museum, Wien, Austria (Peter Sehnal)


NRSNaturhistoriska Riskmuseet, Stockholm, Sweden (Niklas Jönsson, Bert Vicklund and Kevin C. Holston)


UAFUniversity of Alaska, Fairbanks, Alaska (Derek S. Sikes)


WSUMaurice T. James Collection, Department of Entomology, Washington State University, Pullman, Washington (Richard S. Zack)


ZMHUZoologisches Museum, Humboldt Universität, Berlin, Germany (Joachim Ziegler)


## Systematics

### Tribe Discocerinini

Cresson

http://species-id.net/wiki/Discocerinini

Discocerinini (as Discocerini) Cresson 1925: 228. Type genus: *Discocerina* Macquart 1835. Mathis and Zuyin 1989: 435 [description, key to Asian genera]. Zatwarnicki and Mathis 2001: 1–51 [revision of genera].

#### Diagnosis.

A tribe of the subfamily Gymnymyzinae that is distinguished by the following combination of characters: Small to medium-sized shore flies, body length 1.25–3.50 mm; usually invested with considerable microtomentum, especially frons and mesonotum.

*Head*: Frontal vitta (or ocellar triangle) mostly bare of setulae, not conspicuously setulose; pseudopostocellar setae well developed, length greater than distance between either posterior ocellus and anterior ocellus, generally with proclinate orientation and slightly divergent; ocellar seta inserted anterior to lateral alignment of anterior ocellus, sometimes only slightly so; reclinate fronto-orbital seta inserted in front of proclinate fronto-orbital (if 2 proclinate fronto-orbital setae present, reclinate seta inserted in front of the larger proclinate seta); proclinate fronto-orbital seta subequal to length of reclinate seta. Pedicel bearing a large seta anterodorsally; arista with 5-7 dorsally branching rays evenly along aristal length. Compound eye bearing numerous, interfacetal microsetulae. Face generally smooth, not conspicuously pitted or rugose, in lateral view shallowly carinate between antennal bases and/or very shallowly conically produced, convex. Gena generally short (secondarily high in some species), bearing setulae (including midportion) and 1 large seta, its posterior (postgenal) margin rounded, not sharp. Oral opening and clypeus narrow; mouthparts generally dark colored; clypeus generally microtomentose, similar to microtomentum of face.

*Thorax*: Mesonotum generally microtomentose, usually densely so; supra-alar seta usually evident although sometimes reduced; acrostichal setulae arranged in about 8 irregular rows; prescutellar acrostichal setae approximate and inserted behind level of posteromost dorsocentral setae; scutellum usually moderately densely setulose, bearing more than 20 setulae, these evenly scattered; both anterior and posterior notopleural setae inserted at about the same level from notopleural/anepisternal suture; anepisternum with 2 equal setae along posterior margin. Wing with vein R_2+3_ long, extended nearly to level of apex of vein R_4+5_. Foreleg normally developed, not raptorial with greatly enlarged femur.

*Abdomen*: Five tergites visible, usually not covered with microtomentum. Male terminalia: Structures symmetrical; cerci paired, hemispherical, setose, bearing sides of rectum, sometimes fused with posteroventral margin of epandrium; epandrium U-shaped, encircling cerci, anterior margin rounded, in lateral view with setae mainly on dorsum and along anteroventral margin; presurstylus lacking or fused indistinguishably with epandrium; posterolateral arms of epandrium attached with ventral apex of gonites, middle of posterior margin a base for phallapodeme; phallapodeme situated under aedeagus, associated with hypandrium and with ventral part of base of aedeagus, ventral margin with lobate appendix providing attachment for genital muscles that move aedeagus, sometimes fused with base of aedeagus; gonites paired, connecting sides of base of aedeagus and laterodorsal margin of epandrium, bearing 1 or some setulae; subepandrial plate reduced; aedeagus tubular, tapered anteriorly; ejaculatory apodeme usually lacking, if present as a spatula against background of ductus ejaculatorius.

#### Discussion.

Starting with Cresson (1925), who first described Discocerinini, and including all students of the family until Mathis and Zuyin (1989), the diagnoses, descriptions, and catalogs of this tribe included some taxa that are not closely related phylogenetically, rendering the tribe polyphyletic. Mathis and Zuyin (1989) recharacterized Discocerinini using synapomorphies and resulting in a monophyletic tribe into which Mathis and Zatwarnicki (1995) included eight genera and 143 species in their world catalog. Zatwarnicki and Mathis (2001) added two additional genera, *Galaterina* and *Orasiopa*, and altered the status of some subgenera in their phylogenetic study of the tribe.

On a world basis, Zatwarnicki and Mathis (2001) proposed a phylogenetic hypothesis for higher-level relationships within the tribe Discocerinini. They divided the included genera into three lineages: the *Gymnoclasiopa*, *GDiclasiopa*, and *Discocerina* groups. The *Gymnoclasiopa* group, which only includes the genus *Gymnoclasiopa*, is characterized mostly by mostly plesiomorphic characters: face flat; facial setae inserted close to margin of eye; abdominal sternites simple, rectangular; pre- and postgonite separated; postgonites rounded apically, bearing 3-5 dorsal setulae. A synapomorphy for this group and genus is the shape of the aedeagus, which has lateromedial appendices (the plesiomorphic condition is an aedeagus without lateromedial appendices).

**Key to New World genera of Discocerinini**

**Table:** 

1	Notopleuron bare of setulae	2
–	Notopleuron setulose in addition to 2 large setae	6
2	Forefemur slightly enlarged, bearing distinct row of stout, short setae along apical half of posteroventral surface	*Pectinifer* Cresson
–	Forefemur normally developed, lacking row of short, stout setae along posteroventral surface	3
3	Postsutural supra-alar seta strong, distinct, longer than posterior notopleural seta. Face with dorsoclinate seta at lower lateral extremity	*Diclasiopa* Hendel
–	Postsutural supra-alar seta very short or absent, if distinguishable distinctly shorter than posterior notopleural seta. Face without dorsoclinate seta at lower lateral extremity	4
4	Hindtibia with a preapical, ventral, spur-like seta; facial series comprising 2–3 large setae, dorsal seta inserted slightly medially from other setae and arising from distinct, shiny papilla, with a small, slightly dorsoclinate seta laterad of dorsal seta; generally microtomentose, cinereous species, appearing dull	*Hecamedoides* Hendel
–	Hindtibia lacking a preapical, ventral spur-like seta; facial series comprised of 2 large setae, dorsal seta not arising from a shiny papilla and lacking a smaller seta laterad of dorsal seta; mostly bare to sparsely microtomentose, shiny to subshiny species	5
5	Face rather flattened, antennal grooves not always sharply defined ventrally; facial series of setae inserted very close to parafacial, dorsalmost seta not appreciably more removed medially than ventral seta	*Gymnoclasiopa* Hendel
–	Face rather prominent at level of dorsal facial setae, sometimes transversely carinate: antennal grooves generally sharply defined ventrally	*Ditrichophora* Cresson
6	Face with 2 or more conspicuous rows of setae/setulae on each side, paralleling facial suture setal row medial, row(s) of setulae between setal row and parafacial (Figs 10–11, 28–29)	7
–	Face with a single row of setae laterally	8
7	Face with secondary series of dorsolaterally inclined setae laterad to primary series	*Polytrichophora* Cresson
–	Face with setae and setulae of rows inclinate or ventroinclinate	*Facitrichophora* Mathis & Zatwarnicki
8	Gena and lower part of parafacial broad; lateral margin of abdomen usually with gray to whitish microtomentose areas, these usually wedge shaped	*Hydrochasma* Hendel
–	Gena and parafacial rather narrow; abdomen lacking wedge-shaped, light-colored areas laterally	9
9	Parafacial wide and bearing setulae	*Discocerina* Macquart
–	Parafacial narrow and lacking setulae	10
10	Postsutural supra-alar and prescutellar acrostichal setae greatly reduced or lacking; facial series of setae 2, these well separated, distance between them subequal to length of basal flagellomere; parafacial very narrow at anteroventral margin of eye	*Lamproclasiopa* Hendel
–	Postsutural supra-alar and prescutellar acrostichal setae present; facial series of setae 3-4, distance between setae conspicuously less than length of basal flagellomere, if 2 facial setae present, see first character; parafacial evenly wide throughout length	*Orasiopa* Zatwarnicki & Mathis

### Genus Gymnoclasiopa

Hendel

http://species-id.net/wiki/Gymnoclasiopa

Gymnoclasiopa Hendel 1930: 136 (as a subgenus of *Discocerina*). Type species: *Notiphila plumosa*Fallén 1823, by original designation. Cresson 1942: 119–121 [review, Nearctic species]. Wirth 1965: 739-740 [Nearctic catalog]. Zatwarnicki 1992: 89 [generic status]. Mathis and Zatwarnicki 1995: 174–178 [world catalog; world catalog].

#### Diagnosis.

*Gymnoclasiopa* is distinguished from other genera of the tribe Discocerinini by the following combination of characters: Small to moderately small shore flies, body length 1.70–3.30 mm; generally mostly bare to sparsely microtomentose, shiny to subshiny species. *Head*: Frons lacking orbital seta. Face moderately prominent at level of dorsal facial seta; antennal grooves generally weakly defined ventrally; face lacking secondary series of setae; facial setae 2–3, dorsal setae not arising from shiny papilla, lacking a dorsoclinate seta at lower lateral extremity; parafacial narrow throughout length, lacking setulae; gena generally low to moderate. Eye generally oval to nearly round, moderately conspicuously microsetulose, bearing interfacetal setulae not discernible by light stereomicroscope. *Thorax*: Presutural supra-alar seta well developed; postsutural supra-alar setae lacking; acrostichal setae present; notopleuron bare of setulae. Wings transparent, rarely infuscate apically; costa bearing 3–4 long, dorsal setae between humeral and subcostal breaks. Forefemur normally developed, lacking row of short, stout setae along posteroventral surface; hindtibia lacking a preapical, ventral, spurlike seta. *Abdomen*: Tergites usually unicolorous, lacking light colored areas laterally. Tergite 4 of ♂ as long or slightly than tergite 3. Male terminalia: Epandrium U-shaped in dorsal view, complete posteriorly; arms projected ventrad, posterior surface generally setulose, generally thickly formed, especially dorsal portion, arms tapered gradually toward ventral apex; cercus not fused anteriorly with epandrium, in posterior view broadly lunate, or elongate (3× longer than wide), posterior apex more narrowly pointed; pregonite variously shaped, but generally rounded apically, bearing 2-5 setulae along basoposterior margin, and 1 subapical setula along ventral margin toward hypandrium, in lateral view fusiform, in dorsal view ovate with lunate fold on 2/3 ventral margin overlapping the sides of aedeagus; postgonite more or less regularly lobate, in lateral view rod-shaped, slightly widen dorsally, ventrally it is associated with hypandrium arm, and dorsally with posteroventral margin of post-gonite; aedeagus longer than wide, mostly tubular, in dorsal view navicular, broadly rounded or bifurcate apically, bearing lateromedial projections attached to posterior margin of pregonites, in lateral view cigar shaped or ovate, or tapered toward apex; phallapodeme separate from aedeagus, in dorsal view elongate, bifurcated basally and broadly clavate at the apex; keel in lateral view variously shaped, more or less triangular or sometimes hemispherical with posterior margin curved; hypandrium in dorsal view trapezoidal, broadly rounded along anterior margin, posterolateral arms broad, posterior incision reach to 1/3 hypandrial length, in lateral view flat, sometimes irregularly, but slightly curved; ejaculatory apodeme present in form of patella, in lateral view L-shaped.

#### Distribution.

Holarctic and Neotropical Region.

#### Natural history.

Adults are found on bare, sandy to muddy shorelines of both lentic and lotic aquatic systems in primarily temperate climates. Although we have not quantified our collecting, our perception is that specimens are more common in lotic systems. Nothing is known about the immature stages, and little else is known about most other biological aspects of the included species (Zack 1983).

#### Key to species of Gymnoclasiopa from the New World

**Table:** 

1	Maxillary palpus and antenna dark; microtomentum of face distinctive, generally silver colored	2
–	Maxillary palpus yellow to red; often antenna generally yellowish; microtomentum of face generally yellow or brown	7
2	Basitarsomere of foreleg yellow	3
–	Basitarsomere of foreleg black	6
3	Mesonotum largely shiny black. Halter stem black	4
–	Mesonotum mostly microtomentose, at most subshiny. Halter stem tan to yellowish	5
4	Postpronotum and notopleuron of male generally bare of microtomentum, shiny, similar to mesonotum and anepisternum; prescutellar acrostichal setae well developed; male with anterior third of frons bare of microtomentum, shiny black	*Gymnoclasiopa chiapas* (Mathis) comb. n.
–	Postpronotum and most of notopleuron of male densely invested with fine, brown microtomentum, contrasted sharply with generally shiny, adjacent mesonotum and anepisternum; prescutellar acrostichal setae weakly developed; frons of male generally sparsely microtomentose to anterior margin	*Gymnoclasiopa bella* (Mathis) comb. n.
5	Antenna black. Postpronotum through notopleuron and ventrad to include most of anepisternum tan to grayish tan, finely microtomentose, mostly dull	*Gymnoclasiopa grecorum* sp. n.
–	Antenna reddish ventrally, especially basal flagellomere, microtomentum on basal flagellomere not visible. Postpronotum, notopleuron, and anepisternum black, subshiny, sparsely microtomentose	*Gymnoclasiopa parilis* (Cresson)
6	Thorax and abdomen generally subshiny to mostly shiny, with only sparse microtomentum dorsally. Basal tarsomeres of mid- and hindtarsi yellow, contrasted distinctly with forebasitarsomeres. Genal height subequal to height of basal flagellomere	*Gymnoclasiopa argyrostoma* (Cresson)
–	Thorax and abdomen generally densely microtomentose, gray to tannish gray. Tarsi unicolorous, grayish black. Genal height greater than height of basal flagellomere	*Gymnoclasiopa matanuska* sp. n.
7	Scape and pedicel blackish, basal flagellomere yellowish basoventrally (postpronotum and notopleuron grayer than the rest of mesonotum, similar to anepisternum and contrasted with olive, subshiny surface)	*Gymnoclasiopa subnubila* (Cresson)
–	Antenna yellowish, sometimes basal flagellomere slightly darkened dorsally	8
8	Fore- and midtibia black, similar to respective femora. Frons, mesonotum, and pleural area sparsely and thinly microtomentose, subshiny (parafacial and gena covered by grayish microtomentum, contrasted with gold-yellow color of face)	*Gymnoclasiopa tacoma* (Cresson)
–	Foretibia mostly yellow, sometimes slightly brownish medially; midtibia with apices yellow, otherwise brown to black. Frons densely but finely microtomentose	9
9	Anterolateral area of mesonotum, just mediad of area from postpronotum through notopleuron, densely microtomentose, mostly dull; lateral mesonotal area from and including postpronotum and notopleuron, densely and finely microtomentose; this area of males brown, contrasted with whitish to silvery gray microtomentum of broad, medial portion; same area in female concolorous with medial coloration. Midtibia mostly yellow but with brownish area around apical 1/3	*Gymnoclasiopa bohemanni* (Becker)
–	Anterolateral area of mesonotum, just mediad of area from postpronotum through notopleuron, thinly to very thinly microtomentose, subshiny to shiny with some metallic luster; lateral mesonotal area from and including postpronotum and notopleuron in males with thin microtomentum whitish to grayish, similar to central mesonotal microtomentum, not brown. Midtibiae darkened	*Gymnoclasiopa pulchella* (Meigen)

### Gymnoclasiopa argyrostoma

(Cresson)

http://species-id.net/wiki/Gymnoclasiopa_argyrostoma

Figs 1 [Fig F2] –16

Discocerina argyrostoma Cresson 1916: 149.
Ditrichophora argyrostoma . Cresson 1942: 121 [generic combination]; 1944: 161 [synonymy]. Wirth and Stone 1956: 467 [key, California]. Wirth 1965: 739 [Nearctic catalog]. Cole 1969: 398 [fauna, western North America]. Zack 1983: 216 [ecology].
Gymnoclasiopa argyrostoma . Mathis and Zatwarnicki 1995: 174-175 [generic combination; world catalog].
Discocerina aliena Cole and Lovett 1921: 334 [nomen nudum].
Discocerina aliena Cresson 1922: 137. Cresson 1944: 161 [synonymy].
Ditrichophora aliena . Cresson 1924: 159 [generic combination].

#### Diagnosis.

**Figures 4–15. F2:**
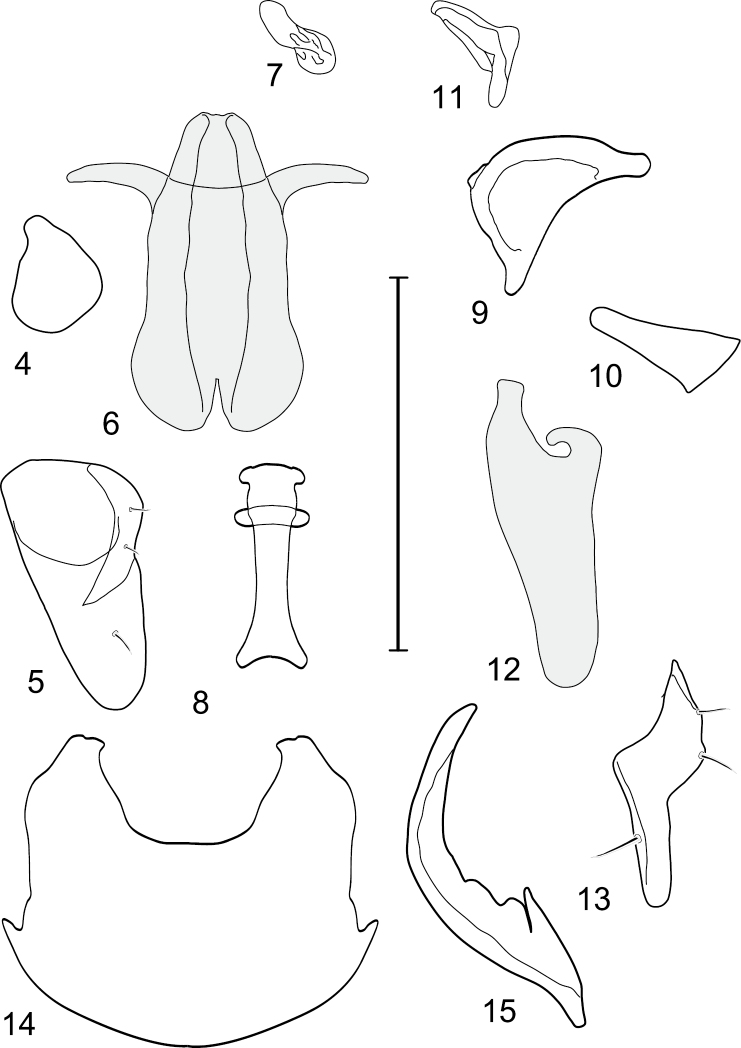
Gymnoclasiopa argyrostoma (Cresson) (USA: Washington. Pierce: DuPont). **4** pregonite, ventral view **5** postgonite, ventral view **6** aedeagus (shaded), ventral view 7 ejaculatory apodeme, ventral view **8** phallapodeme, ventral view **9** phallapodeme, lateral view **10** pregonite, lateral view **11** ejaculatory apodeme, lateral view **12** aedeagus (shaded), lateral view **13** postgonite, lateral view **14** hypandrium, ventral view **15** hypandrium, lateral view. Scale bar = 0.1 mm.

This species is distinguished from congeners by the following combination of characters: Moderately small to medium-sized shore flies, body length 2.10–3.25 mm; generally shiny black. *Head*: Frons blackish, moderately microtomentose gray; mesofrons narrowly triangular. Antenna black, basal flagellomere covered with silver microtomentum; arista with 4-6 dorsally branching rays. Face relatively flat with antennal grooves mostly inconspicuous, very shallow; facial microtomentum silvery white, sericeous; gena moderately high, genal height subequal to height of basal flagellomere; gena-to-eye ratio 0.12–0.20. Maxillary palpus black. *Thorax*: Generally subshiny to mostly shiny, only very sparsely microtomentose dorsally. Wing hyaline; costal vein ratio 0.34–0.40; M vein ratio 0.58–0.60; halter stem blackish brown; knob yellowish to whitish. Coxae, femora, and tibiae black, midtibia with some grayish microtomentum from some angles; male forebasitarsomere black; female forebasitarsomere at least yellowish basally, becoming darker apically; basal tarsomeres of mid- and hindtarsi yellow, contrasted distinctly with black male forebasitarsomere. *Abdomen*: Dorsum generally subshiny to mostly shiny, with only sparse microtomentum dorsally. Male terminalia (Figs 1–15): Epandrium in posterior view (Fig. 1) as a broadly rounded, inverted U, width of dorsal portion slightly narrower than lateral arms, lateral arms shallowly arched, in lateral view wider ventrally; cercus in posterior view (Fig. 1) semilunate, narrower dorsally than ventrally; aedeagus in lateral view (Figs 3, 12) slipper-like, base deeply and unevenly incised, posterior arm of folded over at right angle, tapered toward apex, apex moderately narrowly rounded, in ventral view (Figs 2, 6) expanded laterally from narrow base to wider apex, apical margin bilobed, narrowly incised medially, each lateral lobe as wide as aedeagal base, with thin, wing-like, narrow projections sub-basally; phallapodeme in lateral view (Figs 3, 9) more or less triangular, in ventral view (Figs 2, 8) longer than wide, bar-like with basal, sub-basal and apical crossbars, apical margin shallowly emarginate; ejaculatory apodeme in lateral view (Figs 3, 11) L-shaped, in ventral view (Figs 2, 7) peanut-like; postgonite in lateral view (Figs 3, 13) acutely pointed basally, thereafter becoming wider then apex narrowed, digitiform, bearing 2-3 setulae along basoposterior margin and 1 setula along margin toward hypandrium; pregonite in lateral view (Figs 3–4) moderately elongate, tapered, narrowed toward hypandrium, expanded toward aedeagus, aedeagal end truncate; hypandrium in ventral view (Figs 2, 14) wide, width nearly twice length, broadly and shallowly rounded along anterior margin with anterolateral, pointed, lateral projections, shallowly emarginate along posterior margin medially, in lateral view (Figs 3, 15) arched, posterior angle acute, thereafter anteriorly becoming wider, widest before anterior margin.

#### Type material.

The holotype male of *Discocerina argyrostoma* Cresson is labeled “Berkeley Hills[,] Alameda Co.[,] IV,11.’08. Cal/♂/TYPE No. 6102 Discocerina ARGYROSTOMA E. T. Cresson, Jr, [red; species number and name handwritten].” The holotype is double mounted (minuten pin in a rectangular card), is in poor condition (head and wings missing), and is deposited in the ANSP (6102).

The holotype male of *Discocerina aliena* Cresson is labeled “♂/Berkeley[,] Alameda Co, 2·23·08[,] Cal/TYPE No. 6347/Holo-TYPE Discocerina ALIENA E T Cresson Jr [red; species number and name handwritten].” The holotype is double mounted (minuten pin in a rectangular block of cord), is in excellent condition, and is deposited in the ANSP (6347).

#### Type locality.

United States. California. Alameda: Berkeley (37°52.3'N, 122°16.4'W).

**Other specimens examined.** Nearctic. CANADA. *BRITISH COLUMBIA*. Coldwater River, Merritt (50°06.8'N, 120°47.5'W; 598 m), 6 Aug 1986, P. H. Arnaud, Jr. (1♂; CAS). Creek below Bridal Veil Falls, SW Hope (49°21.3'N, 121°36.4'W), 18 Aug 1986, P. H. Arnaud, Jr. (1♂; CAS). Hope, Lake of the Woods Picnic area (49°23.1'N, 121°26.5'W), 12 Jul 1974, P. H. Arnaud, Jr. (1♂; CAS). Manning Provincial Park, Similkameen River, near road to Lightning Lake Campground (49°00'N, 119°42'W), 4 Aug 1986, P. H. Arnaud, Jr. (9♂, 6♀; CAS). Pritchard (50°41.2'N, 119°49.1'W), 13 Jul 1974, P. H. Arnaud, Jr. (2♂, 4♀; CAS). Wasa Lake (49°47'N, 115°44'W), 17 Jul 1974, P. H. Arnaud, Jr. (3♂, 12♀; CAS).

MEXICO. *BAJA CALIFORNIA*. San Pedro Martir, La Grulla (30°46.3'N, 115°13.6'W; 2105 m), 12 Jun 1953, P. H. Arnaud, Jr. (4♂, 1♀; CAS).

UNITED STATES. *ALASKA. Valdez-Cordova (Census Area)*: Chitina (61°30.9'N, 144°26.2'W), 18 Jun 1953, W. C. Frohne (1♀; WSU).

*ARIZONA*. *Cochise*: Rustler Park (31°54.2'N, 109°16.5'W), 27 Aug 1953, A. H. Sturtevant (1♂, 1♀; USNM).* Coconino*: Oak Creek, 18 km N Sedona (35°N, 111°44.3'W), 13 Apr 2003, T. Zatwarnicki, W. N. Mathis (10♂, 4♀; USNM). *Gila*: Globe (33°23.7'N, 110°47.2'W), 13 Apr 1935, A. L. Melander (1♂, 1♀; USNM). *Graham*: Pinaleno Mountains, Shannon Campground (16 km SE Junction Martijilda Creek; 32°37.5'N, 109°52.5'W; 2774 m), 28 May 1993, J. Gelhaus (1♂; ANSP). *Navajo*: Kayenta (36°44.4'N, 110°14.4'W), 14 Apr 2003, T. Zatwarnicki, W. N. Mathis (3♂, 1♀; USNM). *Pima*: Tucson, Saguaro National Park (32°16.7'N, 111°10.9'W), 10 Apr 1935, A. L. Melander (1♂; ANSP). *Yavapai*: Sedona (34°49.6'N, 111°48.3'W), 13 Apr 2003, T. Zatwarnicki, W. N. Mathis (2♂, 1♀; USNM).

*CALIFORNIA. Alameda*: Berkeley (37°52.3'N, 122°16.4'W), 21 Mar 1965, B. A. McKinley (1♂; CAS); Berkeley Hills (37°53'N, 122°14.3'W), 8 Mar 1908 (7♂, 1♀; ANSP). *Del Norte*: Gasquet (16 km W; 41°50.3'N, 124°02.1'W), 28 Jun 1972, W. N. Mathis (1♂; USNM); Panther Flat, Middle Fork Smith River (41°50.6'N, 123°56'W), 3 Jun 2009, D. and W. N. Mathis (15♂, 9♀; USNM); Wilson Creek (41°36.2'N, 124°06'W; beach), 2 Jun 2009, D. and W. N. Mathis (1♀; USNM). *El Dorado*: Fallen Leaf, Lake Tahoe (38°52.8'N, 120°04.3'W), 13 Jun 1916 (1♂; ANSP); Latrobe (3.2 km SW; 38°33.6'N, 120°59'W), 19 Feb 1974, S. Kuba (1♂; CAS). *Lassen*: Nubieber (4.8 km SW; 41°05.7'N, 121°11'W), 5 Apr 1959, M. T. James, LaMar (1♀; WSU). *Los Angeles*: Angeles Crest Highway, Arroyo Seco, Switzer Station (34°04.7'N, 118°13.6'W; 1000 m), 29 Jun 1977, P. H. Arnaud, Jr. (1♂; CAS); Los Angeles (34°03.1'N, 118°14.6'W), 26 Feb 1915, M. C. Van Duzee (1♂, 1♀; CAS); Los Cerritos (33°50.8'N, 118°09.7'W), 21 Mar 1915, M. C. Van Duzee (1♀; CAS); Pasadena (34°09.6'N, 118°01.4'W), 17 Jan 1950, A. H. Sturtevant (3♂, 1♀; USNM). *Madera*: Mark Mine, meadow near locked gate (Green Mountain; 37°34.1'N, 119°14.8'W; 2315 m), 20 Aug 1971, H. B. Leech (1♂; CAS). *Marin*: Novato, San Jose Creek (38°06.5'N, 122°34.2'W; 55 m), 18 Mar 1973, P. H. Arnaud, Jr. (1♀; CAS); Samuel P. Taylor State Park (38°01.6'N, 122°43.7'W), 8 May 1949, P. H. Arnaud, Jr. (1♂; CAS); Mill Valley (37°54.4'N, 122°32.7'W), 28 Feb 1924, M. C. Van Duzee (2♂; CAS). *Mendocino*: Russian River, near Hopland (38°58.4'N, 123°07'W), 4 May 1968, W. J. Turner (2♂; WSU). *Modoc*: Alturas (41°29.2'N, 120°32.6'W), 15 May 1948, W. W. Wirth (1♂, 1♀; CAS); Cedar Pass Campground (41°33.6'N, 120°17.6'W; 550 m), 11 Aug 1967, P. H. Arnaud, Jr. (2♂, 1♀; CAS). *Fresno*: Lake Thomas A. Edison (37°22.2'N, 118°59.2'W; 725 m), 7 Aug 1975, P. H. Arnaud, Jr. (1♂; CAS). *Marin*: Lagunitas Canyon (38°01'N, 122°44'W), 29 Mar 1908 (2♂, 3♀; ANSP). *Mariposa*: Yosemite (37°46.2'N, 119°30.6'W), 15-22 May 1908, 1916 (4♂, 5♀; ANSP). *Mono*: Leavitt Meadow (38°19'N, 119°33.1'W; 2195 m), 12 Aug 1963, H. B. Leech (1♂; CAS). *Monterey*: Paraiso Hot Springs (13 km SW Soledad; 36°19.8'N, 121°22.1'W; 400 m), 27 May 1974, P. H. Arnaud, Jr. (1♂; CAS). *Nevada*: Donner Summit (39°19.6'N, 120°23.6'W; 2200 m), 9 Sep 1977, P. H. Arnaud, Jr. (1♂, 1♀; CAS); Truckee (39°19.7'N, 120°11'W), 6 Jul 1927, E. P. Van Duzee (1♂, 1♀; CAS). *Orange*: Lower Santa Ana Canyon, Green River Camp (33°51.4'N, 117°49.1'W), 9 May 1933, E. P. Van Duzee (2♀; CAS); San Juan Capistrano Hot Spring (33°30'N, 117°39.8'W), 25 Jan 1935, A. L. Melander (1♀; WSU). *Placer*: N side Folsom Lake, Granite Bay (38°45.8'N, 121°09.8'W; Malaise trap), 26 Mar-4 Apr 1981, S. Kuba (1♂, 1♀; CAS). *Riverside*: Agua Caliente Indian Reservation, Palm Canyon (33°47'N, 116°32'W), 25 Feb 1970, P. H. Arnaud, Jr. (1♂; CAS); Andreas Canyon, Palm Springs (33°45.7'N, 116°32.2'W), 18 Jan-25 Feb 1935, 1954, P. H. Arnaud, Jr., A. L. Melander (5♂, 6♀; ANSP, CAS); Riverside (33°57.2'N, 117°23.8'W), 2 May 1935, A. L. Melander (2♂, 2♀; ANSP). *San Bernardino*: Big Bear Lake (34°14.6'N, 116°54.7'W), 24 May 1935, A. L. Melander (1♂, 1♀; USNM); China Ranch (35°48.1'N, 116°11.7'W; 380 m), 31 Mar 2005, D. and W. N. Mathis (5♂, 4♀; USNM); Forest Home (34°05.6'N, 116°55.8'W), 25 May 1935, A. L. Melander (1♂; WSU). *San Diego*: Alpine (32°50.1'N, 116°46'W), 8 Apr 1915, M. C. Van Duzee (1♂, 1♀; CAS); Palomar Observatory Campground (33°21.3'N, 116°51.7'W; 1524 m), 26 Jun 1968, P. H. Arnaud, Jr. (1♂; CAS). *San Francisco*: Lake Merced (37°43.3'N, 122°29.7'W), 20 Mar 1966, P. H. Arnaud, Jr. (2♀; CAS). *San Mateo*: Stanford University (37°24'N, 122°14.5'W), 14 Feb 2006, P. H. Arnaud, Jr. (4♂, 5♀; USNM). *Shasta*: Old Boundary Campground (40°31.4'N, 121°28.8'W), 29 Jul 1974, P. H. Arnaud, Jr. (1♂, 1♀; CAS); Honn Creek Campground (29 km SE Hat Creek; 40°46.8'N, 121°30.1'W), 30 Jul 1974, P. H. Arnaud, Jr. (2♂, 1♀; CAS); Manzanita Lake, Mount Lassen National Park (40°32.1'N, 121°34.1'W), 6 Jun 1991, J. P. Pishe (1♀; CAS). *Siskiyou*: Poker Flat (34 km NW Happy Camp; 41°55.9'N, 123°32.8'W; 1535 m), 13 Aug 1966, H. B. Leech (1♂, 1♀; CAS); McBride Springs, Mount Shasta (7 km NE Mt. Shasta City; 41°21.2'N, 122°16.9'W), 28 Jul 1974, P. H. Arnaud, Jr. (1♂; CAS). *Sonoma*: Kenwood, Sonoma Creek, Mortons Warm Springs (38°24'N, 122°33.1'W; 105 m), 18 May 1974, H. B. Leech (1♂; CAS). *Tuolumne*: Dardanelle, Middle Fork Stanislaus River (38°20.5'N, 119°50'W; 1765 m), 1 Jul 1977, P. H. Arnaud, Jr. (1♀; CAS); South Fork Stanislaus River (37°53'N, 120°25.4'W), 3 May 1964, C. D. McNeill (1♂; CAS).

*COLORADO. Boulder*: Boulder Creek (16 km W Boulder; 40°0.3'N, 105°24.4'W; 2280 m), 8 Aug 1973, P. H. Arnaud, Jr. (2♂, 1♀; CAS); Nederland (39°57.7'N, 105°30.6'W), 24 Jun 1961, G. C. Steyskal (1♀; USNM). *Chaffee*: South Fork Arkansas River, Garfield (38°33.1'N, 106°17.5'W; 2990 m), 12 Aug 1973, P. H. Arnaud, Jr. (2♂; CAS). *Dolores*: Cahone (9 km N; Dolores River; 37°38.9'N, 108°44.1'W; 1955 m), 3 Aug 2007, D. and W. N. Mathis (4♂, 3♀; USNM).

*IDAHO. Boundary*: Dawson Lake (48°46.3'N, 116°14.3'W; 885 m), 3 Jun 2006, W. N. Mathis (1♂, 4♀; USNM); Solomon Lake (48°47.6'N, 116°06.1'W; 825 m), 3 Jun 2006, W. N. Mathis (4♂, 2♀; USNM). *Custer*: Selway Wilderness, Cove Lake (44°06.1'N, 114°36.3'W; 1950 m), 22 Jul 1978, R. S. Zack (1♀; WSU). *Kootenai*: Beauty Bay on Coeur d’Alene Lake (47°36.8'N, 116°41.1'W; 686 m), 14 Jul 1978, W. J. Turner (1♂, 2♀; WSU). *Latah*: Big Meadow Creek Recreation Area (8 km NW Troy; 46°47.8'N, 116°48.8'W), 31 Jul 1979, R. S. Zack (8♂, 27♀; WSU); Laird Park (6.4 km NE Harvard; 46°56.5'N, 116°38.8'W), 24 May-6 Jul 1978, 1985, R. S. Zack (4♂, 3♀; WSU); Lost Creek, Harvard (6.4 km NNE; 46°57.7'N, 116°41.2'W), 18-30 May 1980, 1981, R. S. Zack (35♂, 16♀; WSU); Moscow Mountain (46°48.2'N, 116°52.1'W), 2 Jun 1908 (3♂, 2♀; ANSP); Moscow Mountain, West Twin Peak (46°48.2'N, 116°54.9'W), 15 Jun 1982, R. S. Zack (1♂, 1♀; WSU); Moscow Mountain, East Twin Peak (46°48.2'N, 116°52.1'W), 19 Jul 1983, R. S. Zack (10♂, 18♀; WSU); Strychnine Creek (10 km NE Joe National Forest; 46°58.7'N, 116°37.1'W), 29 Apr-4 Jun 1980, R. S. Zack (2♂, 5♀; WSU); Troy (46°44.2'N, 116°46.2'W), 31 May 1908 (1♂, 2♀; ANSP, USNM, WSU). *Oneida*: Malad (42°11.5'N, 112°15'W), 13 Aug 1950, A. H. Sturtevant (2♂, 1♀; USNM). *Nez Perce*: Black Pine Camp (38 km SE Lewiston; 46°15'N, 116°46'W), 22 Jul 1974, P. H. Arnaud, Jr. (1♂, 2♀; CAS); Lake Waha (46°12.6'N, 116°50.8'W), 9 Jun 1918, A. L. Melander (1♀; ANSP).

*MONTANA. Lake*: Flathead Lake (47°53.2'N, 114°07.9'W), 19 Aug 1916, A. L. Melander (1♂; ANSP). *Mineral*: St. Regis (37°53'N, 122°14.3'W), 28 Aug 1981, P. H. Arnaud, Jr. (1♀; CAS). *Missoula*: Brewster Creek, Clinton (46°46.4'N, 113°42.6'W), 1 Jun 1957, H. R. Dodge (1♂; USNM).

*NEVADA. Clark*: Las Vegas Wash (36°05.2'N, 114°58.8'W), 10-11 May 2001, D. and W. N. Mathis (2♂; USNM); Red Rock Canyon (36°09.6'N, 115°31.1'W), 12 May 2001, D. and W. N. Mathis (2♂; USNM). *Nye*: Toiyabe National Park, Indian Creek, Indian Valley (6.4 km ESE Grantsville; 38°50'N, 117°30'W; 2065 m), 22 Jun 1995, J. K. Gelhaus (1♀; ANSP).

*NEW MEXICO. Bernalillo*: E slope Sandia Mountains, 14 km W Cedar Crest (35°06.5'N, 106°23'W; 2438 m), 15 Jul 1979, P. H. Arnaud, Jr. (3♂; CAS); *Catron*: Pueblo Creek and Park Campground (27 km S Luna; 33°37.5'N, 109°W; 2100 m), 30 May 1993, J. K. Gelhaus (3♂; ANSP). *Cibola*: Bluewater Lake State Park (35°18.1'N, 108°06.6'W; 2220 m), 9 Aug 2007, D. and W. N. Mathis (1♀; USNM). *Grant*: Apache Creek (33°09.1'N, 108°08.9'W), 13 Jun 1950, A. H. Sturtevant (1♀; USNM); Mimbres River (NM Hwy. 61 and Royal John Mine Road; 32°43.8'N, 107°52'W; 1665 m), 1 Aug 2008, D. and W. N. Mathis (17♂, 4; USNM); Mimbres River (NM Hwy. 35; 19 km N, Hwy 152; 32°56.9'N, 108°01.1'W), 2 Aug 2008, D. and W. N. Mathis (4♂; USNM); Silver City (32°46.2'N, 108°16.8'W), 6 Aug 1950, A. H. Sturtevant (4♂, 1♀; USNM). *Luna*: Hyatt Farm (12 km NW Florida; 32°26'N, 107°35'W; stock pond), 4 Aug 1965, H. B. Leech (2♀; CAS). *Sandoval*: Cuba (18 km S; 35°58.8'N, 106°59.1'W), 25 Jun 1973, W. N. Mathis (2♀; USNM); Jemez Springs (35°46.6'N, 106°41.4'W), Jun (1♂; ANSP); Jemez Springs (12 km N; 35°48'N, 106°41.4'W), 25 Jun 1973, W. N. Mathis (9♂; USNM); La Cueva (Junction of Highways 126 & 4; 35°52'N, 106°38.4'W; 2342 m), 14 Jun 2011, D. and W. N. Mathis (1♀; USNM); Las Huertas (9 km NW San Antonito (35°18.8'N, 106°24.1'W; 2300 m), 26 May 1991, J. Gelhaus (1♂; ANSP); Valles Caldera National Preserve, Alamo Canyon (PH 2 pond; 35°55.1'N, 106°35.7'W; 2610 m), 4 Aug 2008, D. and W. N. Mathis (5♂; USNM); Valles Caldera National Preserve, Rincon de los Soldados (stock pond 2; 35°54.6'N, 106°25.3'W; 2727 m), 6 Aug 2008, D. and W. N. Mathis (2♂, 2♀; USNM); Valles Caldera National Preserve, Sulphur Creek (35°54.9'N, 106°36.4'W; 2588 m), 4 Aug 2008, D. and W. N. Mathis (2♂; USNM). *San Miguel*: Cowles (4 km S: Mora and Pecos Rivers; 35°48'N, 105°39.6'W; 2408 m), 12 Jul 1979, T. W. Davies (1♀; CAS). *Torrance*: Gran Quivira (34°15.8'N, 106°06.1'W), 8 Aug 1965, H. B. Leech (3♂; CAS).

*OREGON. Baker*: Goose Creek (35 km E Baker City; 44°49.2'N, 117°27.79'W; 825 m), 7 Jun 2006, D. and W. N. Mathis (1♂, 6♀; USNM). *Benton*: Cary’s Grove (44°22.6'N, 123°36.1'W), 2 Sep 1974, W. N. Mathis (1♂, 3♀; USNM); Corvallis (44°33.9'N, 123°15.7'W), 12 May 1930, J. Wilcox (2♂, 2♀; ANSP); Corvallis (Oregon State University sheep farm; 44°33.9'N, 123°15.7'W), 29 Mar 1972, W. N. Mathis (1♂; USNM); Corvallis (1.6 km SE, Willamette River; 44°31.7'N, 123°15.2'W), 4 Apr 1972, W. N. Mathis (9♂, 11♀; USNM); William L. Finley National Wildlife Refuge (16 km S Corvallis; 44°24.6'N, 123°19.1'W), 29 Apr 1972, W. N. Mathis (6♂, 2♀; USNM); Kiger Island (44°30.7'N, 123°14'W), 12 May 1930, J. Wilcox (1♂; ANSP); McDonald Forest (44°38'N, 123°17.6'W), 22 Jul 1974, W. N. Mathis (1♂; USNM); Philomath (1.6 km SW; 44°32.1'N, 123°23.6'W), 29 May 1972, W. N. Mathis (10♂, 8♀; USNM); Rock Creek (6.5 km SW Philomath; 44°30'N, 123°26.5'W), 6-29 May 1972, W. N. Mathis (5♂, 2♀; USNM); Willamette Park (44°32.3'N, 123°15'W), 3 May 1972, W. N. Mathis (7♂, 6♀; USNM). *Crook*: Cougar Campground, Marks Creek (44°26.9'N, 120°27.9'W), 24 Jul 1974, P. H. Arnaud, Jr. (2♂; CAS). *Deschutes*: Bend (44°03.5‘N, 121°18.9‘W), 8 Aug 1951, A. H. Sturtevant (2♂; USNM); E side McKenzie Pass (44°15.6'N, 121°48'W; 1585 m), 25 Jul 1974, P. H. Arnaud, Jr. (32♂, 37♀; CAS); Cline Falls State Park, W Redmond (44°16.1‘N, 121°15.3‘W), 25 Jul 1974, P. H. Arnaud, Jr. (1♀; CAS). *Douglas*: Elbow Lake (43°47.2'N, 124°08.9'W), 26 May 1972, W. N. Mathis (1♀; USNM); S fork Umqua River, Charles V. Stanton Park (42°56.8'N, 123°17.6'W), 21 Jun 1974, P. H. Arnaud, Jr. (1♂; CAS). *Gilliam*: 30-mile Creek (45°05.3'N, 120°14.9'W), 20 Jun 1954, J. J. Davis (1♀; WSU). *Harney*: Fields (11 km NW; 42°17'N, 118°42'W; mud pond), 29 Aug 1983, R. S. Zack (1♂; WSU); Page Springs Campground, Blitzen River (42°47'N, 118°51'W; 1300 m), 6 Aug 2005, D. and W. N. Mathis (2♂, 1♀; USNM); Juniper Lake (9.6 km SW Route 78; 42°55.3'N, 118°20.3'W), 12 Sep 1981, R. S. Zack (1♂; WSU); Steens Mountain, Big Indian Gorge (north side; 42°49'N, 118°35'W; 2730 m), 6 Aug 2005, D. and W. N. Mathis (2♂, 1♀; USNM). *Hood River*: Mt. Hood (45°24.7'N, 121°34.9'W), 25 Jun 1935, A. L. Melander (1♀; ANSP). *Jackson*: N side Siskiyou Summit, small falls, 5 km from California (42°04'N, 122°48'W), 28 Jul 1974, P. H. Arnaud, Jr. (2♂; CAS); Squaw Lakes (42°02.2'N, 123°01.4'W), 22 May 1964, J. Schuh (1♂; WSU); Talent, Bear Creek (42°14.7'N, 122°47.3'W), 28 Jul 1974, P. H. Arnaud, Jr. (1♀; CAS). *Josephine*: Merlin (3.2 km W; 42°31.2'N, 123°27.6'W), 13 May 1972, W. N. Mathis (1♂, 1♀; USNM); Selma (16 km W; 42°16.8'N, 123°38.7'W), 13 May 1972, W. N. Mathis (1♀; USNM); Rough and Ready Creek State Park (42°05.8'N, 123°40.9'W), 13 May 1972, W. N. Mathis (1♀; USNM); Whiskey Creek Campground (42°39.5'N, 123°38.2'W; 1460 m), 27 Jul 1974, P. H. Arnaud, Jr. (3♂; CAS). *Klamath*: Crater Lake (42°57.8'N, 122°08.9'W), 16 Sep 1934, A. L. Melander (1♂, 2♀; ANSP); Klamath Falls (Geary Canal; 42°13.7'N, 121°46'W), 27 Apr 1964, J. Schuh (1♀; WSU). *Lane*: Salt Creek Falls Campground (33 km SE Oakridge; 43°36.6'N, 122°07.6'W; 1219 m), 26 Jul 1974, P. H. Arnaud, Jr. (11♂, 1♀; CAS); Yachats (10.5 km S; 44°17'N, 124°06.5'W), 27 May 1972, W. N. Mathis (8♂, 8♀; USNM). *Lincoln*: Waldport (10.5 km E; 44°24.3'N, 123°56.5'W), 27 May 1972, W. N. Mathis (3♂, 4♀; USNM). *Marion*: Keizer Bottom (44°59.3'N, 123°01.3'W), 5 Mar 1940, R. E. Rieder (1♂, 1♀; WSU). *Morrow*: Willow Creek (SE Heppner; 45°19.6'N, 119°24.6'W), 18 Jun 1991, R. S. Zack (2♂, 2♀; WSU). *Multnomah*: Multnomah Falls (45°34.5'N, 122°06.9'W), 26 Jun 1974, P. H. Arnaud, Jr. (1♂; CAS). *Polk*: Sarah Helmick State Park (44°46.8'N, 123°13.8'W), 20 Mar 1972, W. N. Mathis (2♂; USNM). *Umatilla*: Ukiah-Dale Forest Wayside, Camas Creek (45°07.5'N, 118°58.3'W), 24 Jul 1974, P. H. Arnaud, Jr. (3♂; CAS). *Union*: La Grande (22.5 km S; 45°15.1'N, 118°01.5'W; 1305 m; Malaise trap), 17-23 Aug 1975, E. J. Davis (2♂; WSU); Velvet Creek (45 km SE Union; 44°59.8'N, 117°36.5'W; 1438 m; Malaise trap), 6 Jul-31 Aug 1975, 1976, E. J. Davis (2♀; WSU); Whiskey Creek (37 km SSW La Grande; 45°14.5'N, 118°12.7'W; 1560 m; Malaise trap), 22-28 Jun 1975, E. J. Davis (1♂, 1♀; WSU). *Wallowa*: Wallowa Lake State Park (45°17'N, 117°12.7'W), 18 Aug 1954, H. B. and M. T. James (1♀; WSU). *Washington*: Forest Grove (45°31.2'N, 123°06.6'W), 3 Jun 1918, F. Cole (1♂; ANSP).

*SOUTH DAKOTA. Lawrence*: Savoy (3.2 km W; 44°21.1'N, 103°56.2'W), 19 Jun 1968, W. N. Mathis (1♀; USNM).

*UTAH. Carbon*: Deadman Caynon (16 km NE Price; 39°41.7'N, 110°44'W; 2055 m), 14 Aug 2008, D. and W. N. Mathis (9♂, 3♀; USNM); Soldier Canyon (39°44.3'N, 110°36'W), 25 Aug 1972, W. N. Mathis (1♀; USNM). *Garfield*: Alvey Wash (8.4 km S Escalante; 37°42.3'N, 111°37.6'W; 1880 m), 21 May 2001, D. and W. N. Mathis (1♂; USNM); Deer Creek (37°51.2'N, 111°21.1'W; 1762 m), 21 May 2001, D. and W. N. Mathis (2♂, 1♀; USNM). *Grand*: Thompson Spring (8.9 km N Thompson Springs; 39°02.3'N, 109°43.4'W; 1740 m), 1-16 Aug 2007, 2008, D. and W. N. Mathis (6♂; USNM). *Kane*: Coral Pink Sand Dunes (37°2.8'N, 112°40.7'W; spring), 16 May 2001, D. and W. N. Mathis (3♂, 1♀; USNM); Drip Tank Canyon (37°19.4'N, 111°31.8'W), 15 May 2001, D. and W. N. Mathis (1♂, 1♀; USNM); East Fork of Virgin River (26.6 km N Kanab; 37°12.2'N, 112°41.4'W) 18 May 2001, D. and W. N. Mathis (4♂; USNM); Kanab Canyon (6.5 km N Kanab; 37°08.7'N, 112°32.4'W), 14 May 2001, D. and W. N. Mathis (2♂; USNM); Seaman Wash (24.5 km E Kanab; 37°07'N, 112°15'W), 14 May 2001, D. and W. N. Mathis (8♂; USNM); Sheep Creek (37°29.7'N, 112°04'W), 17 May 2001, D. and W. N. Mathis (6♂, 2♀; USNM); S of Right Hand Collett Canyon (37°8.1'N, 111°32'W; stock pond), 15 May 2001, D. and W. N. Mathis (1♂; USNM); White House Spring (71 km E Kanab; 37°04.8'N, 111°53.4'W; 1250 m) 19 May 2001, D. and W. N. Mathis (3♂; USNM). *Salt Lake*: Draper (40°31.6'N, 111°55.1'W; Jordan River; 1320 m), 10 May 2007, D. and W. N. Mathis (10♂, 5♀; USNM). *Utah*: Alpine Loop (Timp Lodge; 40°23.3'N, 111°35.1'W; 1930 m), 7-9 Jun 2011, D. and W. N. Mathis (2♂, 3♀; USNM); Goshen Warm Springs (7 km W Santiquen; 39°58'N, 111°56'W), 9 Jun 2011, D. and W. N. Mathis (1♂; USNM); Thistle (40°0.4'N, 111°29.7'W; 1530 m), 11 May 2007, D. and W. N. Mathis (1♀; USNM). *Washington*: Santa Clara (10 km W; 37°08.2'N, 113°41'W), 5 Apr 1968, W. N. Mathis (2♂, 1♀; USNM); Zion National Park (37°12.8'N, 112°59.8'W), 29 Apr 1935, A. L. Melander (3♂; ANSP).

*WASHINGTON. Adams*: Paha, McElroy Lake (47°01.4'N, 118°29.4'W), 20 Jul 1920, R. C. Shannon (1♀; WSU). *Asotin*: Fields’ Spring State Park (46°04.9'N, 117°10.2'W; Malaise trap), 14 Jun-31 Jul 1971, 1972, W. J. Turner (2♂, 3♀; WSU); Fields’ Spring State Park, Anatone (6.5 km S; 46°08.1'N, 117°07.9'W; 1065-1220 m), 7-12 Jun 1973, W. J. Turner (3♂, 6♀; WSU). *Benton*: Hanford Site. ALE, Lower Snively Spring (46°27.5'N, 119°43.3'W), 29 Mar-16 May 1994, 1995, R. S. Zack (20♂, 15♀; WSU); Hanford Site, ALE, Rattlesnake Ridge (46°28.6'N, 119°32.1'W), 15 May-12 Aug 1994, 1995, R. S. Zack (8♂, 5♀; WSU); Hanford Site. ALE, Snively Ranch (T11N, R25E, Sec. 8; 46°27.1'N, 119°42.9'W), 11 Apr 1994, R. S. Zack (1♂; WSU); West Richland (46°18.3'N, 119°21.7'W), 20 Jun 1973, N. E. Woodley (1♀; WSU). *Chelan*: Lake Wenatchee (47°49.4'N, 120°46.6'W), 4 Aug 1951, A. H. Sturtevant (2♂, 2♀; USNM). *Columbia*: Tucannon Fish Hatchery (32 km E Dayton; 46°33.3'N, 118°10.4'W), 15-22 May 1979, W. J. Turner, R. S. Zack (5♀; WSU). *Grant*: Crab Creek (4.8 km E Beverly; 46°50'N, 119°52'W), 30 May 2006, D. and W. N. Mathis (4♂; USNM). *Kittitas*: Vantage (Columbia River; 46°56.5'N, 119°59.1'W), 29 May 2006, D. and W. N. Mathis (4♂, 2♀; USNM). *Lewis*: La Wis Wis Campground, Ohanapecosh River (46°40.4'N, 121°35.3'W), 3 Jul 1974, P. H. Arnaud, Jr. (6♂, 8♀; CAS). *Okanogan*: Salmon Meadows (14.5 km NW Conconully; 46°42.2'N, 117°28.2'W; 1370 m), 23-26 Jul 1975, W. J. Turner (1♂; WSU); Sweat Creek Campground (3.2 km W; 46°42.2'N, 117°28.2'W; Malaise trap), 7 Jul 1972, W. J. Turner (1♂; WSU); Wauconda (8 km E; 48°43.6'N, 119°W; Rt. 30), 7 Jul 1972, W. B. Garnett (1♂; WSU); Wauconda (11.25 km E; 48°43.7'N, 118°57.9'W; Rt. 30), 29 Jul 1973, W. J. Turner (1♀; WSU); Winthrop (22.5 km N; near Chewack Campground; 48°32'N, 120°11.2'W; Malaise trap), 19 Jul 1972, W. J. Turner (1♀; WSU). *Pierce*: Brown’s Point (47°18'N, 122°26.5'W), 6 May 1971, W. N. Mathis (1♂; USNM); DuPont (5 km WSW; 47°04.3'N, 122°42.2'W), 23 Apr-9 Jun 1971, W. N. Mathis (11♂, 4♀; USNM); Gig Harbor (47°19.8'N, 122°34.8'W), 1 May 1971, W. N. Mathis (1♂ USNM); Lake Spanaway (47°06.6'N, 122°26.6'W), 15 May 1971, W. N. Mathis (1♀; USNM); Mount Rainier National Park, Cayuse Pass (46°52'N, 121°32.3'W; wet meadow; 1435 m), 25 Jun-8 Aug 1979, R. S. Zack (2♂; WSU); Mount Rainier National Park, Chinook Pass (0.8 km SW; 46°52.3'N, 121°31'W; 1615 m), 12 Sep 1978, R. S. Zack (6♂, 2♀; WSU); Mount Rainier National Park, Christine Falls (above; 46°46.9'N, 121°46.8'W; 1125 m), 11-13 Aug 1977, R. S. Zack (9♂, 7♀; WSU); Mount Rainier National Park, Comet Falls Trail above Van Trump Creek (46°46.4'N, 121°46.8'W; 1370 m), 11-13 Aug 1977, R. S. Zack (2♂; WSU); Mount Rainier National Park, Ghost Lake (46°52.4'N, 121°32.5'W; 1340 m), 8 Aug 1979, R. S. Zack (26♂, 21♀; WSU); Mount Rainier National Park, Shadow Lake near Sunrise (46°54.7'N, 121°39.4'W), 10 Aug 1977, R. S. Zack (11♂, 18♀; WSU); Mount Rainier National Park, Sunshine Point (6 km NE; 46°44.6'N, 121°55.6'W; campground), 26 Jun-8 Aug 1979, R. S. Zack (35♂, 25♀; WSU); Mount Rainier National Park, West End Road (5 km N; 46°55'N, 121°35'W), 12 Aug 1977, R. S. and V. L. Zack (1♀; WSU); Mount Rainier National Park, White River (47°12'N, 122°15.5'W; campground; 1240 m), 12 Jul-10 Aug 1977, 1978, R. S. Zack (15♂, 13♀; WSU); Mt. Rainer National Park, White River (47°12'N, 122°15.5'W), 28 Aug 1934, A. L. Melander (2♂; USNM); Mount Rainier National Park, White River (4.5 km NE; 47°13'N, 122°15'W; 1465 m), 12 Jul 1978, R. S. Zack (1♂; WSU); Tacoma (47°15.2'N, 122°26.7'W), 1 Apr 1971, W. N. Mathis (1♂, 1♀; USNM). *Skamania*: Randle (51.5 km SE; 46°21.2'N, 121°47.3'W; Malaise trap), 29 Jul 1972, W. B. Garnett, W. J. Turner (1♂; WSU). *Spokane*: Spokane (47°39.5'N, 117°25.6'W), 27 Jun-30 Jul 1920, 1954, A. L. Melander, A. H. Sturtevant (1♂, 1♀; USNM); Spokane State Park, Bald Knob Campground (47°53.5'N, 117°07.4'W; Malaise trap; 1450-1585 m), 25 Jun-10 Jul 1975, 1976, 1979, W. J. Turner (6♂, 19♀; WSU). *Stevens*: Deer Lake (8 km NE; 48°29'N, 117°42'W; 975 m), 18 Jul 1975, W. J. Turner (4♀; WSU); Deer Lake near Chewelah (48°06.7'N, 117°35.2'W), 30 Jun 1972, M. T. James (1♂; WSU). *Whitman*: Albion (46°47.5'N, 117°15'W), 27 May (1♀; WSU); Almota (46°42.2'N, 117°28.2'W), 23 Apr-2 Jun 1918, 1955, 1972, M. T. James, A. L. Melander, W. J. Turner (56♂, 15♀; ANSP, USNM, WSU); Bald Butte (11 km SE Pullman; 46°38'N, 117°05.3'W), 12 May 1973, L. C. Wright (2♀; WSU); Lyle Grove Biological Area (13 km SW Pullman; 46°42'N, 117°12'W; 645 m), 7 Apr-18 Jun 1976, 1978, 1979, W. J. Turner, R. S. Zack (52♂, 25♀; WSU); Pullman (46°43.9'N, 117°10.8'W; Malaise trap), 15 Apr-31 Jul 1965, 1970, R. D. Akre, J. Novak, J. A. Quist, H. S. Telford (2♂, 4♀; WSU); Pullman (46°43.9'N, 117°10.8'W; golf course), 6 Apr-13 Sep, 1908, 1909,1912, 1923, 1965, 1970, 1971, R. D. Akre, A. L. Melander, J. Novak, J. A. Quist, H. S. Telford, W. J. Turner (10♂, 16♀; WSU); Steptoe Canyon (11 km SW Colton; 46°27.3'N, 117°12'W), 19 Apr 1993, R. S. Zack (4♂, 3♀; WSU); Steptoe Canyon (16 km SW Colton; 46°27.1'N, 117°12.4'W), 27 Apr-3 Aug 1974, 1985, W. J. Turner, R. S. Zack (15♂, 8♀; WSU); Wawawai to Pullman (46°40.4'N, 117°16.3'W), 2 Apr 1950, R. Spurrier (1♂, 3♀; WSU); Wawawai (46°38'N, 117°22.8'W), 22 Apr 1956, V. Newhouse (1♀; USNM); Yakawawa Canyon (43°34.6'N, 117°17.3'W), 2 May 1978, R. S. Zack (4♂, 3♀; WSU). *Yakima*: Chinook Pass (16 km E; American River; 46°52.3'N, 121°29'W; 1035 m), 24 Jul 1976, W. J. Turner (1♀; WSU); Tieton (13 km SW ranger station; Bear Creek; Snoqualmie National Forest; 46°42.1'N, 120°45.3'W), 11-12 Jun 1973, W. J. Turner (1♂, 1♀; WSU).

*WYOMING. Carbon*: Lake Marie (20 km W Centennial; 41°20'N, 106°19.5'W; 3230 m), 1 Aug 1973, P. H. Arnaud, Jr. (1♀; CAS). *Crook*: Alva (near; 44°38.6'N, 104°22.8'W; 1330 m), 20 Jun 2008, D. and W. N. Mathis (6♂, 3♀; USNM). *Fremont*: Lander (42°50'N, 108°43.8'W), 16 Aug 1950, A. H. Sturtevant (7♂, 4♀; USNM). *Niobrara*: Lusk (42°45.7'N, 104°27.1'W), Jul 1895 (1♂; ANSP). *Teton*: Yellowstone National Park, Yellowstone Lake (44°23.8'N, 110°22'W), 9 Aug 1918, A. L. Melander (1♂, 1♀; ANSP).

**Figures 16.**
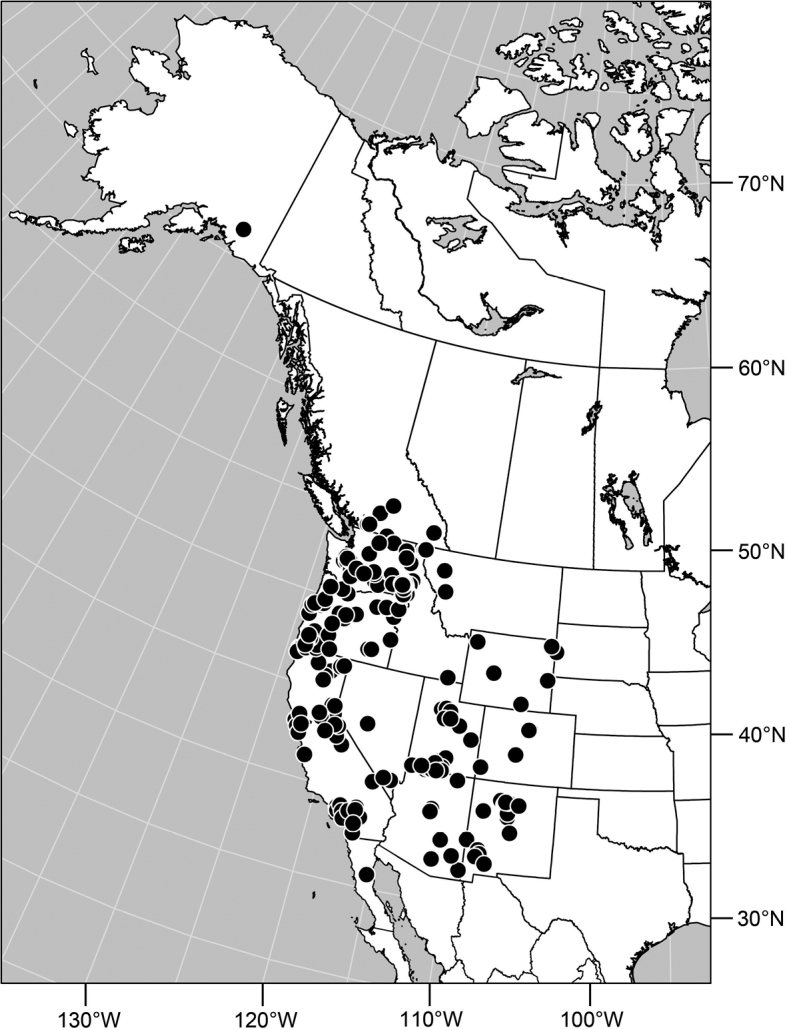
Distribution map of *Gymnoclasiopa argyrostoma* (Cresson).

**Distribution** (Fig. 16). Nearctic: Canada (British Columbia), Mexico (Baja California), United States (Alaska, Arizona, California, Colorado, Idaho, Montana, Nevada, New Mexico, Nevada, Oregon, South Dakota, Utah, Washington, Wyoming).

**Remarks.** Among Nearctic species, this is by far the most abundant and commonly collected, and is found throughout the West (Fig. 16). This is also one of the more easily recognized species, making it unlikely to be confused with congeners. It is distinguished by the entirely black antennae and forelegs and the subshiny to shiny mesonotum and abdomen.

### Gymnoclasiopa bella

(Mathis) comb. n.

http://species-id.net/wiki/Gymnoclasiopa_bella

Figs 17 –20

Ditrichophora bella Mathis 1997: 700.

#### Diagnosis.

**Figures 17–19.**
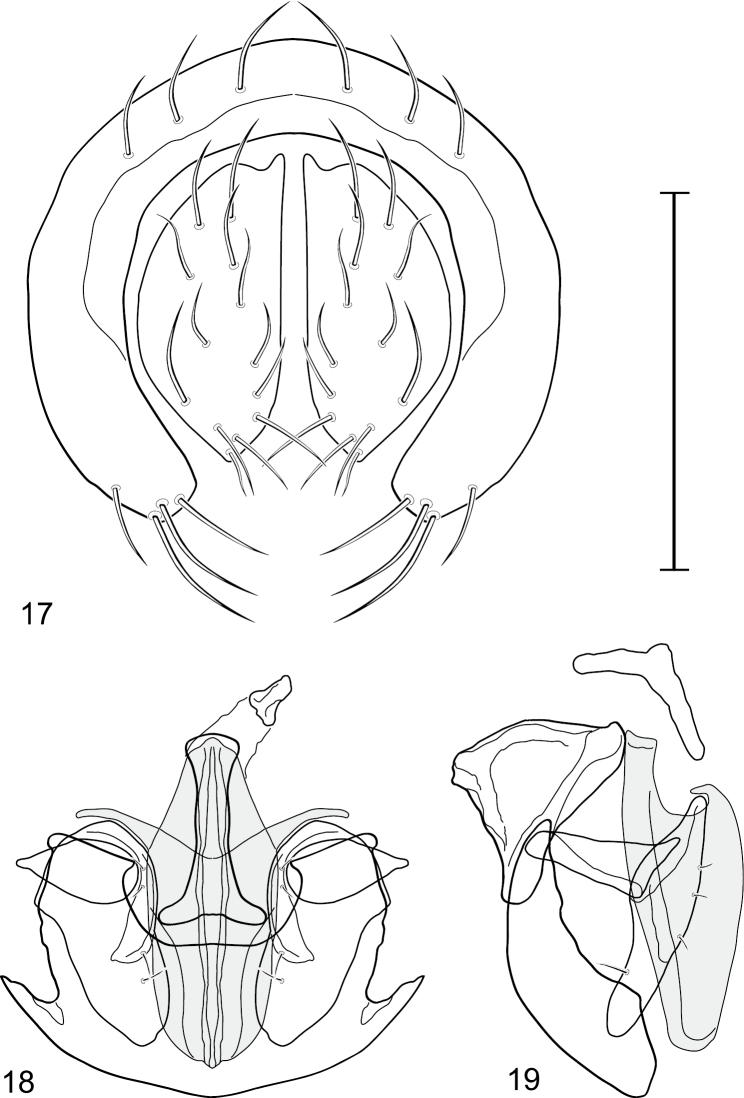
*Gymnoclasiopa bella* (Mathis) (Dominican Republic. Monseñor Nouel: near Jima). **17** epandrium and cerci, posterior view **18** internal structures of male terminalia (aedeagus [shaded], phallapodeme, gonite, hypandrium), ventral view **19** same, lateral view. Scale bar = 0.1 mm.

This species is distinguished from congeners by the following combination of characters: Small to medium-sized shore flies, body length 1.90–3.10 mm; generally shiny black. *Head*: Frons of male generally moderately microtomentose to anterior margin, only small, linear patch anterolaterally and at base of setae bare of microtomentum; frons of female with small bare areas anteriorly; 1 proclinate, fronto-orbital seta, inserted just behind and laterad of reclinate seta. Antenna black; apical margin of pedicel and 1st flagellomere invested with whitish gray microtomentum; arista bearing 5 dorsal rays. Face bearing 2 lateral, facial setae, dorsal seta inserted at level of facial prominence, ventral seta inserted toward ventral margin, distance between facial setae about equal to length of basal flagellomere; face black but mostly invested with silvery white microtomentum, only ventral portion of antennal grooves, vertical stripe immediately adjacent to parafacial, and medial stripe that curves laterally ventrally bare, shiny, black; parafacial completely microtomentose, whitish gray; gena moderately short; gena-to-eye ratio 0.10–0.13. Mouthparts, including maxillary palpus, black. *Thorax*: Mesonotum generally sparsely microtomentose, golden brown, becoming sparser to bare laterally, through supra-alar area, thereafter laterally sparsely microtomentose, male bearing a very distinctive stripe of dense, fine, brown microtomentum extended from postpronotum through most of notopleuron, female shiny black, similar to adjacent areas; prescutellar acrostichal setae weakly developed. Wing hyaline; costal ratio 0.37–0.44; M vein ratio 0.60–0.63; halter stem blackish brown; knob yellowish to whitish yellow. Legs except tarsi black, mostly shiny, femora with some surfaces very sparsely microtomentose; tarsi yellow except apical 2 brown; forefemur unadorned with short, peglike setulae along posteroventral surface. Halter white. *Abdomen*: Black, generally shiny, especially laterally and ventrally. Male terminalia (Figs 17–19): Epandrium in posterior view (Fig. 17) as a broadly rounded, inverted U, dorsal portion slightly narrower than lateral arms, in lateral view wider ventrally; cercus semilunate in posterior view (Fig. 17); aedeagus in lateral view (Fig. 19) slipper-like, base deeply and unevenly incised, tapered toward apex, apex moderately narrowly rounded, in ventral view (Fig. 18) narrowly thumb-like with basal third tapered, basal margin narrowly truncate, apical 2/3 nearly parallel-sided, apex moderately widely rounded, with thin, wing-like, narrow projections sub-basally; phallapodeme in lateral view (Fig. 19) more or less triangular, in ventral view (Fig. 18) as an inverted T; ejaculatory apodeme in lateral view (Fig. 19) L-shaped; postgonite in lateral view (Fig. 19) acutely narrow basally, thereafter becoming wider than apex narrowed, digitiform, bearing 2-3 setulae along basoposterior margin and 1 setula along margin toward hypandrium; pregonite in lateral view (Fig. 19) moderately elongate, tapered, narrowed toward hypandrium, expanded toward aedeagus, aedeagal end truncate; hypandrium in ventral view (Fig. 18) wide, width nearly twice length, broadly and shallowly rounded along anterior margin with anterolateral, pointed, lateral projections, deeply incised along posterior margin medially, in lateral view (Fig. 19) arched, posterior angle acute, thereafter anteriorly becoming wider, widest before anterior margin.

#### Type material.

The holotype male of *Ditrichophora bella* Mathis is labeled “**DOMINICAN RP.** Monsñ. Nouel: nr. Jima, 670m, 19°01.2'N, 70°28.8'W[,] 10May1995, W.N.Mathis/HOLOTYPE Ditrichophora bella ♂ W.N.Mathis USNM [red; species name and gender handwritten].” The holotype is double mounted (minuten pin in block of plastic), is in excellent condition, and is in the USNM. The allotype and 24 paratypes (14♂, 10♀; USNM) bear the same locality label as the holotype. Other paratypes are as follows: *JAMAICA. St. Andrew*: Hardwar Gap (18°04.2'N, 76°44'W), 17 May 1996, D. and W. N. Mathis, H. B. Williams (1♂, 1♀; USNM). *MEXICO. Chiapas*: El Triunfo (49 km S Jaltenango; 1800 m), 14 May 1985, W. N. Mathis (1♂; USNM).

#### Type locality.

Dominican Republic. Monseñor Nouel: near Jima (19°01.2'N, 70°28.8'W; 670 m).

#### Distribution

**Figure 20.**
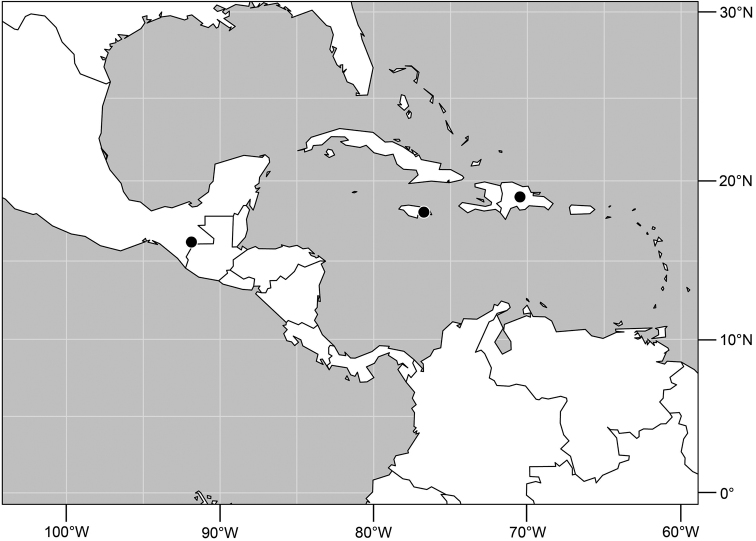
Distribution map of *Gymnoclasiopa bella* (Mathis).

(Fig. 20). *Neotropical*: Mexico (Chiapas), West Indies (Dominican Republic, Jamaica).

#### Remarks.

All specimens were collected in montane habitats that are frequently overcast if not enshrouded in a foggy mist. The specimens from the Dominican Republic were mostly collected from a pile of spoiling cabbage that had been discarded along the roadside.

This species is sexually dimorphic with males having a brown stripe of dense but fine microtomentum extended from the postpronotum to the posterior margin of the notopleuron. Females are shiny black throughout this area of the thorax, similar to portions of the mesonotum that are immediately adjacent.

Based on external characters, this species was placed initially in the genus *Ditrichophora*, but structures of the male terminalia indicate a closer association with *Gymnoclasiopa*.

This species is distinguished from congeners, especially *Gymnoclasiopa chiapas*, by the following combination of characters: Postpronotum and most of notopleuron of male densely invested with fine, brown microtomentum, contrasted sharply with generally shiny, adjacent mesonotum and anepisternum; prescutellar acrostichal setae weakly developed; frons of male generally sparsely microtomentose to anterior margin; halter white; only 1 proclinate fronto-orbital seta; and pattern of silvery white microtomentum on face (see species description).

### Gymnoclasiopa bohemanni

(Becker)

http://species-id.net/wiki/Gymnoclasiopa_bohemanni

Figs 21 –24

Clasiopa bohemanni Becker 1896: 159.
Discocerina bohemanni . Cresson 1925: 256 [generic combination].
Ditrichophora bohemanni . Hackman 1980: 128 [generic combination].
Gymnoclasiopa bohemanni . Mathis and Zatwarnicki 1995: 175 [generic combination; world catalog].
Ditrichophora (Gymnoclasiopa) montana Cresson 1942: 120. Wirth 1965: 739 [Nearctic catalog]. Cole 1969: 398 [fauna, western North America]. NEW SYNONYM
Gymnoclasiopa montana . Mathis and Zatwarnicki 1995: 177 [generic combination; world catalog].

#### Diagnosis.

**Figures 21–23.**
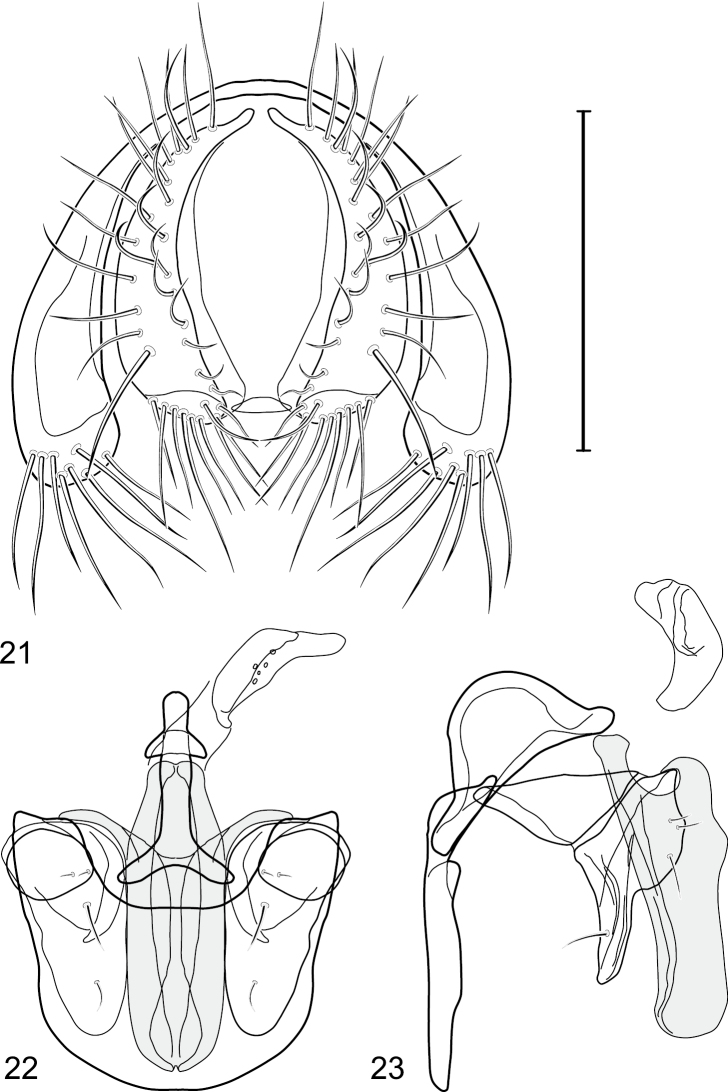
*Gymnoclasiopa bohemanni* (Becker) (Canada. Alberta. Okotoks, Sheep River). **21** epandrium and cerci, posterior view **22** internal structures of male terminalia (aedeagus [shaded], phallapodeme, gonite, hypandrium), ventral view **23** same, lateral view. Scale bar = 0.1 mm.

This species is distinguished from congeners by the following combination of characters: Small to medium-sized shore flies, body length 1.80–3.35 mm; head and thorax generally microtomentose gray, abdomen subshiny to shiny black. *Head*: Frons densely but finely microtomentose. Antenna yellowish, sometimes basal flagellomere slightly darkened dorsally; arista bearing 5 dorsal rays. Face relatively flat with antennal grooves inconspicuous; facial microtomentum generally sericeous, bright yellow (males) or grayish yellow (females, grayer in antennal grooves); gena moderately high, gena-to-eye ratio 0.14–0.18. Maxillary palpus yellow to yellowish red. *Thorax*: Anterolateral area of mesonotum, just mediad of area from postpronotum through notopleuron, densely microtomentose, mostly dull; lateral mesonotal area from and including postpronotum and notopleuron, densely and finely microtomentose; this area of males brown, contrasted with whitish to silvery gray microtomentum of broad, medial portion; same area in female concolorous with medial coloration. Wing of hyaline, not darkened along anterior region; costal ratio 0.0.39–0.50; M vein ratio 0.50–0.59; halter stem yellowish tan, knob yellowish white to white. Forecoxa yellow ventrally; foretibia mostly yellow, sometimes slightly brownish to grayish medially; midtibia mostly yellow to mostly grayish with only apices yellow, but with brownish area around apical 1/3; hindtibia without ventroapical spur, mostly grayish except for apices. *Abdomen*: Tergites 1-4 sparsely and finely microtomentose medially, subshiny; lateral margins of tergites and tergite 5 shiny black with microtomentum either lacking or sparse. Male terminalia (Figs 21–23): Epandrium in posterior view (Fig. 21) as a broadly formed, inverted U with the base more narrowly formed, dorsal portion more thinly developed than lateral arms, lateral arms widespread ventrally, shallowly arched, enlarged ventrally, broadly rounded, setulae more clustered at ventral margin; cercus in posterior view (Fig. 21) elongate, irregularly lunate, dorsal apex very narrow, digitiform, expanded toward ventral apex, ventral apex rounded, both lateral and medial margins arched, setulae more clustered at ventral margin; aedeagus in lateral view (Fig. 23) slipper-like, base shallowly emarginate, tapered very gradually toward apex, apical half nearly parallel sided, apex bluntly rounded, in ventral view (Fig. 22) elongate, expanded laterally from narrow base on basal 1/4, thereafter to apex almost parallel sided, apical margin rounded with tiny notch medially; phallapodeme in lateral view (Fig. 23) more or less irregularly triangular, extension toward hypandrium more elongate than angle towards aedeagal base, in ventral view (Fig. 22) I-shaped, subapical crossbar shorter and basal crossbar, with tapered shoulders, basal crossbar wider, widely Y-shaped, apical margin very shallowly emarginate; ejaculatory apodeme in lateral view robustly comma-shaped, in ventral view obtusely L-shaped; postgonite in lateral view (Fig. 23) with basal half robust, thereafter abruptly tapered to a narrow, slightly tapered, digitiform process, posterior margin with a few setulae, extended process with a single, longer setula, in ventral view (Fig. 22) as an elongate isosceles triangle, tapered gradually toward apex, width of base about half length, angles rounded, lateral and medial margins nearly straight; pregonite in lateral view (Fig. 23) moderately elongate, triangular, width at base almost twice lengths of sides, in ventral view (Fig. 22) lunate with apices pointed; hypandrium in ventral view (Fig. 22) robustly V to U-shaped, lateral margins slightly expanded posteriorly, anterior margin very broadly rounded, posterior margin conspicuously emarginate, widely U-shaped, in lateral view (Fig. 23) narrowly elongate, nearly straight.

#### Type material.

Lectotype female of *Clasiopa bohemanni* Becker is labeled “[empty red square]/Type [printed]/Bohemanni Beck [handwritten]/39 [printed]/174 57 [beige; “57” handwritten]/22 64 [beige; “64” handwritten]/LECTOTYPE ♀ *Clasiopa bohemanni* Becker by Mathis & Zatwarnicki NRS [red].” The lectotype female is double mounted, is in good condition (anterior margin of right wing slightly broken near middle), and is deposited in the NRS. When Becker (1896; 159) described this species he noted that “diese neue Art fand ich in Bohemann’s Sammlung.”

The holotype male of *Ditrichophora montana* Cresson is labeled “GLACIER PARK Avalanche L[a]k[e] [48°39.4'N, 113°47.1'W] 14 July 1935[,] A. L. Melander/TYPE Ditrichophora Montana Cress HoloTYPE 6628 [red; all except “TYPE” handwritten].” The holotype is double mounted (minuten pin in a rectangular card), is in excellent condition, and is deposited in the ANSP (6628).

**Type locality.** Not given, “Bohemann’s Sammlung” (= ? Sweden).

#### Other specimens examined.

Nearctic. CANADA. *ALBERTA*. Okotoks, Sheep River Campground (50°43.4'N, 113°58.3'W), 27 Jun 1968, W. W. Wirth (1♂; USNM).

*BRITISH COLUMBIA*. Emerald Lake, Yoho National Park (51°26.3'N, 116°32.5'W); 30 Jul 1935, A. L. Melander (1♀; ANSP). Martin Creek, Alaska Highway DC 243 (57°17.3'N, 121°28'W), 13 Aug 1978, P. H. Arnaud, Jr. (1♀; CAS). Pine Pass (37 km NE; highway 97; 55°30'N, 122°40'W), 25 Jun 1978, P. H. Arnaud, Jr. (3♂, 1♀; CAS). Prophet River Provincial Campground, Alaska Highway DC 221 (57°58'N, 122°47'W), 13 Aug 1978, P. H. Arnaud, Jr. (1♀; CAS). Terrace (52 km SW; 54°14.8'N, 130°17.2'W), 5 Jul 1960, J. G. Chillcott (1♂; USNM).

*MANITOBA*. The Pas (53°59.5'N, 101°15.2'W), 31 Jul 1937, D. G. Denning (1♀; USNM).

*NEWFOUNDLAND*. Cachrane Pond (47°58'N, 57°13.4'W), 30 Jun 1961, C. P. Alexander (1♀; USNM). Terra Nova National Park (48°31.8'N, 53°55.7'W), 6-8 Jul 1961, C. P. Alexander (3♂; USNM).

*ONTARIO*. Klotz Lake (49°48.1'N, 85°51.8'W), 5 Jul 1954, A. H. Sturtevant (1♂, 1♀; USNM).

*SASKATCHEWAN*. Saskatoon, Beaver Creek (52°08.4'N, 106°41.2'W), 28 Aug 1955, W. W. Wirth (1♂; USNM).

*YUKON TERRITORY*. Aishihik River, Alaska Highway DC 996.8 (61°40.3'N, 137°28.3'W), 7 Aug 1978, P. H. Arnaud, Jr. (2♂, 1♀; CAS).

UNITED STATES. *ALASKA. Anchorage*: Anchorage (61°13.1'N, 149°54'W; Seward Highway), 5 Aug 1964, K. M. Sommerman (3♂; USNM); Mirror Lake (61°25.7'N, 149°24.9'W), 5 Aug 2002, D. and W. N. Mathis (1♀; USNM). *Fairbanks-Northstar*: Colorado Creek, Chena Hot Springs (65°03.2'N, 146°02.9'W), 11 Jul 1978, P. H. Arnaud, Jr. (1♂, 1♀; CAS). *Juneau*: Douglas (58°16.5'N, 134°23.6'W), 19 Jun 1954, R. Coleman (1♀; USNM); Gastineau Channel, Thane Road (S Juneau; 58°16.9'N, 134°22.4'W), 20-22 Jul 2011, D. and W. N. Mathis (31♂, 9♀; USNM); Juneau, Mendenhall Valley, Riverside Rotary Park (58°22.8'N, 134°35.2'W), 21 Jul 2011, D. and W. N. Mathis (1♂; USNM). *Kenai Peninsula*: Homer (59°38.8'N, 151°31.5'W), 2 Aug 2002, D. and W. N. Mathis (2♂, 1♀; USNM); Kenai Fjord National Park, Exit Glacier (60°11.7'N, 149°35.8'W; wetlands), 30 Jul 2002, D. and W. N. Mathis (1♀; USNM); Kenai River, Jim’s Landing (60°28.9'N, 150°06.9'W), 3 Aug 2002, D. and W. N. Mathis (6♂, 1♀; USNM); Ninilchik (60°03'N, 151°40.2'W; beach), 2 Jul 2006, D. and W. N. Mathis (2♂, 1♀; USNM); Seward (60°08.3'N, 149°23.2'W), 7 Aug 2003, D. and W. N. Mathis (1♀; USNM); Seward (21 km N; 60°17.2'N, 149°20.5'W; Snow River), 31 Jul 2002, D. and W. N. Mathis (28♂, 12♀; USNM); Skilak Lake (60°26.3'N, 150°19.4'W), 3 Aug 2002, D. and W. N. Mathis (4♂, ♀; USNM). *Matanuska-Susitna*: Eklutna (Knik Arm; 61°28.2'N, 149°21.4'W), 7 Aug 2002, D. and W. N. Mathis (2♂, 2♀; USNM); Hatcher Pass (61°45'N, 149°13.9'W), 6 Aug 2002, D. and W. N. Mathis (1♂; USNM); Knik River (61°27.8'N, 148°51.6'W), 5 Aug 2002, D. and W. N. Mathis (33♂, 7♀; USNM); Little Willow Creek (61°48.6'N, 150°05.8'W; 50 m), 25 Jul 2011, D. and W. N. Mathis (10♂; USNM); Matanuska (61°32.5'N, 149°13.8'W; rotary trap), 28 Apr-21 May 1945, J. C. Chamberlin (1♂, 1♀; USNM); Palmer (61°36'N, 149°06.8'W; jeep trap), 7-13 Jul 1964, K. M. Sommerman (2♂, 6♀; USNM); Palmer (Knik River; 61°31.2'N, 148°59.4'W), 6 Aug 2002, D. and W. N. Mathis (1♀; USNM); Palmer (Matanuska River; 61°36.5'N, 149°04.1'W), 5-16 Aug 2002, 2012, D. and W. N. Mathis (8♂, 4♀; USNM); Sheep Creek (61°58.3'N, 150°05'W; 55 m), 10 Aug 2011, D. and W.N. Mathis (2♂, 6♀; USNM); Talkeetna (Susitna River; 61°19.4'N, 150°07.2'W; 120 m), 10 Aug 2011, D. and W.N. Mathis (8♂, 2♀; USNM); Willow Creek (61°46.1'N, 150°04.2'W; 50 m), 26 Jul 2011, D. and W.N. Mathis (1♂; USNM). *Southeast Fairbanks Census Area*: Dry Creek Campground, Glenn Highway A-192 (63°39.2'N, 144°21.8'W), 3 Aug 1978, P. H. Arnaud, Jr. (1♀; CAS); Gardiner Creek Camp, Alaska Highway DC 1253 (62°51.5'N, 141°28'W), 5 Aug 1978, P. H. Arnaud, Jr. (1♀; CAS); Gerstle River, Alaska Highway DC 1393 (64°03.4'N, 145°08.1'W), 9 Jul 1978, P. H. Arnaud, Jr. (2♂; CAS). *Valdez-Cordova (Census Area)*: Glennallen (32 km W; 62°05.9'N, 146°05.4'W; 665 m), 27 Jul 2011, D. and W. N. Mathis (8♂, 2♀; USNM); Gulkana River (19.3 km N Glenallen; 62°16.1'N, 145°23.1'W), 27 Jul-7 Aug 2011, 2012, D. and W. N. Mathis (17♂, 6♀; USNM); Klutina River (mile 101; 61°57.2'N, 145°19.3'W; 315 m), 7 Aug 2012, D. and W. N. Mathis (1♂; USNM); Tolsona Creek State Campground, Glenn Highway, A 173 (62°03.9'N, 145°59.8'W), 31 Jul 1978, P. H. Arnaud, Jr. (1♂; CAS); Valdez (61°07.5'N, 146°21.5'W; Ruth Pond), 8 Jul 2006, D. and W. N. Mathis (2♂, 1♀; USNM); Valdez, Valdez Glacier Campground (61°07'N, 146°12.6'W), 1 Aug 1978, P. H. Arnaud, Jr. (1♂, 1♀; CAS).

*COLORADO. Chaffee*: Green Timber Gulch, Cottonwood Lake (6 km W; 38°45.6'N, 106°20.3'W), 10-12 Jul 1978, T. W. and W. T. Davies (1♂; CAS); Monarch Pass (38°29.8'N, 106°19.5'W; 2440 m), 21 Jun 1940, A. L. Melander (2♂; USNM). *Gunnison*: Schofield Pass on Gothic Road (39°0.9'N, 107°02.8'W; 2900 m), 1 Aug 1957, A. H. Sturtevant (5♀; USNM). *Rio Grande*: South Fork (37°40.2'N, 106°38.4'W; 2440 m), 20 Jun 1972, W. W. Wirth (1♂, 1♀; USNM). *La Plata*: Durango (37°16.5'N, 107°52.8'W), 10 Aug 1950, A. H. Sturtevant (5♂; USNM). *Larimer*: Estes Park (40°22.6'N, 105°31.3'W), 13 Jul 1934, A. L. Melander (1♀; ANSP); Hidden Valley, Rocky Mountain National Park (40°23.6'N, 105°38.8'W), 8 Aug 1934, A. L. Melander (1♂; ANSP); Virginia Dale (40°57.3'N, 105°21'W), 27 Jul 1953, R. R. Dreisbach (1♀; USNM).

*IDAHO. Latah*: Big Meadow Creek Recreation Area (11.25 km N Troy; 46°51'N, 116°44.7'W; 915 m; sweeping), 7 Aug 1986, W. J. Turner (1♂; WSU).

*MONTANA. Flathead*: Glacier National Park, Lake McDonald (48°35'N, 113°55.6'W), 13 Jun 1935, A. L. Melander (1♀; ANSP).

*NEBRASKA. Dawes*: Chadron (42°42.5'N, 103°01'W), 20 Aug 1950, A. H. Sturtevant (1♂; USNM).

*SOUTH DAKOTA. Custer*: Sylvan Lakes (42°50.6'N, 103°33.7'W), 19 Jul 1924 (1♀; ANSP).

*UTAH. Carbon*: Deadman Canyon (16 km NE Price; 39°41.7'N, 110°44'W; 2055 m), 14 Aug 2008, D. and W. N. Mathis (5♂, 4♀; USNM). *Garfield*: Grand Staircase-Escalante National Monument: spring off highway 12, Henrieville (12 km E; 37°36.8'N, 111°53.8'W; 2005 m), 2–17 Aug 2000, W. N. Mendel, E. C. Green, M. Moody (1♂; BYU). *Grand*: Harley Dome (39°10.4'N, 109°08'W), 13 Aug 1958 (1♀; USNM). *Salt Lake*: Little Cottonwood Canyon (40°34.7'N, 111°47.8'W), 23 Aug 1940, A. L. Melander (1♀; USNM). *Sanpete*: Ephraim (39°21.6'N, 111°35.2'W), 18 Aug 1953, A. H. Sturtevant (1♀; USNM).

*WASHINGTON. Island*: Keystone Ferry (1.6 km E; 48°10.1'N, 122°38.2'W), 13 Aug 1977, R. S. & V. L. Zack (1♂; WSU). *Pierce*: Mount Rainier National Park, Summerland Trail (46°52.0'N, 121°38.1'W), 24 Jul 1924, A. L. Melander (1♀; ANSP). *Whitman*: Steptoe Canyon (16 km SW Colton; 46°27.1'N, 117°12.4'W; Malaise trap with dry ice), 3 Aug 1974, W. J. Turner (1♂; WSU).

*WYOMING. Sublette*: Slide Lake (43°16.5'N, 109°46.9'W), 14 Aug 1951, A. H. Sturtevant (3♂, 2♀; USNM). *Teton*: Teton Pass (43°29.9'N, 110°57.3'W), 10 Aug 1951, A. H. Sturtevant (2♂; USNM); Yellowstone National Park, Riverside (44°28.4'N, 110°50.4'W), 4 Aug 1919, A. L. Melander (1♀; ANSP).

#### Distribution

**Figure 24.**
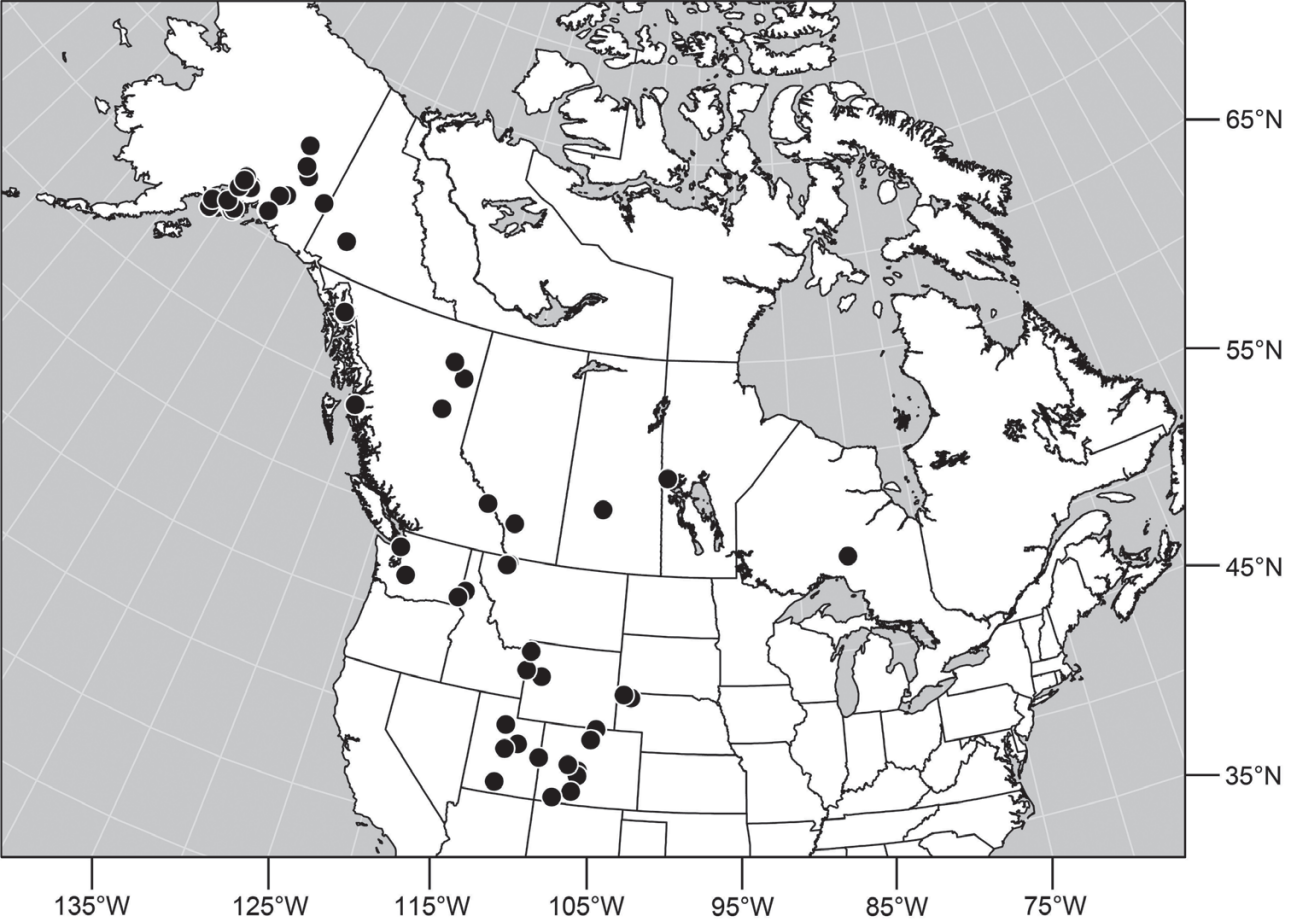
Distribution map of *Gymnoclasiopa bohemanni* (Becker).

(Fig. 24). *Nearctic*: Canada (Alberta, British Columbia, Manitoba, Newfoundland, Ontario, Quebec, Saskatchewan, Yukon Territory), United States (Alaska, Colorado, Idaho, Montana, Nebraska, South Dakota, Utah, Washington, Wyoming). *Palearctic*: Austria, Finland, Iceland, Mongolia (Bayan Ölgiy, Khovd), Sweden.

#### Remarks.

This species, like many but not all congeners, is sexually dimorphic, especially the coloration of the face and the mesonotal area from the postpronotum to the base of the wing as noted in the diagnosis. These differences, which were not always recognized, probably was a major factor contributing to this species being described more than once.

This species is similar to *Gymnoclasiopa pulchella* in having yellowish antennae, maxillary palpi, and foretibiae (sometimes brownish apically) but is distinguished from that species as follows (also see key): The anterolateral area of the mesonotum, just mediad of the area from the postpronotum through the notopleuron is densely microtomentose, mostly dull; the lateral mesonotal area from and including the postpronotum and the notopleuron is densely and finely microtomentose (this area in males is brown and is contrasted with whitish to silvery gray microtomentum of the broad, medial portion; the same area in females is concolorous with the medial coloration).

### Gymnoclasiopa chiapas

(Mathis) comb. n.

http://species-id.net/wiki/Gymnoclasiopa_chiapas

Figs 25 –28

Ditrichophora chiapas Mathis 1997: 702.

#### Diagnosis.

**Figures 25–27.**
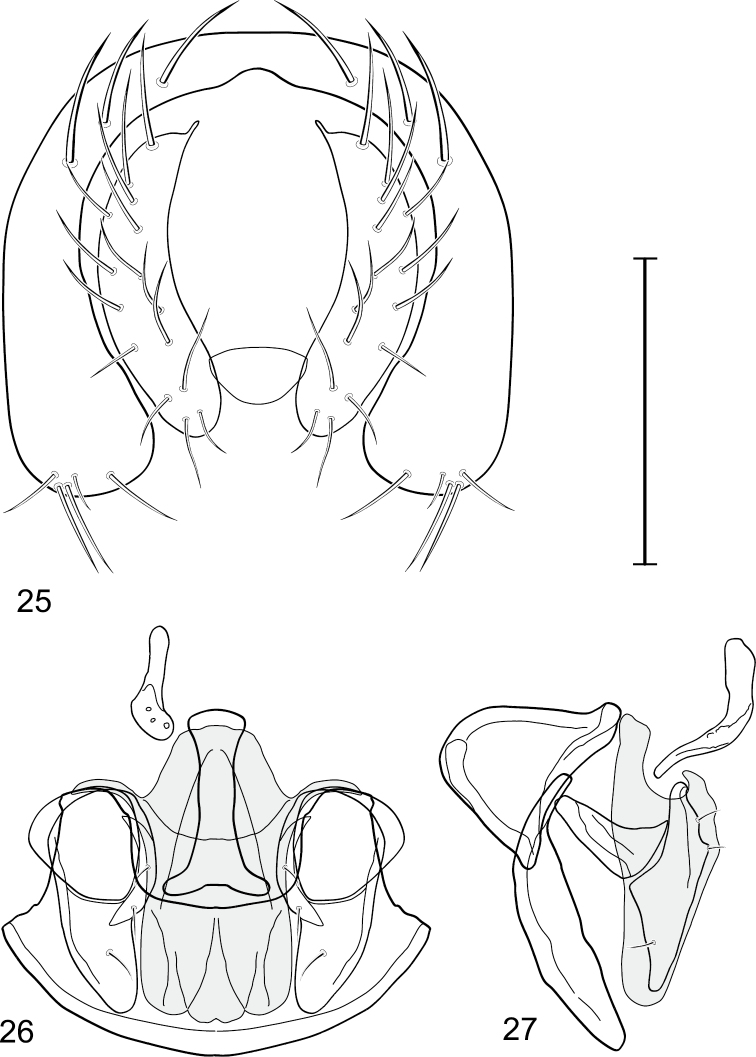
*Gymnoclasiopa chiapas* (Mathis) (Mexico. Chiapas. Biosfera El Triunfo). **25** epandrium and cerci, posterior view **26** internal structures of male terminalia (aedeagus [shaded], phallapodeme, gonite, hypandrium), ventral view **27** same, lateral view. Scale bar = 0.1 mm.

This species is distinguished from congeners by the following combination of characters: Moderately small shore flies, body length 2.00–2.65 mm; generally shiny black. *Head*: Frons of male and female similar, moderately microtomentose on posterior portion, although bare at bases of setulae and small area just laterad of posterior ocelli, anterior third of frons bare, shiny, with 2 lunate indentations, medial area with pointed extension of moderately microtomentum from posterior portion; 1 proclinate, fronto-orbital seta, inserted just behind and laterad of reclinate seta. Antenna black; apical margin of pedicel and basal flagellomere invested with whitish gray microtomentum; arista bearing 5 dorsal rays. Face bearing 3 lateral, facial setae, dorsal seta longest, inserted at level of facial prominence, ventral setae progressively shorter, evenly spaced with distance between less than width of 1st flagellomere; face mostly black, shiny, but with pattern of silvery white microtomentose, vertical stripes; lateral facial stripe immediately adjacent to parafacial, other vertical stripe just laterad of midfacial vertical bare area; also silvery white microtomentose on dorsal portion of antennal grooves and along ventral, facial margin; parafacial bare, shiny black; gena-to-eye ratio 0.10–0.12. Mouthparts, including maxillary palpus, black. *Thorax*: Mesonotum generally sparsely microtomentose, golden brown, becoming bare laterally through supra-alar area and continuing ventral through pleural area; prescutellar acrostichal setae well developed. Wing hyaline; costal ratio 0.40–0.43; M vein ratio 0.70–0.73; halter stem blackish brown; knob yellowish to whitish. Legs except tarsi black, mostly shiny, femora with some surfaces very sparsely microtomentose; tarsi yellow except apical 1-2 brown; forefemur with row of numerous, very short, peglike setulae along posteroventral surface. *Abdomen*: Tergites black, generally shiny, especially laterally and ventrally. Male terminalia (Figs 25–27): Epandrium in posterior view (Fig. 25) moderately broadly as an inverted, robust U, dorsal portion nearly straight, narrower than lateral arms, ventral margins of arms slightly expanded, broadly rounded, in lateral view wider ventrally; cercus in posterior view (Fig. 25) semilunate, elongate, narrow, shallowly arched, parallel sided; aedeagus in lateral view (Fig. 27) slipper-like, base deeply and unevenly incised, tapered toward apex, apex moderately narrowly rounded, in ventral view (Fig. 26) broadly tapered on basal third, basal margin almost truncate, very shallowly arched, with thin, wing-like, narrow projections sub-basally, apical margin twice as broad as basal margin, shallowly crenulate; phallapodeme in lateral view (Fig. 27) more or less triangular, in ventral view (Fig. 26) as an inverted T; ejaculatory apodeme in lateral view (Fig. 27) obtusely L-shaped, tapered from wider apex to narrow base; postgonite in lateral view (Fig. 27) as a parallelogram, acutely narrow basally and apically, each lateral margin obtusely angulate, bearing 2 setulae along basoposterior margin and 1 setula along margin toward hypandrium; pregonite in lateral view (Fig. 27) moderately elongate, tapered, narrowed toward hypandrium, flared toward aedeagus, aedeagal end truncate, in ventral view shallowly arched medially, thereafter laterally slightly tapered, lateral margin rounded; hypandrium in ventral view (Fig. 26) broadly and shallowly rounded along anterior margin, in lateral view (Fig. 27) arched, posterior portion digitiform, thereafter anteriorly becoming wider, dish-like.

#### Type material.

The holotype male of *Ditrichophora chiapas* Mathis is labeled “MEXICO. Chiapas: El Triunfo (49 km S Jaltenango) 14 May 1985, 1800 m[,] Wayne N. Mathis/HOLOTYPE Ditrichophora chiapas W.N.Mathis USNM [red; species name handwritten].” The holotype is double mounted (minuten pin in block of plastic), is in excellent condition, and is in the USNM. The allotype female and four paratypes (4♂; USNM) bear the same locality label as the holotype. Other paratypes are as follows: MEXICO. Chiapas: El Triunfo (49 km S Jaltenango; 1300-2000 m), 13-15 May 1985, W. N. Mathis (3♂; UNAM, USNM).

#### Type locality.

Mexico. Chiapas. Biosfera El Triunfo (ca 49 km S Jaltenango; 15°39.5'N, 92°48.5'W; 1800 m).

#### Distribution

**Figure 28.**
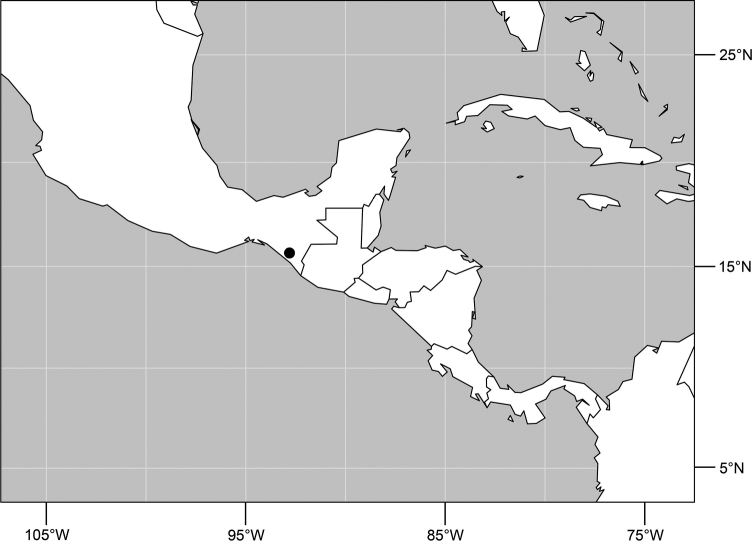
Distribution map of *Gymnoclasiopa chiapas* (Mathis).

(Fig. 28). *Neotropical*: Mexico (Chiapas).

#### Remarks.

El Triunfo is a site in the cloud forest of southern Mexico (some of the only cloud forest that remains largely undisturbed in Mexico).

Based on external characters, this species was placed initially in the genus *Ditrichophora*, but structures of the male terminalia indicate a closer association with *Gymnoclasiopa*.

This species is distinguished from congeners, especially *Ditrichophora bella*, by the following combination of characters: Postpronotum and notopleuron of males are generally bare of microtomentum and shiny, similar to the mesonotum and anepisternum; the prescutellar acrostichal setae are well developed; males have the anterior third of the frons bare of microtomentum, shiny black; halteres are white; there is only one proclinate fronto-orbital seta; and by the pattern of silvery white microtomentum on face (see species description).

### Gymnoclasiopa grecorum

Mathis & Zatwarnicki sp. n.

urn:lsid:zoobank.org:act:29AD8BF1-45F7-41C9-A756-6DD850D7C623

http://species-id.net/wiki/Gymnoclasiopa_grecorum

**Figures 29–30.**
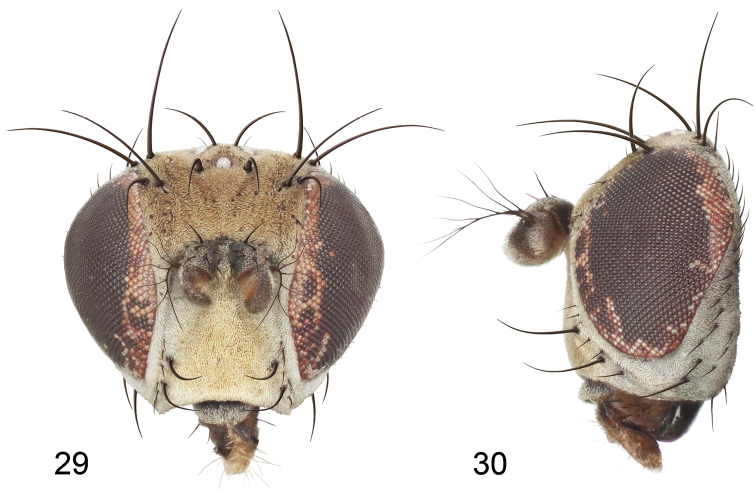
*Gymnoclasiopa grecorum* Mathis and Zatwarnicki, new species (USA. Alaska. Juneau: Thane Road). **29** head, anterior view **30** same, lateral view.

Figs 29 [Fig F11] –34

#### Diagnosis.

**Figures 31–33. F11:**
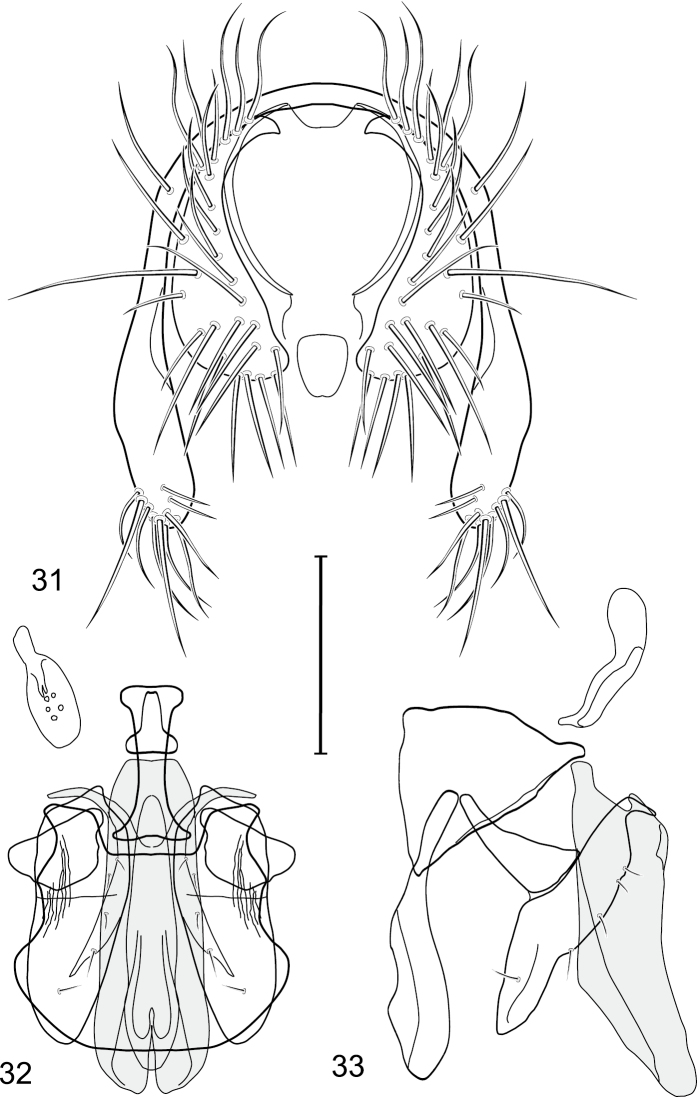
*Gymnoclasiopa grecorum* Mathis and Zatwarnicki, new species (USA. Alaska. Juneau: Thane Road). **31** epandrium and cerci, posterior view **32** internal structures of male terminalia (aedeagus [shaded], phallapodeme, gonite, hypandrium), ventral view **33** same, lateral view. Scale bar = 0.1 mm.

This species is distinguished from congeners by the following combination of characters: Moderately small to medium-sized shore flies, body length 2.10–3.15 mm; head generally dull, thorax faintly subshiny medially, dull, microtomentose anteriorly and laterally, microtomentose gray, abdomen black with thin investment of whitish microtomentum. *Head* (Figs 29–30): Frons densely microtomentose, dull, gray to blackish gray. Antenna black; basal flagellomere covered with silvery gray microtomentum, with faint reddish orange coloration mediobasally; arista bearing 5-6 dorsal rays. Face somewhat flat, antennal grooves generally inconspicuous, oriented ventrolaterally to vertical; facial microtomentum generally yellow to golden yellow, slightly grayish to silvery white in antennal grooves; parafacial and gena whitish gray to white; genal height about equal to height of basal flagellomere; gena moderately short; gena-to-eye ratio 0.17–0.20. Maxillary palpus varying from mostly black but with some reddish to yellowish coloration at middle to entirely yellowish. *Thorax*: Mesonotum and pleural area generally densely microtomentose, gray to tannish gray, dull laterally and anteriorly, mesonotum medially partially subshiny with some faint oliveceous to greenish metallic luster. Wing hyaline; costal vein ratio 0.37–0.43; M vein ratio 0.62–0.74; halter stem tan to yellowish tan; knob yellow to whitish yellow. Coxae gray; femora and tibia concolorous gray to blackish gray, densely microtomentose; tarsi generally yellowish; apical 2-3 tarsomeres darkened, brown to blackish brown. *Abdomen*: Generally thinly microtomentose; basal tergites largely gray to grayish white; tergite 5 of male subshiny to shiny, less densely microtomentose than anterior tergites. Male terminalia (Figs 31–33): Epandrium in posterior view (Fig. 31) as a broadly rounded, inverted U, width of dorsal portion narrower than expanded apices of lateral arms, lateral arms nearly straight, in lateral view wider ventrally; cercus in posterior view (Fig. 31) unevenly semilunate, much narrower dorsally than ventrally, densely setulose; aedeagus in lateral view (Fig. 33) as a parallelogram, basal margin shallowly emarginate, tapered toward apex, apex narrowly rounded, in ventral view (Fig. 32) expanded slightly laterally from narrow base to wider apex, apical margin bilobed, narrowly incised medially, each lateral lobe slightly narrower than aedeagal base, with thin, wing-like, narrow projections sub-basally; phallapodeme in lateral view (Fig. 33) more or less triangular, in ventral view (Fig. 32) longer than wide, bar-like with basal, sub-basal and apical crossbars, apical margin shallowly emarginate; ejaculatory apodeme in lateral view (Fig. 33) as an irregular comma with less arch; postgonite in lateral view (Fig. 33) narrowly pointed basally, thereafter becoming wider then apex narrowed, tapered, apex pointed, bearing 3-4 tiny setulae along basoposterior margin and 1 setula subapically; pregonite in lateral view (Fig. 33) moderately elongate, tapered, narrowed toward hypandrium, expanded toward aedeagus, aedeagal end truncate; hypandrium in ventral view (Fig. 32) wide, width nearly twice length, broadly and shallowly rounded along anterior margin with anterolateral shallowly and moreroundedly projected, obtusely pointed, shallow projections, angulately and moderately deeply incised along posterior margin medially, in lateral view (Fig. 33) irregularly clavate, very shallowly arched, expanded anteriorly, narrow, parallel sided posteriorly.

#### Type material.

The holotype male is *Gymnoclasiopa grecorum* Mathis and Zatwarnicki is labeled “**USA. A[las]K[a]**. Juneau: Thane Road (58°16.9'N, 134°22.4'W), 22 July 2011[,] D. & W. N. Mathis/USNM ENT 00117971 [plastic bar code label]/HOLOTYPE ♂ *Gymnoclasiopa grecorum* Mathis & Zatwarnicki, USNM [red].” The holotype is double mounted (minuten pin in a block of plastic), is in excellent condition, and is deposited in the USNM. Twenty-four paratypes (13♂, 11♀; USNM) bear the same locality label data as the holotype with dates from 20-22 July 2011. Other paratypes are as follows: UNITED STATES. Alaska. *Juneau*: Echo Cove (58°39.7'N, 134°54.4'W), 21 Jul 2011, D. and W. N. Mathis (1♂; USNM); Juneau, Mendenhall Valley, Riverside Rotary Park (58°22.8'N, 134°35.2'W), 21 Jul 2011, D. and W. N. Mathis (1♂; USNM). *Matanuska-Susitna*: Little Willow Creek (61°48.6'N, 150°05.8'W; 50 m), 25 Jul 2011, D. and W. N. Mathis (3♂; USNM); Sheep Creek (61°58.3'N, 150°05'W; 55 m), 10 Aug 2011, D. and W.N. Mathis (3♀; USNM); Talkeetna (Susitna River; 61°19.4'N, 150°07.2'W; 120 m), 10 Aug 2011, D. and W.N. Mathis (3♂, 1♀; USNM).

#### Type locality.

United States. Alaska. Juneau: Gastineau Channel, Thane Road (S Juneau; 58°16.9'N, 134°22.4'W).

#### Distribution 

**Figure 34.**
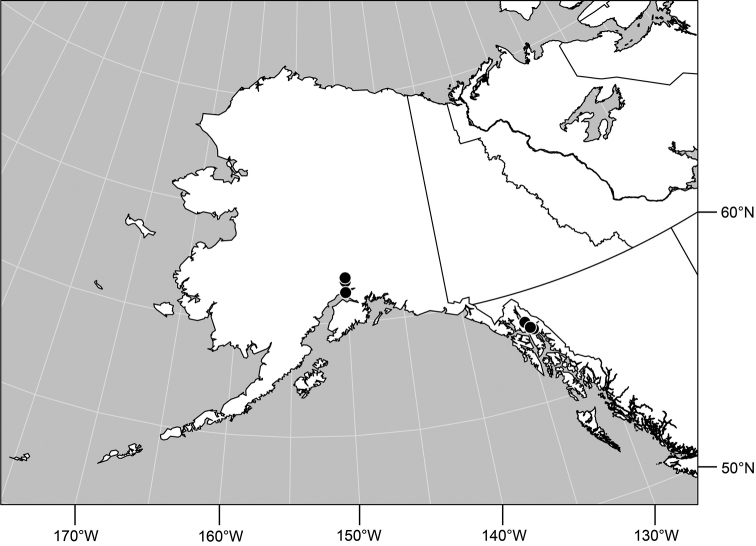
Distribution map of *Gymnoclasiopa grecorum* Mathis and Zatwarnicki sp. n.

(Fig. 34). *Nearctic*: United States (Alaska).

#### Etymology.

The species epithet, *grecorum*, is a plural genitive Latin patronym to honor and recognize the generous contributions of Richard Art and Karen Greco (nee Halliday) toward the improvement of mankind, their friendship, and their love of Alaska.

#### Remarks.

This species is similar to *Gymnoclasiopa subnubila* but is distinguished by the generally larger body size and by having the basal flagellomere black and the abdomen dull to subshiny (posterior tergites not as shiny as those of *Gymnoclasiopa subnubila*).

### Gymnoclasiopa matanuska

Mathis & Zatwarnicki sp. n.

urn:lsid:zoobank.org:act:D260E953-AB39-4BF4-9299-E1953B2D97FC

http://species-id.net/wiki/Gymnoclasiopa_matanuska

**Figures 35–36.**
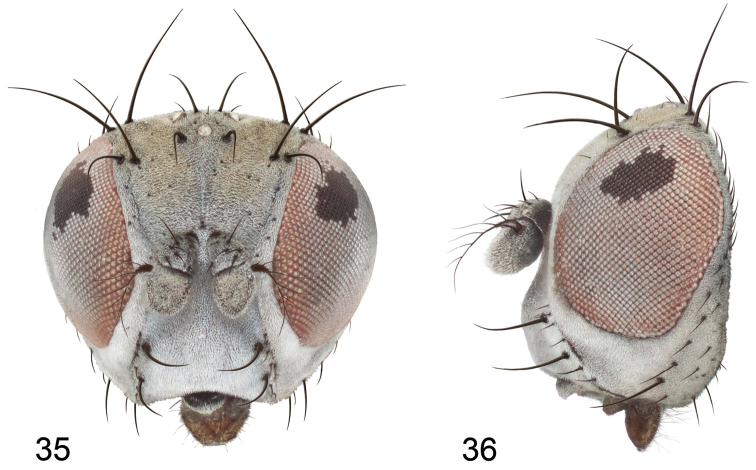
*Gymnoclasiopa matanuska* Mathis and Zatwarnicki, new species (USA. Alaska. Matanuska-Susitna: Palmer (Knik River)). **35** head, anterior view **36** same, lateral view.

Figs 35 [Fig F14] –40

#### Diagnosis.

**Figures 37–39. F14:**
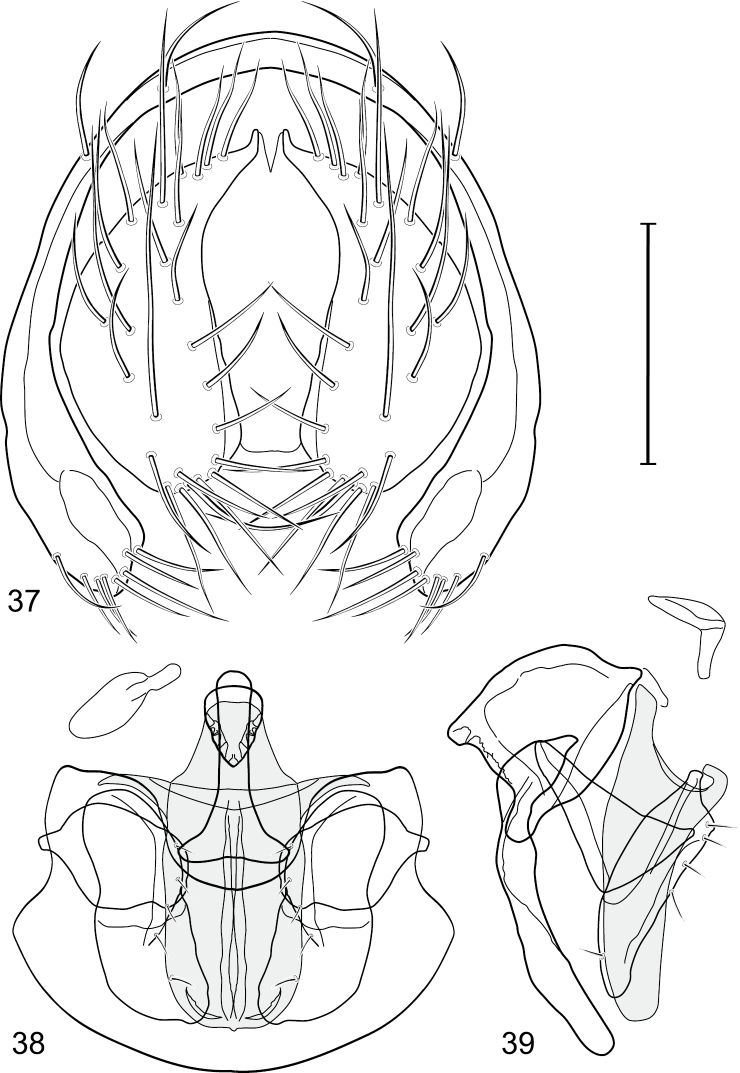
*Gymnoclasiopa matanuska* Mathis and Zatwarnicki, new species (USA. Alaska. Matanuska-Susitna: Palmer (Knik River)). **37** epandrium and cerci, posterior view **38** internal structures of male terminalia (aedeagus [shaded], phallapodeme, gonite, hypandrium), ventral view **39** same, lateral view. Scale bar = 0.1 mm.

This species is distinguished from congeners by the following combination of characters: Moderately small to medium-sized shore flies, body length 2.05–3.20 mm; dorsum generally microtomentose gray, microtomentum on abdomen sparser and finer. *Head* (Figs 35–36): Frons densely microtomentose, gray to blackish gray. Antenna black; basal flagellomere covered with silvery gray microtomentum; arista bearing 5 dorsal rays. Face somewhat flat, although antennal grooves, especially ventral margins, conspicuous; facial microtomentum distinctive, generally silvery white colored; gena relatively high, genal height greater than height of basal flagellomere; gena-to-eye ratio 0.28–0.33; gena silvery white, concolorous with face. Maxillary palpus black. *Thorax*: Thorax and abdomen generally densely microtomentose, gray to tannish gray, dull. Wing hyaline; costal vein ratio 0.38–0.52; M vein ratio 0.50–0.67; halter stem tan to yellowish tan; knob yellow to whitish yellow. Coxae, femora and tibia concolorous densely microtomentose gray to blackish dorsally, ventrally with some reddish coloration; fore- and hind tarsi mostly black; midtarsus with tarsomeres 1 and 2 somewhat yellowish to reddish, especially ventrally, apical tarsomeres black. *Abdomen*: Tergites largely gray, moderately densely microtomentose although less so than on mesonotum. Male terminalia (Figs 37–39): Epandrium in posterior view (Fig. 37) as a broadly rounded, inverted U, width of dorsal portion slightly narrower than lateral arms, lateral arms shallowly arched, gradually enlarged ventrally; cercus in posterior view (Fig. 37) unevenly semilunate, much narrower dorsally, pointed, ventral margin broadly rounded; aedeagus in lateral view (Fig. 39) slipper-like, base shallowly emarginate, tapered gradually toward apex, apex moderately narrowly bluntly rounded, in ventral view (Fig. 39) expanded laterally from narrow base on basal ¼, thereafter to apex almost parallel sided, apical margin slightly truncate, not bilobed, with thin, wing-like, narrow projections sub-basally; phallapodeme in lateral view (Fig. 39) more or less triangular, with broadly arched basal margin, in ventral view (Fig. 38) T-shaped, apical transverse bar robust, apical margin very shallowly emarginate; ejaculatory apodeme in lateral view L-shaped, in ventral view narrowly ovate with a thin, digitiform process; postgonite in lateral view (Fig. 39) narrowly tapered on extreme base, thereafter apically almost parallel sided to tapered apical 1/3, apex moderately narrowly rounded, bearing 3-4 tiny setulae along basoposterior margin and 1 setula subapically along margin toward hypandrium; pregonite in lateral view (Fig. 39) moderately elongate, tapered, acutely narrowed toward hypandrium, expanded toward aedeagus, aedeagal end truncate; hypandrium in ventral view (Fig. 38) wide, width twice length, broadly and shallowly rounded along anterior margin with shallowly extended, relatively wide, shallow, obtusely pointed lateral projections, shallowly emarginate along entire posterior margin, in lateral view (Fig. 39) angled basally, thereafter toward anterior margin irregularly parallel sided, narrow, bar-like.

#### Type material.

The holotype male of *Gymnoclasiopa matanuska* Mathis and Zatwarnicki is labeled “**USA. A[las]K[a]**. Mat[anuska]-Su[sitna]: Palmer (Matanuska River; 61°36.5'N, 149°04.1'W), 5 Aug 2002,D.&W.N.Mathis/USNM ENT 00183434 [plastic bar code label]/HOLOTYPE ♂ *Gymnoclasiopa matanuska* Mathis & Zatwarnicki, USNM [red].” The holotype is double mounted (minuten pin in a block of plastic), is in excellent condition, and is deposited in the USNM. Twenty-four paratypes (11♂, 13♀; USNM) bear the same label data as the holotype; 2 paratypes (1♂, 1♀; USNM) have the same locality data but with the date of 16 Aug 2012. Other paratypes are as follows: UNITED STATES. *ALASKA. Kenai Peninsula*: Kenai Fjord National Park, Exit Glacier (60°11.3'N, 149°37.6'W), D. and W. N. Mathis (1♀; USNM); Seward (21 km N; 60°17.2'N, 149°20.5'W; Snow River), 31 Jul 2002, D. and W. N. Mathis (4♂, 1♀; USNM); Swanson River Road (60°42.8'N, 150°48.9'W), 13 Aug 2012, D. and W. N. Mathis (1♂; USNM). *Matanuska-Susitna*: Knik River (61°27.8'N, 148°51.6'W), 5 Aug 2002, D. and W. N. Mathis (2♀; USNM); Lucile Lake (61°34.2'N, 149°28.6'W; 100 m), 15 Aug 2012, D. and W. N. Mathis (1♀; USNM); Palmer (Knik River; 61°31.2'N, 148°59.4'W), 6 Aug 2002, D. and W. N. Mathis (7♂, 1♀; USNM); Talkeetna (west, adjacent to Susitna River; 62°19.4'N, 150°07.2'W), 4-10 Aug 2003, 2011, D. and W. N. Mathis (2♀; USNM); Willow Creek (61°46.1'N, 150°04.2'W; 50 m), 10 Jul 2006, D. and W. N. Mathis (2♂; USNM). *Valdez-Cordova (Census Area)*: Chitina (61°30.9'N, 144°26.2'W), 18 Jun 1953, W. C. Frohne (3♀; USNM); Gulkana River (19.3 km N Glennallen; 62°16.1'N, 145°23.1'W), 9 Jul-7 Aug 2006, 2011, 2012, D. and W. N. Mathis (11♂, 12♀; USNM); Klutina River (mile 101; 61°57.2'N, 145°19.3'W; 315 m), 7 Aug 2012, D. and W. N. Mathis (15♂, 4♀; USNM); Lower Tonsina Valley (61°39.3'N, 144°39.5'W), 19 Aug 1953 (1♀; WSU); Valdez (4.8 km N; 61°05.8'N, 146°14.6'W), 8 Jul 2006, D. and W. N. Mathis (13♂, 6♀; USNM).

#### Type locality.

United States. Alaska. Matanuska-Susitna: Palmer (Matanuska River; 61°36.5'N, 149°04.1'W).

#### Other specimens examined.

Palearctic. MONGOLIA. *Bayan Ölgiy*: Bulgan Gol (47°05.3'N, 91°01.6'E; 2055 m), 7 Jul 2009, W. N. Mathis (1♂; USNM); Bulgan Gol (15 km N Bulgan; 47°02.4'N, 91°02.1'E; 2015 m), 9 Jul 2009, W. N. Mathis (3♂; USNM); Bulgan Gol (28 km N Bulgan; 47°06.9'N, 90°56.5'E; 2122 m), 8 Jul 2009, W. N. Mathis (1♀; USNM); Burgedtein Gol (47°31.9'N, 91°16.9'E; 2520 m), 4 Jul 2009, W. N. Mathis (1♂; USNM); Chigertei Gol (7 km W Dalum; 47°50.7'N, 90°38.6'E; 2165 m), 5 Jul 2009, W. N. Mathis (1♂; USNM); Gantsmodi Gol (27 km S Deluun; 47°37.5'N, 90°40.3'E; 2200 m), 5 Jul 2009, W. N. Mathis (8♂, 7♀; USNM); Ulaagchimy Davaa Gol (40 km N Bulgan; 47°20.9'N, 90°57.7'E; 2515 m), 6 Jul 2009, W. N. Mathis (1♀; USNM). *Khovd*: Bodovich Gol (8 km E Ikh Ulaan Davaa; 46°37.1'N, 92°13.8'E; 2545 m), 14 Jul 2009, W. N. Mathis (10♂, 3♀; USNM); Khovd, Buyant gol (47°58.5'N, 91°35.7'E; 1425 m), 2 Jul 2009, W. N. Mathis (1♂; USNM); Monkhkhayrkhan (47°03.3'N, 91°50.9'E; 2090 m), 16 Jul 2009, W. N. Mathis (2♂; USNM); Nevt Rashaa (9 km E Duut; 47°31.9'N, 91°42.3'E; 2050 m), 19 Jul 2009, W. N. Mathis (3♂, 1♀; USNM).

#### Distribution

**Figures 40.**
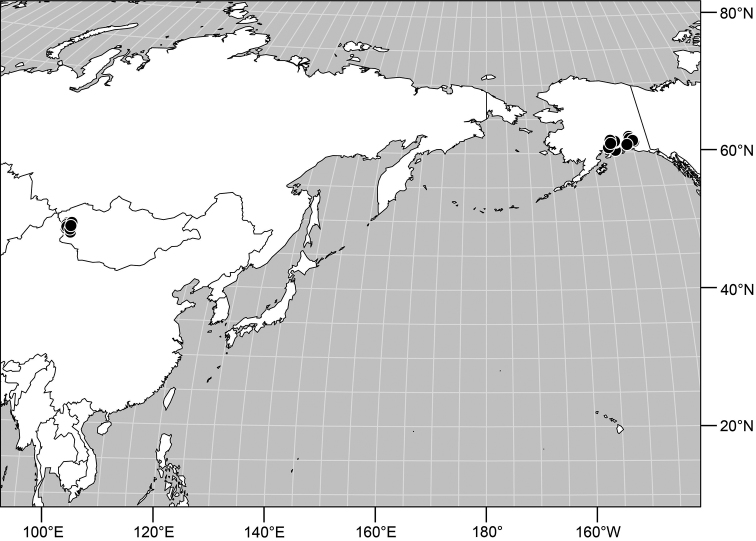
Distribution map of *Gymnoclasiopa matanuska* Mathis and Zatwarnicki sp. n.

(Fig. 40). *Nearctic*: United States (Alaska). *Palearctic*: Mongolia (Bayan Ölgiy, Khovd).

#### Etymology.

The species epithet, *matanuska*, refers to the glacier, river, and valley in southcentral Alaska where many specimens of the type series were collected along lotic aquatic systems. The name is apparently of Athabaskan origin and means a strong, gusty, northeast wind, which occasionally occurs in this region during the winter. The name is a noun in apposition.

#### Remarks.

Like a number of shore-fly species with Holarctic distributions, including *Gymnoclasiopa matanuska*, the Bering Strait is apparently a conduit between the Old and New Worlds.

Although similar to other congeners, especially *Gymnoclasiopa grecorum*, this species is distinguished by the black maxillary palpus and basitarsomere of the foreleg, the densely microtomentose gray to tannish gray body, especially on the thorax and abdomen, and the high genal height.

### Gymnoclasiopa parilis

(Cresson)

http://species-id.net/wiki/Gymnoclasiopa_parilis

Figs 41 –44

Ditrichophora parilis Cresson 1924: 160; 1942: 119 [review]. Wirth 1965: 739 [Nearctic catalog].
Gymnoclasiopa parilis . Mathis and Zatwarnicki 1995: 177 [generic combination; world catalog].

#### Diagnosis.

**Figures 41–43.**
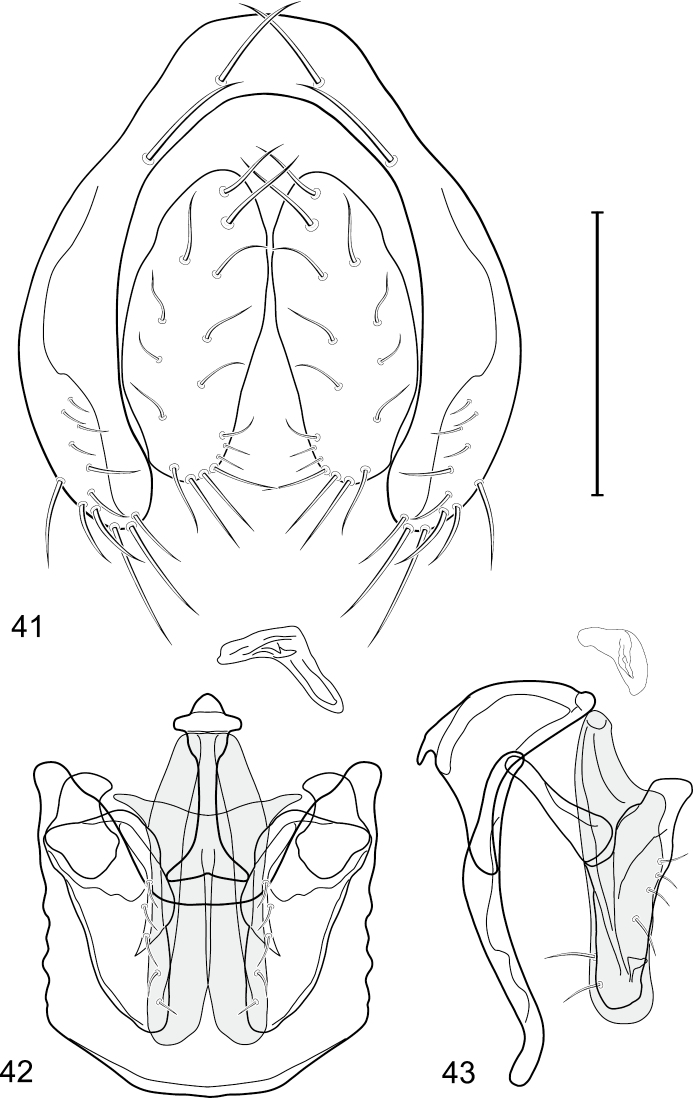
*Gymnoclasiopa parilis* (Cresson) (USA. Maine. Hancock: Cadillac Mountain). **41** epandrium and cerci, posterior view **42** internal structures of male terminalia (aedeagus [shaded], phallapodeme, gonite, hypandrium), ventral view **43** same, lateral view. Scale bar = 0.1 mm.

This species is distinguished from congeners by the following combination of characters: Small to moderately small shore flies, body length 1.60–2.60 mm, generally black with moderately to very sparsely gray microtomentose dorsally. *Head*: Frons moderately gray microtomentose on black background color; fronto-orbits gray. Scape black, pedicel mostly grayish black but with apex yellowish red; basal flagellomere blackish gray dorsoapically, yellowish to reddish basoventrally; arista bearing 5 dorsal rays. Face somewhat flat, although antennal grooves, especially ventral margins, relatively conspicuous; facial microtomentum distinctive, generally silvery white; genal height greater than height of basal flagellomere, gena-to-eye ratio 0.13–0.15, silvery white, concolorous with face. Maxillary palpus black. *Thorax*: Mesonotum thinly microtomentose, subshiny with some metallic luster, especially medially; grayish black; pleural areas gray to brownish gray, subshiny. Wing hyaline; costal vein ratio 0.36–0.43; M vein ratio 0.52–0.61; halter stem brown to blackish brown; knob whitish yellow to yellow. Coxae, femora and tibia concolorous grayish black to black, microtomentum sparse; tarsi mostly yellow, apical tarsomeres darker, more grayish black. *Abdomen*: Tergites largely shiny black, very sparsely microtomentose. Male terminalia (Figs 41–43): Epandrium in posterior view (Fig. 41) as a moderately rounded, inverted U with the base more narrowly formed, width of dorsal portion slightly narrower than lateral arms, lateral arms shallowly arched, setulae more clustered at ventral margin; cercus in posterior view (Fig. 41) elongate, evenly oval, dorsal margin very slightly more narrowly rounded than ventral margin, setulae more clustered at ventral margin; aedeagus in lateral view (Fig. 43) slipper-like, base shallowly emarginate, tapered gradually toward apex, apex moderately broadly rounded, in ventral view (Fig. 42) expanded laterally from narrow base on basal 1/3, thereafter to apex almost parallel sided, apical margin moderately deeply incised medially, bilobed; phallapodeme in lateral view (Fig. 43) more or less irregularly triangular, extension toward hypandrium more elongate than angle towards aedeagal base, in ventral view (Fig. 42) I-shaped, apical crossbar robust, wider than basal crossbar, apical margin very shallowly emarginate; ejaculatory apodeme in lateral view robustly comma-shaped, in ventral view L-shaped; postgonite in lateral view (Fig. 43) robustly bar-like, basal 1/3 angulate, narrower than extended, mostly parallel-sided apical 2/3, bearing 3-4 setulae along posterior margin and 2 setulae subapically along anterior margin, in ventral view (Fig. 42) as an elongate triangle, wide basally, tapered to narrowly rounded apex; pregonite in lateral view (Fig. 43) moderately elongate, straight, clavate, wider apically than narrow base, in ventral view (Fig. 42) robustly J-shaped with pointed, curved base and expanded apex; hypandrium in ventral view (Fig. 42) almost quadrate, as wide as long, anterior margin shallowly curved, lateral margins curvey, posterior margin broadly and moderately deeply emarginate, forming wide pocket, in lateral view (Fig. 43) narrowly elongate, shallowly sinuous.

#### Type material.

The holotype male of *Ditrichophora parilis* Cresson is labeled “Bar Harbor 4 VIII. 18. [August 18; date handwritten] Me [Maine. Hancock:]/♂/M. C. Z. Type 31758 [red; number handwritten]/TYPE Ditrichophora PARALIS [sic; *parilis*] E. T. Cresson, Jr. [maroon; species name handwritten].” The holotype is directly pinned, is in good condition (thorax partially broken where pin is inserted), and is deposited in the MCZ (31758). Cresson (1924: 160) noted that C. W. Johnson was the collector.

#### Type locality.

United States. Maine. Hancock: Bar Harbor (44°18.7'N, 68°12.2'W).

#### Other specimens examined.

Nearctic. CANADA. *BRITISH COLUMBIA*. Waterfall at Peterson Creek # 4, Alaska Highway DC 445 (52°24'N, 121°38'W), 29 Jun 1978, P. H. Arnaud, Jr. (1♀; CAS).

*NORTHWEST TERRITORIES*. Aklavik (68°13.6'N, 135°0.7'W), 12 Jun-15 Sep 1931, O. Bryant (1♂, 3♀; USNM).

UNITED STATES. *ALASKA. Fairbanks North Star*: Fairbanks, Lake Ballaine (64°52.2'N, 147°49.5'W; 160 m), 2 Aug 2011, D. and W. N. Mathis (1♂, 3♀; USNM). *Kenai Peninsula*: Kenai Lake (60°20.5'N, 149°22.2'W; Primrose Campground), 31 Jul 2002, 2003, D. and W. N. Mathis (2♂, 2♀; USNM); Kenai River, Jim’s Landing (60°28.9'N, 150°06.9'W), 3 Aug 2002, D. and W. N. Mathis (2♂; USNM); Skilak Lake (60°26.3'N, 150°19.4'W), 3 Aug 2002, D. and W. N. Mathis (1♀; USNM). *Matanuska-Susitna*: Eklutna (Knik Arm; 61°28.2'N, 149°21.4'W), 7 Aug 2002, D. and W. N. Mathis (1♂; USNM); Glennallen (96.5 km W Glennallen; 61°55.6'N, 147°13.6'W), 7 Aug 2012, D. and W. N. Mathis (1♀; USNM); Knik River (61°27.8'N, 148°51.6'W), 5 Aug 2002, D. and W. N. Mathis (21♂; USNM); Matanuska (61°32.5'N, 149°13.8'W; rotary trap), 29 Apr 1944, J. C. Chamberlin (1♂, 4♀; USNM); Matanuska Flats (N Palmer; 61°16'N, 150°16'W), 17 Jul 1971, B. A. Foote (1♂; USNM); Sheep Creek (61°58.3'N, 150°05'W; 55 m), 10 Aug 2011, D. and W.N. Mathis (3♂, 5♀; USNM); Talkeetna (62°18.9'N, 150°06.3'W), 10 Aug 2011, D. and W. N. Mathis (1♀; USNM); Willow Creek (61°46.1'N, 150°04.2'W; 50 m), 10 Jul 2006, D. and W. N. Mathis (1♂; USNM). *Southeast Fairbanks (Census Area)*: Delta Junction (8 km S; 63°51.5'N, 145°44.6'W), 11 Aug 2003, D. and W. N. Mathis (1♂; USNM); Gardiner Creek Camp, Alaska Highway DC 1253 (62°51.5'N, 141°28'W), 5 Aug 1978, P. H. Arnaud, Jr. (1♂; CAS); Tok River, GJ-97.9 (62°21.8'N, 142°50.5'W), 4 Aug 1978, P. H. Arnaud, Jr. (1♀; CAS). *Valdez-Cordova (Census Area)*: Chitina (61°30.9'N, 144°26.2'W), 15-18 Jun 1953, W. C. Frohne (2♂, 2♀; USNM; WSU); Lower Tonsina Valley (61°39.3'N, 144°39.5'W), 19 Aug 1953, W. C. Frohne (1♀; WSU); Tolsona Creek State Campground, Glenn Highway, A 173 (62°03.9'N, 145°59.8'W), 31 Jul 1978, P. H. Arnaud, Jr. (2♂, 1♀; CAS). *Yukon-Koyukuk (Census Area)*: Circle Hot Springs (65°49.5'N, 144°03.6'W), 26 Jul 1971, B. A. Foote (2♂, 3♀; CMNH, USNM).

*CALIFORNIA. San Mateo*: Stanford University (37°24'N, 122°14.5'W), 15 Jul, A. L. Melander (1♀; ANSP).

*CONNECTICUT. Litchfield*: Kent Falls (41°46.7'N, 73°25'W), 28 Aug 1940, A. L. Melander (1♂; USNM).

*MAINE. Hancock*: Bar Harbor (44°23.3'N, 68°12.3'W), 2 Jul 1921, C. W. Johnson (1♀; ANSP); Cadillac Mountain (44°21.1'N, 68°13.4'W), 25 Jul 1914 (1♂; ANSP). *Somerset*: Caratunk (45°14'N, 69°59.4'W), 2 Aug 1950, A. H. Sturtevant (1♀; USNM).

*MASSACHUSETTS. Hampden*: Chester (42°16.4'N, 72°56.6'W), 12 Jul 1912, C. W. Johnson (1♂; ANSP).

*NEW HAMPSHIRE. Coos*: Dolly Copp (44°19.9'N, 71°13.1'W), 13 Jul 1931, A. L. Melander (1♀; USNM). *Grafton*: White Mountains, Stinson Lake (43°51.7'N, 71°48.5'W), 23 Jul 1961, W. W. Wirth (1♂, 1♀; USNM).

*NEW YORK. Cattaraugus*: Stoddard Hollow (42°04'N, 78°45.5'W; 550-580 m), 10 Aug 1961, D. L. Deonier (7♂, 7♀; USNM). *Hamilton*: Shanty Brook (43°17.2'N, 74°33.4'W; 520 m), 5 Aug 1961, D. L. Deonier (4♂, 2♀; USNM).

#### Distribution

**Figure 44.**
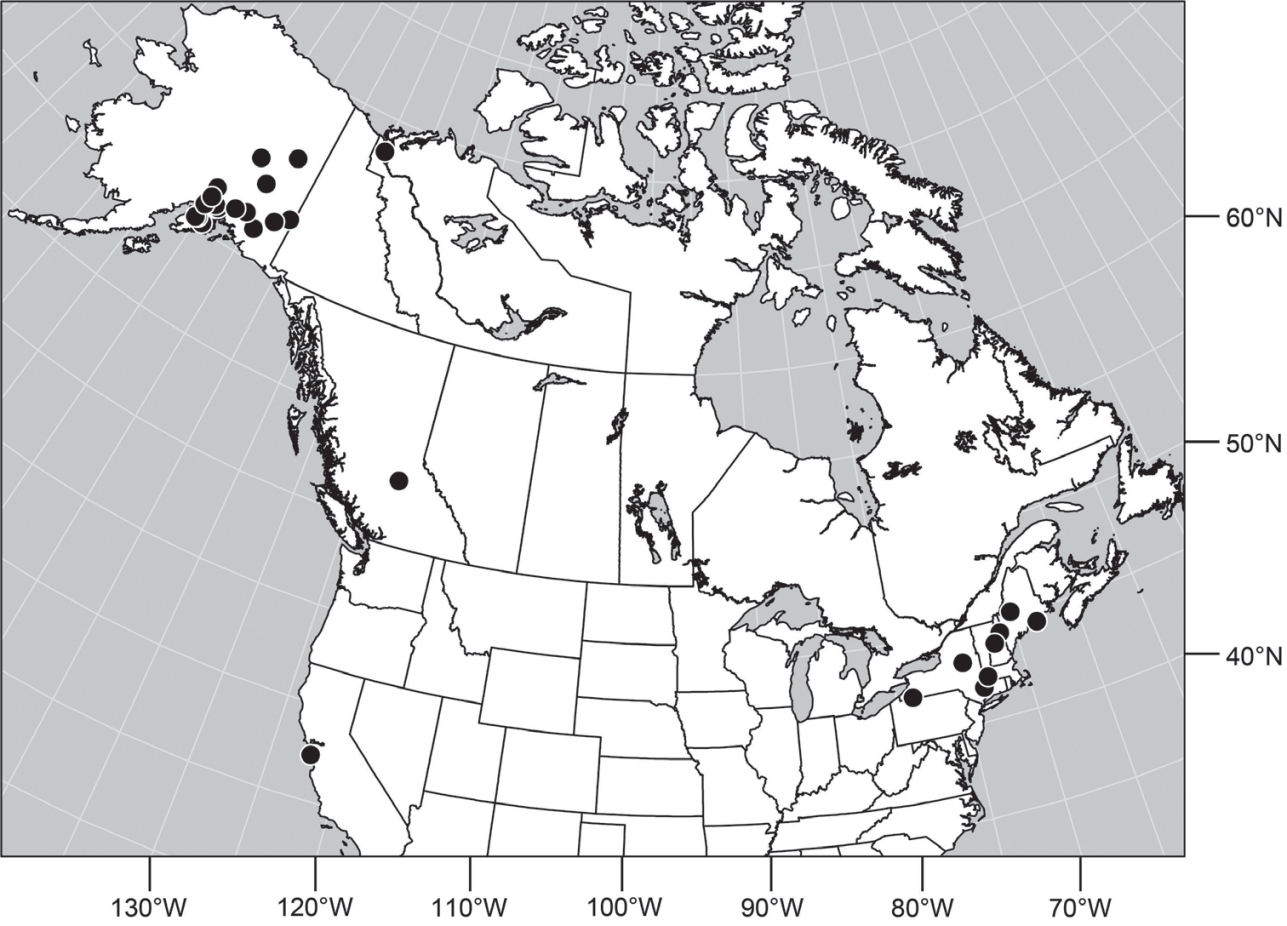
Distribution map of *Gymnoclasiopa parilis* (Cresson).

(Fig. 44). Nearctic: Canada (British Columbia, Northwest Territories, Quebec, Yukon Territory), United States (Alaska, California, Connecticut, Maine, Massachusetts, New Hampshire, New York, ?Vermont [literature record, not confirmed; Cresson 1924: 160], Washington).

#### Remarks.

In collections that we studied, this was the species that was most often misidentified. Although similar to other congeners, it is distinguished most by the black maxillary palpus and tan to yellowish basitarsomere of the foreleg and halter stem. From *Gymnoclasiopa grecorum*, which also shares these character states just noted, it is distinguished by the ventrally reddish antennae (especially the basal flagellomere) and the black, subshiny postpronotum, notopleuron, and anepisternum.

### Gymnoclasiopa pulchella

(Meigen)

http://species-id.net/wiki/Gymnoclasiopa_pulchella

**Figures 45–47.**
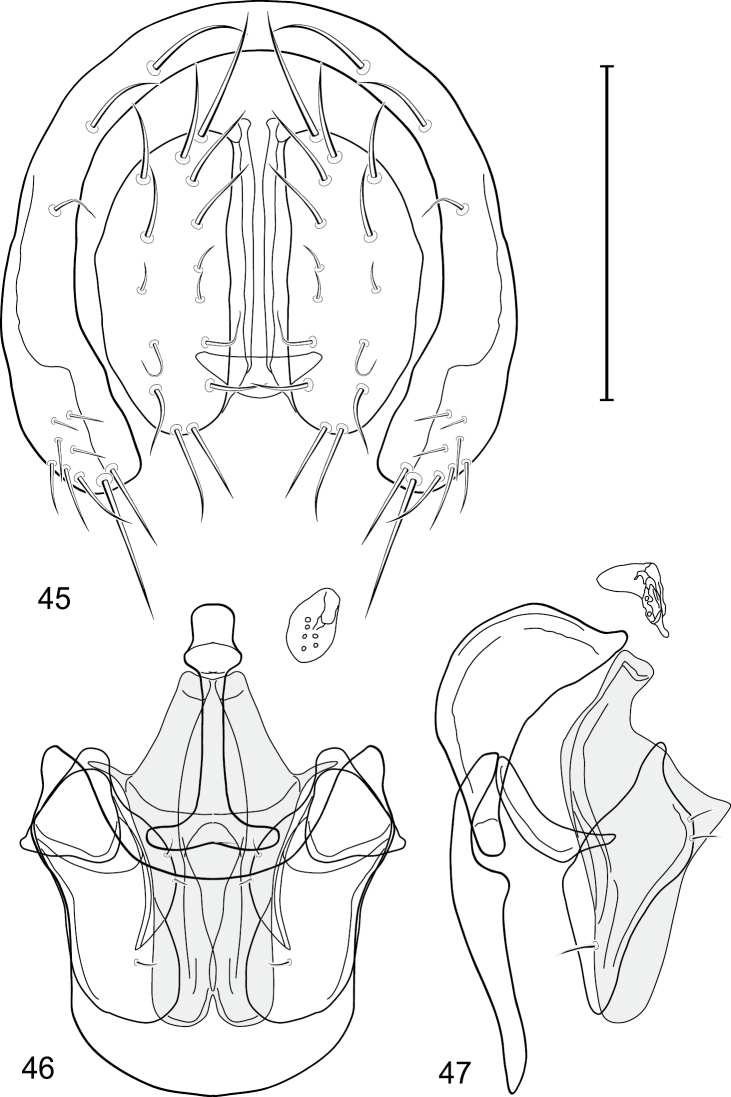
*Gymnoclasiopa pulchella* (Meigen) (Poland. Wroclaw-Lesnica). **45** epandrium and cerci, posterior view **46** internal structures of male terminalia (aedeagus [shaded], phallapodeme, gonite, hypandrium), ventral view **47** same, lateral view. Scale bar = 0.1 mm.

Figs 45 [Fig F19] –49

Notiphila pulchella Meigen 1830: 70.
Hydrellia pulchella . Macquart 1835: 525 [generic combination].
Clasiopa pulchella . Becker 1905: 195 [generic combination].
Discocerina pulchella . Czerny 1909: 268 [generic combination]. Deonier 1964: 121 [key, Iowa]; 1965: 502 [ecology].
Ditrichophora (Gymnoclasiopa) pulchella . Cresson 1942: 121 [generic combination]. Wirth 1965: 739 [Nearctic catalog]. Cole 1969: 398 [fauna, western North America].
Gymnoclasiopa pulchella . Mathis and Zatwarnicki 1995: 178 [generic combination; world catalog].
Clasiopa fulgida Becker 1896: 156. Becker 1902: 297 [synonymy].

#### Diagnosis.

**Figure 48. F19:**
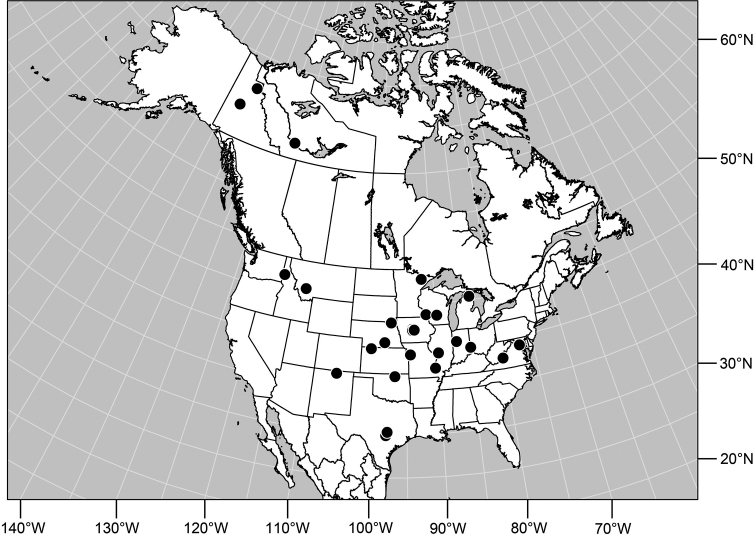
Distribution map of *Gymnoclasiopa pulchella* (Meigen).

This species is distinguished from congeners by the following combination of characters: Small to moderately small shore flies, body length 1.60–2.45 mm; generally coloration of males and females sexually dimorphic to a degree with females generally less microtomentose. *Head*: Frons of male densely microtomentose, moderately intensely whitish gray to white, that of female moderately microtomentose, whitish gray microtomentose anterior, becoming less microtomentose, more blackish posteriorly; proclinate fronto-orbital seta 1. Antenna generally yellowish orange (sometimes apex of basal flagellomere and scape darkened); arista bearing 4–6 dorsal rays. Face relatively flat; antennal grooves, especially ventral margins, inconspicuous; face of male densely microtomentose, intensely yellow, that of female less densely microtomentose, silvery white; facial setae inserted close to parafacials, aligned vertically; gena very short, gena-to-eye ratio 0.065–0.072. Maxillary palpus yellowish orange. *Thorax*: Anterolateral area of mesonotum, just mediad of area from postpronotum through notopleuron, thinly to very thinly microtomentose, subshiny to shiny with some metallic luster; lateral mesonotal area from and including postpronotum and notopleuron in males with thin microtomentum whitish to grayish, similar to central mesonotal microtomentum, not brown; mesonotum of female more thinly microtomentose, appearing blackish with whitish gray microtomentum more sparse; pleural areas generally shiny black with some very thin microtomentum ventrally. Wing mostly hyaline; costal section II conspicuously longer than costal section III by about twice length; costal vein ratio 0.44–0.47; M vein ratio 0.43–0.54; halter stem dark brown; knob white to whitish yellow. Femora black; tibiae blackish brown except for basal and apical extremities black; tarsi mostly yellowish orange. *Abdomen*: Tergites shiny black. Male terminalia (Figs 45–48): Epandrium in posterior view (Fig. 45) as a broadly rounded, inverted U, width of dorsal portion slightly narrower than lateral arms, lateral arms shallowly arched, in lateral view wider ventrally; cercus in posterior view (Fig. 45) almost evenly semilunate, robustly developed at dorsal and ventral margins; aedeagus in lateral view (Fig. 47) slipper-like, base wide with moderately narrow, thumb-like projection at connection with phallapodeme, tapered more or less evenly to moderately rounded apex, in ventral view (Fig. 47) with basal 1/3 expanded laterally from narrow base, thereafter to apex slightly tapered then parallel to bilobed apex, each lateral lobe as wide as aedeagal base, with thin, short, wing-like, narrow projections sub-basally; phallapodeme in lateral view (Fig. 47) robustly lunate, basal margin broadly and conspicuously rounded, narrowed at each apex, in ventral view (Fig. 46) longer than wide, T-like, with long, apical crossbar, apical margin shallowly emarginate; ejaculatory apodeme in lateral view L-shaped, in ventral view broadly ovate; postgonite in lateral view (Fig. 47) as a parallelogram, acutely pointed basally and apically, bearing 2-3 setulae along basoposterior margin and 1 setula subapically along margin toward hypandrium; pregonite in lateral view (Fig. 47) moderately elongate, almost parallel sided, tapered, narrowed toward aedeagus, narrowly pointed; hypandrium in ventral view (Fig. 46) wide, width almost twice length, broadly and shallowly rounded along anterior margin without lateral projections anterolaterally, but with posterolateral, robust projections, forming a deeply and widely incised posterior margin medially, in lateral view (Fig. 47) nearly straight, narrow, posterior with dished out emargination on posterior 1/3, thereafter anteriorly narrow, very slightly tapered toward anterior margin.

#### Type material.

The lectotype male of *Notiphila pulchella* Meigen (designated by Cresson 1925: 256) is labeled “pulchella Coll. [Wilhelm von] Winth[em]. [pulchella handwritten]/pulchella [handwritten]/TYPE [dark red].” The lectotype is double mounted, is in poor to fair condition (part of frons, thorax, and rest of abdomen wet; tiny balls on distal part of wing; forelegs with both coxae, left tibia and femur; hindlegs covered with dark brown substance; right wing missing; distal portion of abdomen removed and stored in plastic microvial with glycerin), and is deposited in the NMW.

The holotype female of *Clasiopa fulgida* Becker is labeled “[Romania. Mehedinți:] Orsova [44°43.5'N, 22°23.8'E] V. 37647 [submarginal border; “Orsova” printed; number handwritten]/Holotypus [red, printed].” The holotype is double mounted, is in moderate condition (abdomen missing), and is deposited in the ZMHU.

#### Type locality.

Not given, Wilhelm von Winthem collection (? Germany, perhaps Hamburg).

#### Other specimens examined.

Nearctic. CANADA. *NORTHWEST TERRITORIES*. Aklavik (66°13.1'N, 135°0.3'W), 5 Aug 1930, O. Bryant (1♂; CAS). Fort Simpson (61°50.8'N, 121°21'W), 16 Aug 1929, O. Bryant (1♂; CAS).

*YUKON TERRITORY*. Clear Lake, Klondike Loop (63°47'N, 137°18'W), 5 Jul 1978, P. H. Arnaud, Jr. (2♂, 2♀; CAS).

UNITED STATES. *IDAHO. Latah*: Laird Park (6.4 km NE Harvard; 46°56.5'N, 116°38.8'W), 6 Jul 1978, R. S. Zack (1♂; WSU).

*ILLINOIS. Macoupin*: Carlinville (39°16.8'N, 89°52.9'W), 5 Sep 1952, M. R. Wheeler (3♂, 5♀; USNM).

*INDIANA. Tippecanoe*: Lafayette (40°25'N, 86°52.5'W), 17 May-26 Jul 1916, 1922, E. V. Stafford (3♂, 2♀; ANSP, USNM).

*IOWA. Boone*: Fraser (1 km SW; 42°07'N, 93°58'W), 4 Jul 1960, D. L. Deonier (2♂, 1♀; USNM); Ledges State Park (41°59'N, 93°53.2'W), 13 May-16 Aug 1960, 1968, D. L. Deonier, J. L. Laffoon (35♂, 22♀; USNM). *Story*: Ames (42°02.1'N, 93°37.2'W), 17 Jul 1960, D. L. Deonier (5♂, 2♀; USNM).

*KANSAS. Decatur*: Sappa State Park (39°50.2'N, 100°29.7'W), 31 Aug 1961, D. L. Deonier (3♂, 2♀; USNM).

*MICHIGAN. Saginaw*: Saginaw (45°25.2'N, 83°57'W), 18 Jun 1952, R. R. Dreisbach (1♂; USNM).

*MINNESOTA. Houston*: Houston (43°45.8'N, 91°34.1'W), 28 May 1939, H. E. Milliron (1♀; USNM). *St. Louis*: Eagles Nest (47°50.4'N, 92°05.8'W), 5 Jul 1957, W. V. Balduf (1♀; USNM).

*MISSOURI. Jackson*: Atherton (39°11.2'N, 94°18.3'W), 21 Aug 1915, C. F. Adams (3♂, 9♀; ANSP, USNM). *Madison*: Fredericktown (37°33.6'N, 90°17.6'W), 4 Sep 1952, A. H. Sturtevant (2♂; USNM); Oak Grove (37°34.4'N, 90°23.7'W), 4 Sep 1952, A. H. Sturtevant (1♂; USNM); Silver Mine (37°33.4'N, 90°29.1'W), 4 Sep 1952, A. H. Sturtevant (1♂; USNM).

*MONTANA. Silver Bow*: Pipestone Pass (45°51.6'N, 112°26'W), 3 Jul 1925, A. L. Melander (1♂; ANSP).

*NEBRASKA. Adams*: Hastings (40°35.2'N, 98°23.3'W), 22 Aug 1950, M. R. Wheeler (6♀; USNM).

*NEW MEXICO. Taos*: Questa, Red River (36°42.2'N, 105°34.7'W), 26 May 1969, W. W. Wirth (1♂; USNM).

*OHIO. Preble*: Hueston Woods State Park (39°34.4'N, 84°44.5'W), 25 Jun 1975, J. Regensburg (1♂; USNM).

*OKLAHOMA. Osage*: Burbank (36°41.7'N, 96°43.9'W), 13 Sep 1952, A. H. Sturtevant (2♂; USNM).

*SOUTH DAKOTA. Yankton*: Yankton (42°52.3'N, 97°23.8'W), 25 Jun 1948, A. H. Sturtevant (1♂, 1♀; USNM).

*TEXAS. Hays*: San Marcos (29°53'N, 97°56.5'W), 8 Nov 1962, A. H. Sturtevant (1♀; USNM). *Travis*: Austin (30°16'N, 97°44.6'W), 15 Oct 1950, M. R. Wheeler (9♂, 3♀; USNM).

*VIRGINIA. Alleghany*: Covington (37°47.6'N, 79°59.6'W), 25 Jun 1933, A. L. Melander (1♂; ANSP). *Arlington*: Chain Bridge (38°55.5'N, 77°06.5'W), 20 Aug 1922, J. R. Malloch (1♂; USNM).

*WISCONSIN. Columbia*: Dells (43°37.5'N, 89°45.9'W), 8 Jul 1933, A. L. Melander (1♂; USNM).

#### Distribution

(Fig. 48). *Nearctic*: Canada (Alberta, Northwest Territories, Ontario, Yukon Territory). United States (Idaho, Illinois, Indiana, Iowa, Kansas, Michigan, Minnesota, Missouri, Nebraska, New Mexico, Ohio, Oklahoma, South Dakota, Texas, Virginia, Wisconsin). *Palearctic*: Austria, Belgium, Czech Republic, Morocco, Romania, Russia (European Territory), Spain.

#### Remarks.

This species is rarely collected and may be uncommon in nature. In the Delmarva States (United States), this species occurs along the coastal plain and in the Alleghany and is expected to be found between these zones.

Although uncommon, this species is relatively distinctive and is similar to *Gymnoclasiopa bohemanni*, in having pale, usually yellowish maxillary palpi, antennae, and foretibiae. This species is distinguished from *Gymnoclasiopa bohemanni* as follows (also see key): The anterolateral area of the mesonotum, just mediad of the area from the postpronotum through the notopleuron is thinly microtomentose and is subshiny to shiny with some metallic luster; the lateral mesonotal area from and including the postpronotum and notopleuron in males is thinly whitish microtomentose, similar to the central mesonotal microtomentum; and the midtibiae are dark colored.

**Figures 49–51.**
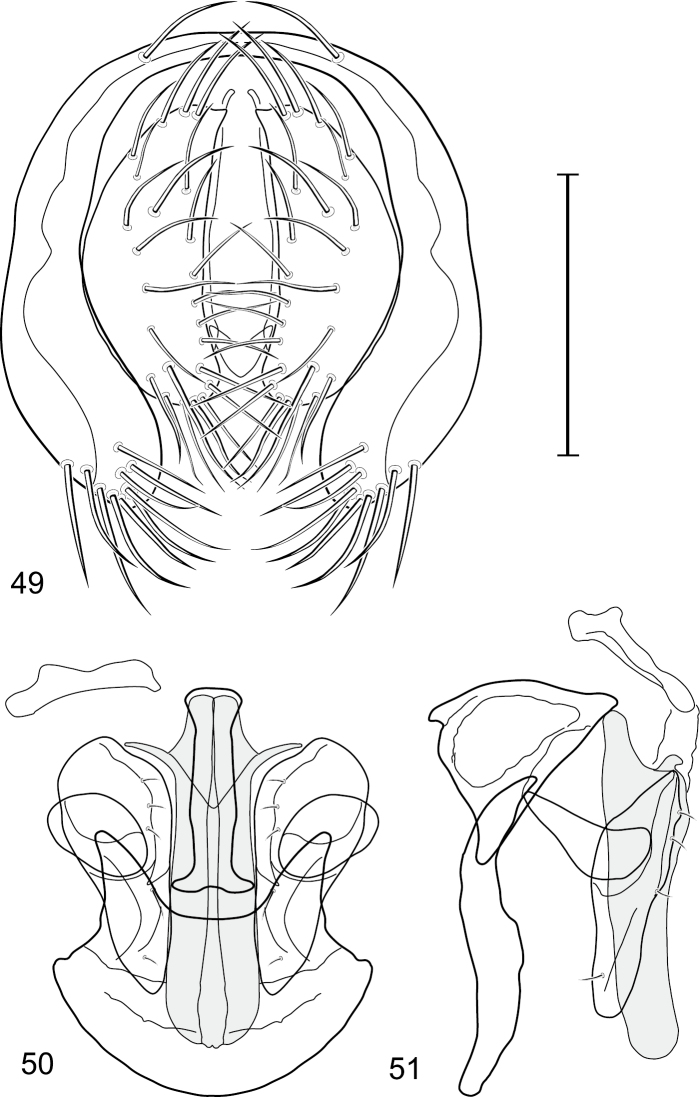
*Gymnoclasiopa subnubila* (Cresson) (Canada. British Columbia. Hope, Silver Lake). **49** epandrium and cerci, posterior view **50** internal structures of male terminalia (aedeagus [shaded], phallapodeme, gonite, hypandrium), ventral view **51** same, lateral view. Scale bar = 0.1 mm.

### Gymnoclasiopa subnubila

(Cresson)

http://species-id.net/wiki/Gymnoclasiopa_subnubila

Figs 49 –52

Ditrichophora subnubila Cresson 1940: 6; 1942: 120 [review]. Wirth 1965: 739 [Nearctic catalog]. Cole 1969: 398 [fauna, western North America].
Gymnoclasiopa subnubila . Mathis and Zatwarnicki 1995: 178 [generic combination; world catalog].

#### Diagnosis.

**Figure 52.**
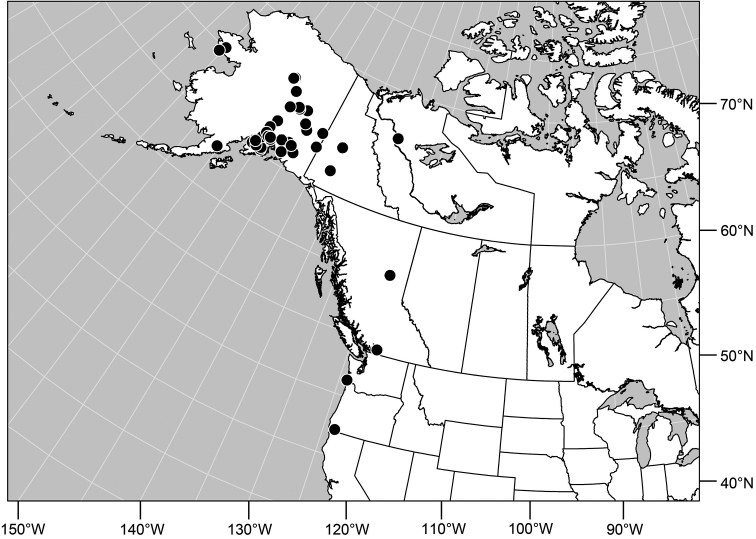
Distribution map of *Gymnoclasiopa subnubila* (Cresson).

This species is distinguished from congeners by the following combination of characters: Small to moderately small shore flies, body length 1.75–2.95 mm, head and thorax generally moderately to densely microtomentose, gray, abdomen thinly invested with microtomentum, subshiny to shiny black. *Head*: Frons densely microtomentose, mostly tannish gray to gray; fronto-orbits gray. Scape black, pedicel mostly grayish black to blackish gray; basal flagellomere blackish gray dorsoapically, yellowish to reddish basoventrally; arista bearing 5 dorsal rays. Face somewhat flat, although antennal grooves, especially ventral margins, somewhat conspicuously impressed; facial microtomentum distinctive, generally silvery yellow, shiny; genal moderately high, height greater than height of basal flagellomere, gena-to-eye ratio 0.15–0.17, whitish gray, contrasted with silvery yellow face. Maxillary palpus yellow to yellowish red. *Thorax*: Mesonotum tannish gray to brownish gray, moderately microtomentose, some areas somewhat subshiny; pleural areas gray, more densely microtomentose than mesonotum. Wing hyaline; costal vein ratio 0.44–0.56; M vein ratio 0.54–0.57; halter stem yellowish to brown, knob yellowish white to yellow. Coxae gray; femora and tibia concolorous blackish gray to gray, apices yellowish; tarsi mostly yellow, apical tarsomeres darker, more grayish black. *Abdomen*: Basal tergites and medially gray to tannish gray, laterally and apical tergites shiny black, very sparsely microtomentose. Male terminalia (Figs 49–52): Epandrium in posterior view (Fig. 49) as a broadly formed, inverted U, somewhat rounded quadrate, with the base more narrowly formed, dorsal portion more thinly developed than lateral arms, lateral arms shallowly arched to nearly straight, curved medially subapically, enlarged ventrally, broadly rounded, setulae more clustered at ventral margin; cercus in posterior view (Fig. 49) elongate,semilunate, lateral margins arched, medial margins nearly straight, nearly uniformly setulose but slightly more clustered at ventral margin; aedeagus in lateral view (Fig. 52) slongate, narrowly slipper-like, nearly straight, base shallowly emarginate, tapered very slightly toward apex, long margins nearly parallel sided, apex moderately broadly rounded, in ventral view (Fig. 50) expanded sub-basally with narrow, laterally flaring processes, thereafter very slightly expanded to apex, essentially parallel sided, apex broadly rounded; phallapodeme in lateral view (Fig. 51) more or less irregularly triangular, extension toward hypandrium slightly more elongate than extension towards aedeagal base, in ventral view (Fig. 50) T-shaped, apical crossbar moderately robust, length of each flared arm about half width of stem; ejaculatory apodeme in lateral view straight, base expanded, extended process digitiform, in ventral view almost bar-like with bump near midlength; postgonite in lateral view (Fig. 51) irregularly bar-like, pointed basally, widest just before midlength, thereafter slightly tapered, apex robustly digitiform, bearing 3 setulae along posterior margin and 1 setula subapically along anterior margin, in ventral view (Fig. 50) as an elongate triangle, wide basally, length of tapered sides about twice basal width, apex narrowly rounded, lateral margins shallowly emarginate, straight medially; pregonite in lateral view (Fig. 51) clavate, pointed on portion toward hypandrium, rounded on opposite end, in ventral view (Fig. 50) ovate with rounded pointed; hypandrium in ventral view (Fig. 50) with anterior margin broadly rounded, thereafter posteriorly with lateral margin concave, posterior margin moderately deeply and widely U-shaped, in lateral view (Fig. 51) narrowly elongate, shallowly sinuous.

#### Type material.

The holotype male of *Ditrichophora subnubila* Cresson is labeled “Ilwaco WASH July 1917 ALMelander/TYPE Ditrichophora SUBNUBILA E. T. Cresson, Jr. [red; species number and name handwritten].” The holotype is double mounted (minuten pin in a rectangular card), is in excellent condition (some cephalic setae missing or misoriented), and is deposited in the ANSP (6550).

#### Type locality.

United States. Washington. Pacific: Ilwaco (46°18.5'N, 124°02.6'W).

#### Other specimens examined.

Nearctic. CANADA. *BRITISH COLUMBIA*. Hope, Silver Lake (49°22'N, 121°29'W), 2 Jul 1968, W. W. Wirth (1♂, 1♀; USNM). Pine Pass (37 km NE; highway 97; 55°30'N, 122°40'W), 25 Jun 1978, P. H. Arnaud, Jr. (5♂, 3♀; CAS).

*NORTHWEST TERRITORIES*. Good Hope, Mackenzie River (66°15'N, 128°38'W), 23 Aug 1929, O. Bryant (1♀; USNM).

*YUKON TERRITORY*. Aishihik River, Alaska Highway DC 996.8 (61°40.3'N, 137°28.3'W), 7 Aug 1978, P. H. Arnaud, Jr. (25♂, 12♀; CAS). Clear Lake, Klondike Loop (63°47'N, 137°18'W), 5 Jul 1978, P. H. Arnaud, Jr. (1♂; CAS).

UNITED STATES. *ALASKA. Anchorage*: Mirror Lake (61°25.7'N, 149°24.9'W), 29 Jun-5 Aug 2002, 2006, D. and W. N. Mathis (6♂, 1♀; USNM). *Fairbanks North Star*: Colorado Creek, Chena Hot Springs (65°03.2'N, 146°02.9'W), 11 Jul 1978, P. H. Arnaud, Jr. (2♀; CAS); Creamer’s Field (64°51.7'N, 147°44.3'W; 160 m), 3 Aug 2011, D. and W. N. Mathis (1♂, 1♀; USNM); Fairbanks, Lake Ballaine (64°52.2'N, 147°49.5'W; 160 m), 2 Aug 2011, D. and W. N. Mathis (9♂; USNM); Fairbanks (32 km E: 64°50'N, 147°24'W), 28 Jul 1971, B. A. Foote (3♂, 3♀; CMNH, USNM); Steese Highway (milepost 10: 64°56'N, 147°39'W), 10 Jul 1952, C. P. Alexander (1♂; USNM). *Kenai Peninsula*: Arc Lake (3.2 km W Soldotna; 60°27'N, 151°06.3'W), 5 Jul 2006, D. and W. N. Mathis (1♀; USNM); Kenai Lake (60°20.5'N, 149°22.2'W; Primrose Campground), 31 Jul 2003, D. and W. N. Mathis (3♂, 3♀; USNM); Kenai River, Jim’s Landing (60°28.9'N, 150°06.9'W), 3 Aug 2002, D. and W. N. Mathis (3♂, 2♀; USNM); Moose Creek (60°30'N, 149°25'W), 27 Jul 1978, P. H. Arnaul, Jr. (1♂; CAS); Skilak Lake (60°26.3'N, 150°19.4'W), 3 Aug 2002, D. and W. N. Mathis (1♂, 2♀; USNM); Swanson River Road (60°42.8'N, 150°48.9'W), 13 Aug 2012, D. and W. N. Mathis (1♂; USNM). *Lake and Peninsula*: Savonoski, Naknek Lake (58°32'N, 155°19'W), 12-26 Jul 1919, 1932, A. Basinger (3♂, 3♀; USNM). *Matanuska-Susitna*: Eklutna (Knik Arm; 61°28.2'N, 149°21.4'W), 7 Aug 2002, D. and W. N. Mathis (23♂, 2♀; USNM); Glennallen (96.5 km W Glennallen; 61°55.6'N, 147°13.6'W), 7 Aug 2012, D. and W. N. Mathis (2♂, 4♀; USNM); Honolulu Creek, George Parks Highway A-178 (63°03.4'N, 149°35.5'W), 17 Jul 1978, P. H. Arnaud, Jr. (3♂, 1♀; CAS); Knik Lake, SW Wasilla (61°27.7'N, 149°43.9'W), 18 Jul 1978, P. H. Arnaud, Jr. (1♀; CAS). Knik River (61°27.8'N, 148°51.6'W), 5 Aug 2002, D. and W. N. Mathis (18♂, 6♀; USNM); Little Willow Creek (61°48.6'N, 150°05.8'W; 50 m), 25 Jul 2011, D. and W. N. Mathis (1♂, 1♀; USNM); Lucile Lake (61°34.2'N, 149°28.6'W; 100 m), 15 Aug 2012, D. and W. N. Mathis (1♂; USNM); Matanuska Flats (N Palmer; 61°16'N, 150°16'W), 17 Jul 1971, B. A. Foote (1♂; USNM); Palmer (Matanuska River; 61°36.5'N, 149°04.1'W), 16 Aug 2012, D. and W. N. Mathis (11♂, 3♀; USNM); Sheep Creek (61°58.3'N, 150°05'W; 55 m), 10 Aug 2011, D. and W.N. Mathis (5♂, 1♀; USNM); Talkeetna (62°18.9'N, 150°06.3'W), 4-10 Aug 2003, 2011, D. and W. N. Mathis (13♂, 1♀; USNM); Willow Creek (61°46.1'N, 150°04.2'W; 50 m), 10-26 Jul 2006, 2011, D. and W. N. Mathis (3♂, 1♀; USNM). *Nome (Census Area)*: Pilgrim Hot Springs (65°05.6'N, 164°55.6'W), 3 Aug 2012, D. and W. N. Mathis (1♂, 1♀; USNM); Snake River (11 km NW Nome; 64°33.9'N, 165°30.6'W), 2 Aug 2012, D. and W. N. Mathis (1♀; USNM). *Southeast Fairbanks Census Area*: Delta Junction (8 km S; 63°51.5'N, 145°44.6'W), 11 Aug 2003, D. and W. N. Mathis (1♂; USNM); Dry Creek Campground, Glenn Highway A-192 (63°39.2'N, 144°21.8'W), 3 Aug 1978, P. H. Arnaud, Jr. (1♀; CAS); Gardiner Creek Camp, Alaska Highway DC 1253 (62°51.5'N, 141°28'W), 5 Aug 1978, P. H. Arnaud, Jr. (13♂, 6♀; CAS); Gerstle River, Alaska Highway DC 1393 (64°03.4'N, 145°08.1'W), 9 Jul 1978, P. H. Arnaud, Jr. (1♂; CAS); Walker Fork Campground, Richardson Highway (64°04.6'N, 141°37.4'W), 31 Jul 1978, P. H. Arnaud, Jr. (2♂; CAS). *Valdez-Cordova (Census Area)*: Chitina (61°30.9'N, 144°26.2'W), 18 Jun 1953, W. C. Frohne (1♂, 2♀; WSU); Gulkana River (19.3 km N Glennallen; 62°16.1'N, 145°23.1'W), 6 Aug 2012, D. and W. N. Mathis (15♂, 6♀; USNM); Klutina River (mile 101; 61°57.2'N, 145°19.3'W; 315 m), 7 Aug 2012, D. and W. N. Mathis (5♂, 4♀; USNM); Lower Tonsina Valley (61°39.3'N, 144°39.5'W), 19 Aug 1953, W. C. Frohne (1♀; WSU); Tolsona Creek State Campground, Glenn Highway, A 173 (62°03.9'N, 145°59.8'W), 31 Jul 1978, P. H. Arnaud, Jr. (2♂, 1♀; CAS); Valdez (4.8 km N; 61°05.8'N, 146°14.6'W), 8 Jul 2006, D. and W. N. Mathis (1♀; USNM). *Yukon-Koyukuk (Census Area)*: Kanuti National Wildlife Refuge, Jim River (66°45.4'N, 151°21.1'W; Malaise trap), 4-6 Aug 2006 (3♂, 3♀; UAF); Kanuti National Wildlife Refuge, Jim River (66°39'N, 151°30.4'W; Malaise trap), 11 Aug 2006, L. Saperstein (1♂, 3♀; UAF); Nenana River, George Park Highway A-222 (64°33.9'N, 149°06.3'W), 17 Jul 1978, P. H. Arnaud, Jr. (4♂, 1♀; CAS); Yukon River at Dalton Highway (65°52.8'N, 149°43.2'W; 110 m), 4 Aug 2011, D. and W. N. Mathis (4♂; USNM).

*OREGON. Josephine*: Illinois River, W Fork (3 km S Cave Junction; 42°09.6'N, 123°39.5'W), 20 Jun 1974, P. H. Arnaud, Jr. (1♂; CAS).

#### Distribution

(Fig. 52). *Nearctic*: Canada (British Columbia, Northwest Territories, Quebec, Yukon Territory), United States (Alaska, Oregon, Washington).

#### Remarks.

This species is very similar and is probably closely related to the *Gymnoclasiopa cinerella* (Stenhammar) in the Old World but is distinguished from that species by the shape of structures of the male terminalia (see figures). From Nearctic congeners, this species is distinguished by the yellowish to slightly reddish maxillary palpi and the black scape and pedicel.

### Gymnoclasiopa tacoma

(Cresson)

http://species-id.net/wiki/Gymnoclasiopa_tacoma

Figs 53 –56

Ditrichophora tacoma Cresson 1924: 160; 1942: 119 [review]. Strickland 1946: 167 [list Alberta]. Wirth 1965: 739 [Nearctic catalog]. Cole 1969: 398 [fauna, western North America].
Gymnoclasiopa tacoma . Mathis and Zatwarnicki 1995: 178 [generic combination; world catalog].

#### Diagnosis.

**Figures 53–55.**
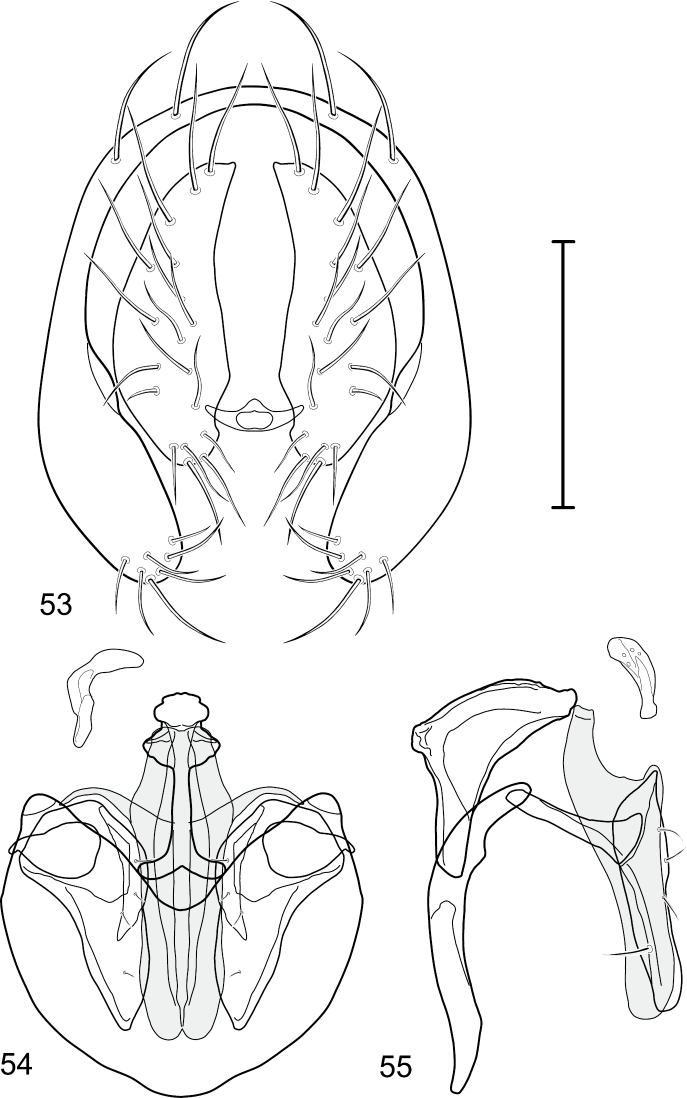
*Gymnoclasiopa tacoma* (Cresson) (USA. Washington. Pierce: Gig Harbor). **53** epandrium and cerci, posterior view **54** internal structures of male terminalia (aedeagus [shaded], phallapodeme, gonite, hypandrium), ventral view **55** same, lateral view. Scale bar = 0.1 mm.

This species is distinguished from congeners by the following combination of characters: Small to moderately small shore flies, body length 1.95–2.85 mm; generally black dorsally with moderately to sparse microtomentum . *Head*: Frons grayish black, moderately to sparsely microtomentose, subshiny. Scape black; pedicel black basally, apicoventrally yellow; basal flagellomere slightly darkened dorsally, otherwise yellow to reddish yellow, sometimes wholly so; arista bearing 5 dorsal rays. Facial microtomentum generally yellow to slightly brownish yellow; parafacial and gena grayish microtomentose, contrasted with gold-yellow color of face; gena moderately high, gena-to-eye ratio 0.13–0.18. Maxillary palpus yellow to red. *Thorax*: Mesonotum slightly grayish black to black, sparsely microtomentose, subshiny to shiny; pleural area largely like mesonotum, sometimes more grayish to tannish black. Wing hyaline; costal vein ratio 0.37–0.45; M vein ratio 0.55–0.60. Coxae, femora, and tibia grayish black to black; tarsi yellow, apical tarsomeres becoming darker, brownish yellow. *Abdomen*: Tergites Partially subshiny to mostly shiny black, very sparsely microtomentose. Male terminalia (Figs 53–55): Epandrium in posterior view (Fig. 54) as a broadly formed, inverted U with the base more narrowly formed, dorsal portion more thinly developed than lateral arms, lateral arms shallowly arched, enlarged ventrally, broadly rounded, setulae more clustered at ventral margin; cercus in posterior view (Fig. 53) elongate,semilunate, lateral margins arched, medial margins irregularly straight, setulae more clustered at ventral margin; aedeagus in lateral view (Fig. 55) slipper-like, base shallowly emarginate, tapered very gradually toward apex, apical half nearly parallel sided, apex moderately broadly rounded, in ventral view (Fig. 54) expanded laterally from narrow base on basal 1/3, thereafter to apex almost parallel sided, slightly tapered, apical margin shallowly incised medially, bilobed; phallapodeme in lateral view (Fig. 55) more or less irregularly triangular, extension toward hypandrium more elongate than angle towards aedeagal base, in ventral view (Fig. 54) I-shaped, apical crossbar robust, wider than basal crossbar, apical margin very shallowly emarginate; ejaculatory apodeme in lateral view shallowly comma-shaped, in ventral view L-shaped; postgonite in lateral view (Fig. 55) bar-like, only slightly tapered from base to apex, bearing 2-3 setulae along posterior margin and 1 setula subapically along anterior margin, in ventral view (Fig. 54) as an elongate triangle, wide basally, tapered to narrowly rounded apex, lateral margins shallowly emarginate laterally, arched medially; pregonite in lateral view (Fig. 55) moderately elongate, straight, expanded slightly aedeagal base, in ventral view (Fig. 54) lunate with apices pointed; hypandrium in ventral view (Fig. 54) robustly V-shaped, lateral margins shallowly curved, anterior margin rounded, posterior margin emarginate, widely V-shaped, in lateral view (Fig. 55) narrowly elongate, shallowly curved.

**Type material.** The neotype male of *Ditrichophora tacoma* Cresson, here designated, is labeled “WASH[INGTON].PierceCo. 3 mi. W.S.W. DuPont [47°05.6'N, 122°40'W], 9 Jun [handwritten] 1971[,] Wayne N. Mathis/NEOTYPE ♂ *Ditrichophora tacoma* Cresson, designated by Mathis & Zatwarnicki 2012 - USNM [red].” The neotype is double mounted (glued to a paper triangle), is in excellent condition, and is deposited in the USNM. There are also 46 specimens (24♂, 22♀; USNM) from the type locality, some with varying dates of collection (13 Apr-9 Jun 1971). DuPont is near the original type locality (Tacoma).

When Cresson (1924) described this species, he noted that the holotype was deposited in the collection of the University of Washington (Seattle). We were unsuccessful in locating this holotype at the Burke Museum, University of Washington (Rodney L. Crawford) or at other collections in the Northwest or where Cresson worked: Oregon State University (Christopher J. Marshall), Washington State University (Richard S. Zack), Academy of Natural Sciences of Philadelphia (Jon K. Gelhaus, Jason D. Weintraub). We are thus designating a neotype for this species, especially given the likelihood of a junior synonym (in the Old World) and the need for a primary type to stabilize the nomenclature of this species.

#### Type locality.

United States. Washington. Pierce: DuPont (4.8 km WSW; 47°05.6'N, 122°40'W).

#### Other specimens examined.

Nearctic. CANADA. *ALBERTA*. Edmonton (53°32.1'N, 113°29.4'W), 29 Apr 1924, O. Bryant (1♂; USNM).

*BRITISH COLUMBIA*. Spences Bridge (50°25'N, 121°21'W), 29 Aug 1951, A. H. Sturtevant (1♀; USNM). Vanderhoof (ca. 27.4 km E, Route 16; 53°53'N, 123°26'W; ditch), 7 Jun 1983, R. S. and V. L. Zack (1♂, 1♀; WSU).

*ONTARIO*. Ottawa (45°23.5'N, 75°38'W), 26 Apr 1922, C. H. Curran (1♀; ANSP).

*QUEBEC*. Hull (45°25.8'N, 75°42.8'W), 4 Jun 1923, C. H. Curran (2♂, 1♀; ANSP); Sainte-Anne-de-la-Pérade (46°35'N, 72°12'W), A. L. Melander (1♀; USNM).

UNITED STATES. *ALASKA. Anchorage*: Mirror Lake (61°25.7'N, 149°24.9'W), 5 Aug 2002, D. and W. N. Mathis (2♀; USNM). *Fairbanks North Star*: Chena Lake Recreation Area (64°47.6'N, 147°11.4'W), 10 Aug 2003, D. and W. N. Mathis (1♂, 2♀; USNM); Fairbanks (64°50.3'N, 147°43'W), 1 Jul 1921, J. M. Aldrich (4♀; ANSP, USNM); Fairbanks, Creamer’s Field (64°51.7'N, 147°44.3'W; 160 m), 3 Aug 2011, D. and W. N. Mathis (1♂; USNM). *Juneau*: Eagle Creek (58°31.6'N, 134°49'W), 21 Jul 2011, D. and W. N. Mathis (1♂; USNM). *Kenai Peninsula*: Homer (59°38.8'N, 151°31.5'W), 2 Aug 2002, D. and W. N. Mathis (1♀; USNM); Kasilof (11 km S; 60°14.8'N, 151°21.9'W), 2 Aug 2003, D. and W. N. Mathis (1♂; USNM); Kenai River, Jim’s Landing (60°28.9'N, 150°06.9'W), 3 Aug 2002, D. and W. N. Mathis (2♂, 1♀; USNM); Ninilchik (60°03'N, 151°40.2'W; beach), 2 Jul 2006, D. and W. N. Mathis (1♂, 3♀; USNM); Rainbow Lake (60°43.1'N, 150°49.1'W), 13 Aug 2012, D. and W. N. Mathis (1♀; USNM); Skilak Lake (60°26.3'N, 150°19.4'W), 3 Aug 2002, D. and W. N. Mathis (10♂, 2♀; USNM); Soldotna (6.5 km E; 60°30.5'N, 150°55.6'W), 1 Aug 2003, D. and W. N. Mathis (2♂, 1♀; USNM); Swanson River Landing (60°44.7'N, 150°48'W), 13 Aug 2012, D. and W. N. Mathis (8♂, 10♀; USNM). *Peninsula*: Savonoski, Naknek Lake (58°30.9'N, 155°31.8'W), 9-20 Jul 1919, A. J. Basinger (4♂, 3♀; USNM). *Matanuska-Susitna*: Eklutna (Knik Arm; 61°28.2'N, 149°21.4'W), 7 Aug 2002, D. and W. N. Mathis (3♂; USNM); Knik River (61°27.8'N, 148°51.6'W), 5 Aug 2002, D. and W. N. Mathis (4♂; USNM); Little Willow Creek (61°48.6'N, 150°05.8'W; 50 m), 25 Jul 2011, D. and W. N. Mathis (1♂; USNM); Lucile Lake (61°34.2'N, 149°28.6'W; 100 m), 15 Aug 2012, D. and W. N. Mathis (1♂, 1♀; USNM); Matanuska (61°32.5'N, 149°13.8'W; rotary trap), 28 Apr-21 May 1944, 1945, J. C. Chamberlin (1♂, 4♀; USNM); Palmer (Knik River; 61°31.2'N, 148°59.4'W), 6 Aug 2002, D. and W. N. Mathis (2♂; USNM); Pittman (61°35.5'N, 149°37.9'W), 14 Aug 2012, D. and W. N. Mathis (2♂; USNM); Sheep Creek (61°58.3'N, 150°05'W; 55 m), 10 Aug 2011, D. and W.N. Mathis (2♂, 2♀; USNM); Talkeetna (Susitna River; 61°19.4'N, 150°07.2'W; 120 m), 10 Aug 2011, D. and W.N. Mathis (♂, ♀; USNM); Willow Creek (61°46.1'N, 150°04.2'W; 50 m), 10 Jul-17 Aug 2006, 2011, 2012, D. and W. N. Mathis (22♂, 3♀; USNM). *Valdez-Cordova (Census Area)*: Chitina (61°30.9'N, 144°26.2'W), 18 Jun 1953, W. C. Frohne (1♀; WSU); Gulkana River (19.3 km N Glennallen; 62°16.1'N, 145°23.1'W), 9 Jul-7 Aug 2006, 2011, 2012, D. and W. N. Mathis (22♂, 17♀; USNM); Klutina River (mile 101; 61°57.2'N, 145°19.3'W; 315 m), 7 Aug 2012, D. and W. N. Mathis (3♂, 2♀; USNM). *Yukon-Koyukuk (Census Area)*: Yukon River at Dalton Highway (65°52.8'N, 149°43.2'W; 110 m), 4 Aug 2011, D. and W. N. Mathis (1♂; USNM).

*COLORADO. Gunnison*: Rocky Mountain Biological Laboratory (38°57.5'N, 106°59.4'W; 2900 m), 26 Aug 1961, D. L. Deonier (1♂, 4♀; USNM). *Rio Grande*: South Fork (37°40.2'N, 106°38.4'W; 2440 m), 20 Jun 1972, W. W. Wirth (2♂; USNM).

*IDAHO. Latah*: near Big Meadow Creek Recreation Area (11.25 km N Troy; 46°51'N, 116°44.7'W; 915 m; Malaise trap and sweeping), 13-31 Jul 1979, W. J. Turner (3♂, 2♀; WSU); Helmer (5 km S; 46°46.9'N, 116°28.2'W), 22 May 1971, W. J. Turner (1♂; USNM); Little Sand Creek, near Bonami Creek (25.75 km E Potlach; 46°54.9'N, 116°37.5'W; 884 m), 5 Aug 1979, W. Turner (1♀; WSU).

*MICHIGAN. Presque Isle*: (45°21.1'N, 83°29.3'W), 2 Jun 1951, R. R. Dreisbach (1♂; USNM).

*OREGON. Benton*: Corvallis (1.6 km SE, Willamette River; 44°31.7'N, 123°15.2'W), 4 Apr 1972, W. N. Mathis (1♀; USNM); Mary’s Peak (44°30.3'N, 123°33.1'W), 1 Aug 1975, W. N. Mathis (2♀; USNM).

*WASHINGTON. Ferry*: Colville National Forest, Republic (29 km E; 48°38.9'N, 118°42'W), 19 Jul 1970, P. W. Oman (1♀; USNM). *Okanogan*: Okanogan National Forest, Wauconda (11.25 km SE, Highway 30; 48°43.7'N, 118°57.9'W), 28 Jul 1973, W. J. Turner (2♂; WSU). *Pierce*: Mount Rainier National Park, Christine Falls (above; 46°46.9'N, 121°46.8'W; 1125 m), 11–13 Aug 1977, R. S. Zack (2♂; USNM, WSU); Mount Rainier National Park, Comet Falls Trail above Van Trump Creek (46°46.4'N, 121°46.8'W; 1370 m), 11 Aug 1977, R. S. Zack (1♂; WSU); Mount Rainier National Park, Ramparts (46°45.5'N, 121°48.9'W), 1 Aug 1922, A. L. Melander (1♀; WSU); Mount Rainier National Park, Shadow Lake near Sunrise (46°54.7'N, 121°39.4'W), 10 Aug 1977, R. S. Zack (2♂, 7♀; WSU); Mount Rainier National Park, St. Andrews Creek (19.3 km NE Sunshine Point campground; 46°50'N, 121°53'W), 26 Jun 1979, R. S. Zack (2♀; WSU); Mount Rainier National Park, Sunshine Point (6 km NE; 46°44.6'N, 121°55.6'W; 914 m; campground), 18 Jun-8 Aug 1979, R. S. Zack (5♂, 10♀; WSU); Mount Rainier National Park, Westside Road (5 km N; 46°51'N, 121°54.9'W), 12 Aug 1977, R. S. and V. L. Zack (1♂; USNM); Mount Rainier National Park NP, Westside Road, near Puyallup River (46°56'N, 121°35'W; 1066 m), 12 Aug 1977, R. S. Zack (1♀; WSU); Mount Rainier National Park, White River (46°59.6'N, 121°42.6'W), 28 Aug 1934, A. L. Melander (1♂; ANSP); Mount Rainier National Park, White River Campground (46°54.1'N, 121°38.5'W; 1340 m), 9-10 Aug 1977, W. J. Turner, R. S. Zack (1♂, 3♀; WSU); Tacoma (47°15.2'N, 122°26.7'W), 27 Aug 1911 (1♀; paratype; ANSP). *Snohomish*: Index (47°49.2'N, 121°33.3'W), 2 Aug 1917, A. L. Melander (1♂; ANSP); Verlot (48°05.4'N, 121°46.6'W), 3 Aug 1951, A. H. Sturtevant (2♂, 1♀; USNM).

*WYOMING. Fremont*: Lander (42°50'N, 108°43.8'W), 16 Aug 1950, A. H. Sturtevant (2♂; USNM).

#### Distribution

**Figure 56.**
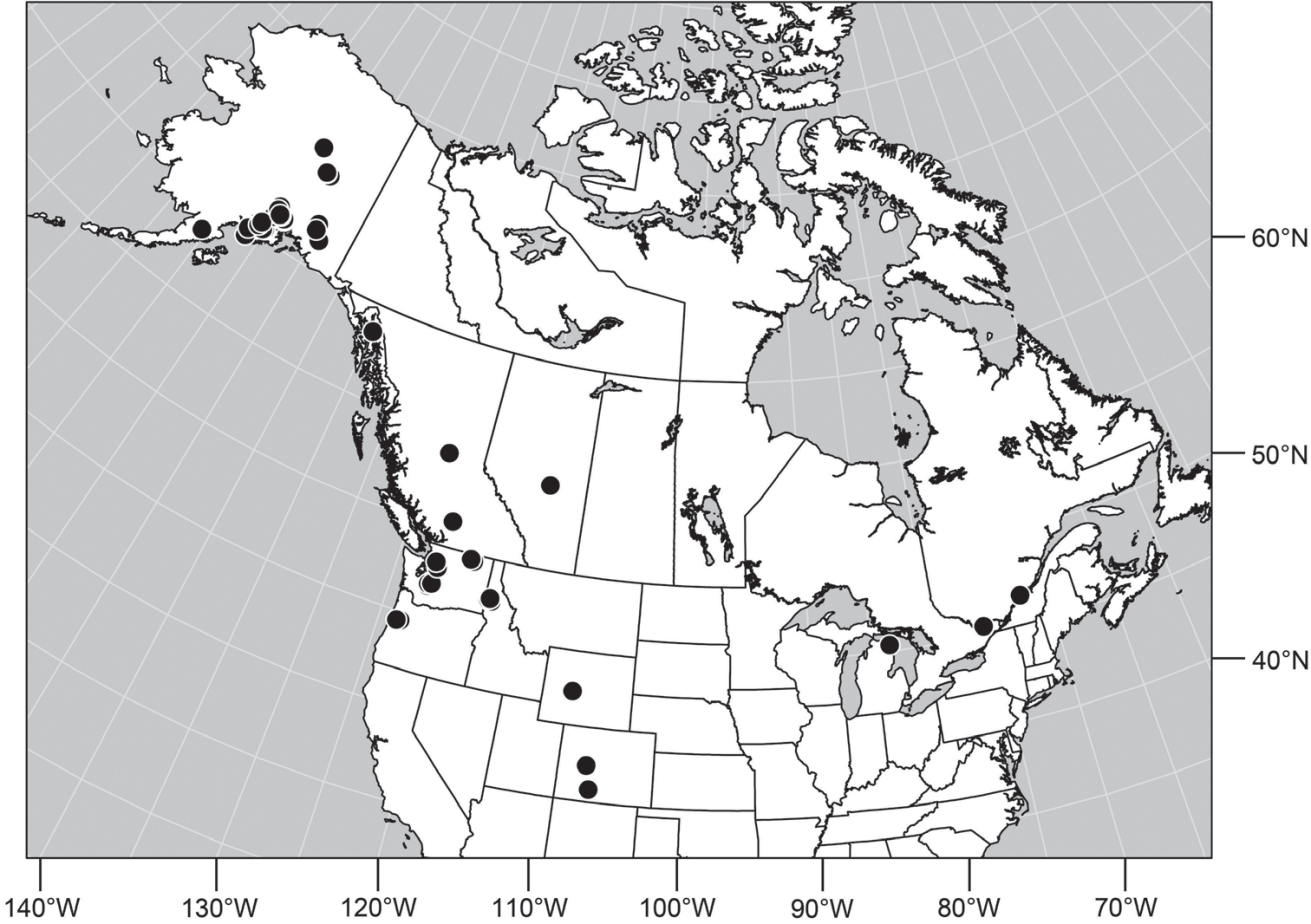
Distribution map of *Gymnoclasiopa tacoma* (Cresson).

(Fig. 56). *Nearctic*: Canada (Alberta, British Columbia, Ontario, Quebec), United States (Alaska, Colorado, Idaho, ?Maine [literature record, not confirmed; Cresson 1924: 160], Michigan, Oregon, Washington, Wyoming). *Palearctic*: Finland, Japan (Hokkaido), Mongolia (Töv).

#### Remarks.

This species is relatively widespread in the Nearctic Region and in the Old World, we have examined specimens from Finland, Japan, and Mongolia.

This species is distinguished from congeners by the yellowish to yellowish red antennae and maxillary palpi coupled with the black fore- and midtibiae, which are similar in color to the femora.

##### Nearctic species excluded from *Gymnoclasiopa*

In our world catalog (Mathis and Zatwarnicki 1995), we transferred two Nearctic species, *Ditrichophora cana* Cresson and *Ditrichophora canifrons* Cresson, from *Ditrichophora* to *Gymnoclasiopa*. This transfer was based on external characters. We have now examined structures of the male terminalia of these two species, and from this evidence, we suggest that their proper placement is in *Ditrichophora*. Like other species in *Ditrichophora*, these two species have a rounded epandrium, a single gonite (probably the postgonite) that is not divided into pre- and postgonites, and a phallapodeme that is somewhat Y-shaped in lateral view. Details concerning these two species, including diagnoses, follow.

### Ditrichophora cana

Cresson stat. rev.

http://species-id.net/wiki/Ditrichophora_cana

Fig. 57

Ditrichophora cana Cresson 1940: 7; 1942: 121 [review]. Wirth and Stone 1956: 467 [key, California]. Wirth 1965: 739 [Nearctic catalog]. Cole 1969: 398 [fauna, western North America].
Gymnoclasiopa cana . Mathis and Zatwarnicki 1995: 175 [generic combination; world catalog].

#### Diagnosis.

This species is distinguished from congeners by the following combination of characters: Moderately small to medium-sized shore flies, body length 2.40–3.60 mm; head and thorax generally microtomentose gray dorsally, abdomen subshiny to shiny black. *Head*: Frons moderately microtomentose, cinereous to whitish; proclinate fronto-orbital setae 2, length of anterior seta about 1/2 that of posterior seta, inserted far anteriad, distance between proclinate setae subequal to that between posterior seta and medial vertical seta. Antenna generally black, especially scape and pedicel; arista bearing 6–7 dorsal rays. Face rather flat; antennal grooves, especially ventral margins, poorly defined, not conspicuous; face slightly whitish gray dorsally, dorsal portion shallowly carinate between shallow antennal grooves, thinly, microtomentose, becoming blackish, less microtomentose ventrally; facial setae inserted close to parafacials, aligned vertically; gena short, less than height of basal flagellomere; gena-to-eye ratio 0.15–0.16. Maxillary palpus black. *Thorax*: Mesonotum moderately microtomentose, cinereous, similar to frons, not shiny; pleural areas from ventral notopleural suture ventrad black, contrasted with whitish gray mesonotum, similar to black abdominal tergites. Wing lacteous, more so in males; costal section II conspicuously longer than costal section III; costal vein ratio 0.47–0.51; M vein ratio 0.52–0.56; halter stem yellowish; knob white to whitish yellow. Femora black; tibiae black, including basal and apical extremities black; foretarsus black dorsally, yellowish ventrally; mid- and hindtarsi mostly yellowish orange. *Abdomen*: Tergites subshiny to shiny, black. Male terminalia: Epandrium in posterior view as an inverted U, narrowed dorsally, abruptly so medially with moderately deep incision, in lateral view widest subventrally with ventral margin rounded and bearing cluster of longer setulae, these becoming longer toward posteroventral angle; cercus in posterior view uniformly semilunate with dorsomedial margin more narrowly pointed; aedeagus in lateral view longer than wide, as anterior and posterior structures, anterior portion longer than posterior portion and with a long flap folded back on itself (as in *Pectinifer aeneus* (Cresson)), in ventral view robust, narrow ventrally; phallapodeme in lateral view with elongate, irregularly triangular keep, processes at either end about equal in length, in ventral view robustly T-shaped with thick stem, base as wide as cross bar; gonite (probably the postgonite) elongate, wide basally, narrowed to elongate, narrow, apically curved process, at midlength with a digitiform, pointed perpendicular to plane of gonite; hypandrium in lateral view bowl shaped, posterior portion slightly more extended, in ventral view with anterior margin broadly curved and deeply emarginate.

#### Type material.

The holotype male of *Ditrichophora cana* Cresson is labeled “Ilwaco WASH July 1917 ALMelander/TYPE Ditrichophora CANA E. T. Cresson, Jr. [red; species number and name handwritten].” The holotype is double mounted (minuten pin in a rectangular card), is in fair condition (head dislodged and in an attached microvial), and is deposited in the ANSP (6552).

#### Type locality.

United States. Washington. Pacific: Ilwaco (46°18.5'N, 124°02.6'W).

Other Specimens Examined. *CALIFORNIA. Humboldt*: Orick (41°17.2'N, 124°03.6'W), 21 Jun 1935, A. L. Melander (1♂, 1♀; ANSP).

*OREGON*. *Curry*: Gold Beach (42°24.4'N, 124°25.3'W), 27 Jul 1951, A. H. Sturtevant (1♂, 2♀; USNM). *Douglas*: Elkton (43°38.2'N, 123°33.9'W), 28 Jul 1951, A. H. Sturtevant (1♂; USNM). *Multnomah*: Benson Park (45°34.5'N, 122°07.7'W), 24 Jun 1935, A. L. Melander (1♂; ANSP).

*WASHINGTON. Clallam*: Bogachiel Way, Forks (47°56.3'N, 124°24.6'W), 1 Aug 1951, A. H. Sturtevant (4♂, 3♀; USNM; Sequim (48°04.8'N, 123°06.1'W), 2 Aug 1951, A. H. Sturtevant (8♂, 2♀; USNM). *Lewis*: Toledo (46°26.4'N, 122°50.8'W), 27 Jun 1935, A. L. Melander (1♂, 1♀; ANSP). *Pacific*: Ilwaco (46°18.5'N, 124°02.6'W), 8 Jun-Jul 1917, 1925, A. L. Melander (4♂, 3♀; ANSP, USNM); Seaview (46°20.1'N, 124°03.3'W), A. Spuler (2♂; USNM). *Pierce*: Tacoma (47°15.2'N, 122°26.7'W), 23 Jul 1915, A. L. Melander (1♀; ANSP). *Snohomish*: Everett (47°58.7'N, 122°12'W), 4-6 Jul 1924, A. L. Melander (7♂, 3♀; ANSP; USNM).

#### Distribution 

**Figure 57.**
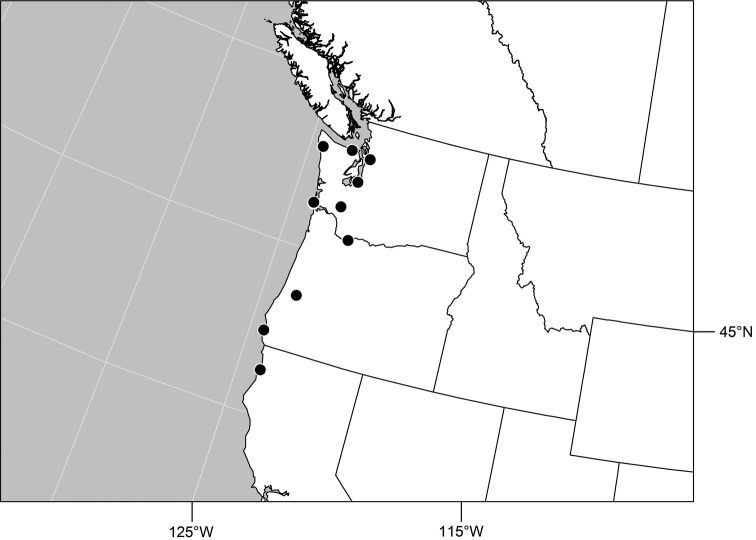
Distribution map of *Ditrichophora cana* Cresson.

(Fig. 57). Nearctic: United States (California, Oregon, Washington).

#### Remarks.

Although similar externally to *Ditrichophora canifrons*, the structures of the male terminalia of this species are remarkably different and appear to be more similar to *Pectinifer aeneus* (Cresson) (Zatwarnicki and Mathis 2001: 44), especially the elongated and robust aedeagus with an apical flap that folds back on itself. From *Ditrichophora canifrons*, this species is distinguished by the blackish antenna and entirely black foretibia.

### Ditrichophora canifrons

Cresson stat. rev.

http://species-id.net/wiki/Ditrichophora_canifrons

Fig. 58

Ditrichophora canifrons Cresson 1926: 250; 1942: 121 [review]. Wirth 1965: 739 [Nearctic catalog]. Steyskal 1972: 129 [habitat on tree trunks].
Gymnoclasiopa canifrons . Mathis and Zatwarnicki 1995: 176 [generic combination, world catalog]. Chandler 2012: 17 [list, Maine].

#### Diagnosis.

This species is distinguished from congeners by the following combination of characters: Small to medium-sized shore flies, body length 1.85–3.25 mm; head and thorax generally microtomentose gray dorsally, abdomen subshiny to shiny black. *Head*: Frons moderately microtomentose, cinereous to whitish; proclinate fronto-orbital setae 2, length of anterior seta about 1/2 that of posterior seta, inserted far anteriad, distance between proclinate setae subequal to that between posterior seta and medial vertical seta. Antenna generally yellowish orange (sometimes apex of basal flagellomere and scape darkened); arista bearing 7–8 dorsal rays. Face rather flat; antennal grooves, especially ventral margins, poorly defined, not conspicuous; face slightly whitish gray dorsally, dorsal portion shallowly carinate between shallow antennal grooves, thinly, microtomentose, becoming blackish, less microtomentose ventrally; facial setae inserted close to parafacials, aligned vertically; gena short, less than height of basal flagellomere; gena-to-eye ratio 0.08–0.09. Maxillary palpus black. *Thorax*: Mesonotum moderately microtomentose, cinereous, similar to frons, not shiny; pleural areas from ventral notopleural suture ventrad black, contrasted with whitish gray mesonotum, similar to black abdominal tergites. Wing lacteous; costal section II conspicuously longer than costal section III; costal vein ratio 0.47–0.65; M vein ratio 0.54–0.57; halter stem yellowish tan to yellow, knob yellowish white to white. Femora black; tibiae black except for basal and apical extremities black; tarsi mostly yellowish orange. *Abdomen*: Tergites subshiny to shiny, black. Male terminalia: Epandrium in posterior view as an inverted U, narrowed dorsally, each lateral arm shallowly curved; cercus in posterior view semilunate with dorsomedial, narrow extension; aedeagus in lateral view longer than wide, truncate apically, in ventral view as wide as long, shallowly and angularly emarginate apically; phallapodeme in lateral view with elongate, narrow, process to base of aedeagus, and much shorter, digitiform process toward hypandrium, 1/3 length of longer process, in ventral view robustly T-shaped with thick stem, base as wide as cross bar; gonite (probably the postgonite) elongate, wide basally, narrowed to elongate, narrow process bearing 2 posterior setulae and 1 anterior, subapical setula; hypandrium in lateral view bowl shaped, posterior portion slightly more extended, in ventral view with anterior margin broadly curved and deeply emarginate.

#### Type material.

The holotype male of *Ditrichophora canifrons* Cresson is labeled “Jack Run, Allegheny, VI,14,08,Pa./♂/302/TYPE No. 6301 Ditrichophora CANIFRONS E T Cresson, Jr. [red; species number and name handwritten].” The holotype is double mounted (minuten pin in a rectangular card), is in excellent condition (some cephalic setae missing or misoriented), and is deposited in the ANSP (6301).

**Type locality.** United States. Pennsylvania. Allegheny: Jacks Run (40°29'N, 80°03'W).

#### Other specimens examined.

Canada. *QUEBEC*: Quebec City (46°48.2'N, 71°14.6'W), 5 Aug 1930, A. L. Melander (1♂; ANSP).

United States. *MAINE*. Hancock: Schoodic Peninsula (SERC Campus; 44°20.5'N, 68°03.7'W; lot 95), 16 Jul 2006, D. and W. N. Mathis (1♀; USNM).

*MARYLAND. Montgomery*: Bethesda (38°58.8'N, 77°06'W), 14 Jun-25 Aug 1965, 1971, 1974, G. C. Steyskal (16♂, 1♀; USNM); Cabin John (38°58.5'N, 77°09.5'W), 25 Jul 1972, G. C. Steyskal (19♂; USNM); Rockville (39°05.1'N, 77°09.2'W), 30 May 1969, G. C. Steyskal (1♀; USNM).

*NEW YORK. Ulster*: Beaver Kill (42°06.7'N, 73°59.2'W), 12 Aug 1909, E. T. Cresson, Jr. (1♂; ANSP).

*NORTH CAROLINA. Mitchell*: Penland (35°55.8'N, 82°06.7'W; 915 m), 17 Jun 1957, G. C. Steyskal (2♀; USNM).

*OHIO. Lawrence*: Vesuvius Lake (38°34.8'N, 82°37.5'W), 23 Aug 1974, J. Regensburg (1♀; USNM). *Preble*: Hueston Woods (39°26.9'N, 84°45'W), 25 Jun 1975, J. Regensburg (1♂, 1♀; USNM); Rush Run Wilderness Area (39°34.6'N, 84°37.9'W), 1 Jul 1979, J. Regensburg (1♀; USNM). *Wayne*: Rittman Salt Works (40°58.2'N, 81°45.7'W), 30 Jun 1977, B. A. Steinly (1♂; USNM).

*PENNSYLVANIA. Allegheny*: Jacks Run (40°29'N, 80°03'W), 14 Jun 1908 (5♂, 4♀; ANSP). *Mifflin*: Lewiston (40°33.6'N, 77°38'W), 7 Jun 1940, A. L. Melander (1♀; USNM). *Wilkes-Barre*: Mineral Spring (41°15.3'N, 75°50.5'W), 5 Sep 1927, A. L. Melander (1♂; USNM).

*TENNESSEE. Sevier*: Arch Rock (35°38.1'N, 83°26.3'W), 28 Jun 1941, A. L. Melander (4♂, 5♀; USNM); Chimneys (35°38.2'N, 83°29.3'W), 25 Jun 1941, A. L. Melander (4♀; USNM).

*VIRGINIA. Fairfax*: Fairfax (38°50.5'N, 77°18.5'W), Jul 1954, M. R. Wheeler (1♂, 3♀; USNM); Lake Barcroft (38°50.9'N, 77°09.4'W), 28 May 1977, W. N. Mathis (12♂, 13♀; USNM); Turkey Run (mouth; 38°57.9'N, 77°09.4'W), 21 Jun 2006, W. N. Mathis (1♀; USNM). *Independent City*: Falls Church (38°52.9'N, 77°10.3'W), 13 Jul 1954, W. W. Wirth (1♀; USNM).

*WEST VIRGINIA. Hardy*: Lost River State Park (38°55.6'N, 78°53.6'W), 19 Jun 1977, L. V. Knutson (2♀; USNM); Lost River State Park (38°53.8'N, 78°55.7'W; 615 m), 20 Jun-13 Jul 2007, D. and W. N. Mathis (1♂, 9♀; USNM). *Pocahontas*: Tea Creek (Right Fork; 38°20'N, 80°9.9'W), 29 Jul 1982, O. S. Flint, W. N. Mathis (4♂, 5♀; USNM). *Ritchie*: North Bend State Park (39°13.4, 81°06.6'W), 23 Jun 1970, G. C. Steyskal (1♂; USNM).

#### Distribution

**Figure 58.**
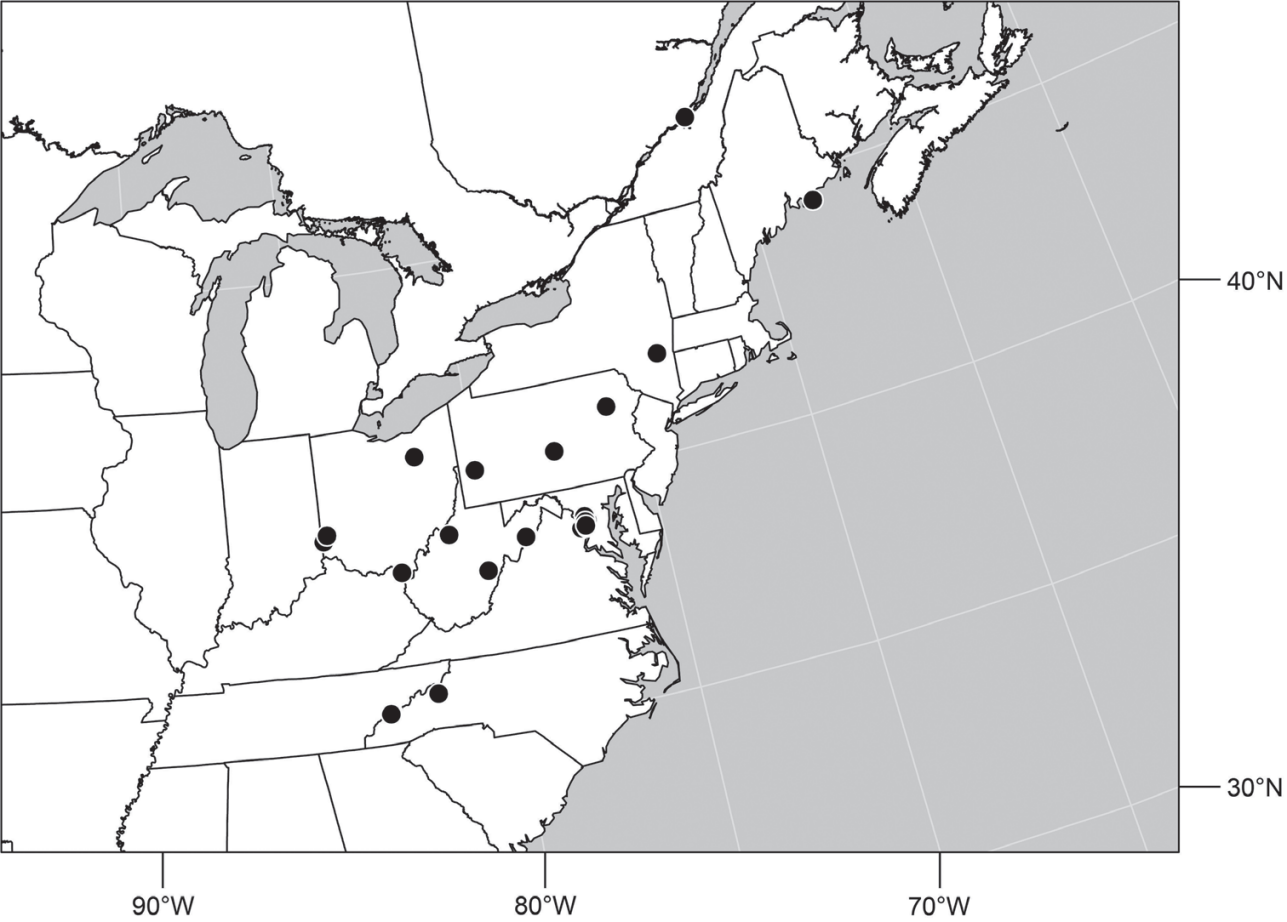
Distribution map of *Ditrichophora canifrons* Cresson.

(Fig. 58). *Nearctic*: Canada (Quebec), United States (Maine, Maryland, New York, North Carolina, Ohio, Pennsylvania, Tennessee, Virginia, West Virginia).

#### Remarks.

Although uncommon, this species is widespread in the Mid-Atlantic states, occurring along the coastal plain through the Piedmont and Blue Ridge to the Alleghany.

The mostly yellowish antenna, especially the pedicel in lateral view (all yellow except for the dorsum), and apex of the foretibia distinguish this species from *Ditrichophora cana*. Structures of the male terminalia, especially the shape of the aedeagus, as described above, are also diagnostic.

## Supplementary Material

XML Treatment for Discocerinini

XML Treatment for Gymnoclasiopa

XML Treatment for Gymnoclasiopa argyrostoma

XML Treatment for Gymnoclasiopa bella

XML Treatment for Gymnoclasiopa bohemanni

XML Treatment for Gymnoclasiopa chiapas

XML Treatment for Gymnoclasiopa grecorum

XML Treatment for Gymnoclasiopa matanuska

XML Treatment for Gymnoclasiopa parilis

XML Treatment for Gymnoclasiopa pulchella

XML Treatment for Gymnoclasiopa subnubila

XML Treatment for Gymnoclasiopa tacoma

XML Treatment for Ditrichophora cana

XML Treatment for Ditrichophora canifrons
